# Total Synthesis of the Tetracyclic Pyridinium Alkaloid *epi*‐Tetradehydrohalicyclamine B

**DOI:** 10.1002/anie.202209651

**Published:** 2022-09-02

**Authors:** Andrew G. Dalling, Georg Späth, Alois Fürstner

**Affiliations:** ^1^ Max-Planck-Institut für Kohlenforschung 45470 Mülheim/Ruhr Germany

**Keywords:** Alkaloids, Alkyne Metathesis, Chemoselectivity, Dual Catalysis, Photoredox Catalysis

## Abstract

The first total synthesis of a tetracyclic marine pyridinium alkaloid hinged on recent advances in chemoselectivity management: While many classical methods failed to afford the perceptively simple pyridine‐containing core of the target, nickel/iridium photoredox dual catalysis allowed the critical C−C bond to be formed in good yield. Likewise, ring closing alkyne metathesis (RCAM) worked well in the presence of the unhindered pyridine despite the innately Lewis acidic Mo(+6) center of the alkylidyne catalyst. Finally, an iridium catalyzed hydrosilylation was uniquely effective in reducing a tertiary amide without compromising an adjacent pyridine and the lateral double bonds; this transformation is largely without precedent. The second strained macrocycle enveloping the core was closed by intramolecular N‐alkylation with formation of the pyridinium unit; the reaction proceeded site‐ and chemoselectively in the presence of an a priori more basic tertiary amine.

## Graphical abstract

The conquest of a polycyclic marine pyridinium alkaloid was only made possible by the remarkable chemoselectivity profile of contemporary transition metal catalysts that operate in the presence of basic sites. Ni/Ir photoredox catalysis, Mo‐alkylidynes for alkyne metathesis, and an Ir‐catalyzed lactam‐selective hydrosilylation stand out in this regard.

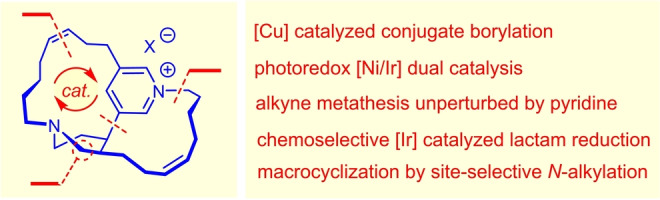

## Introduction

**Scheme 1:**
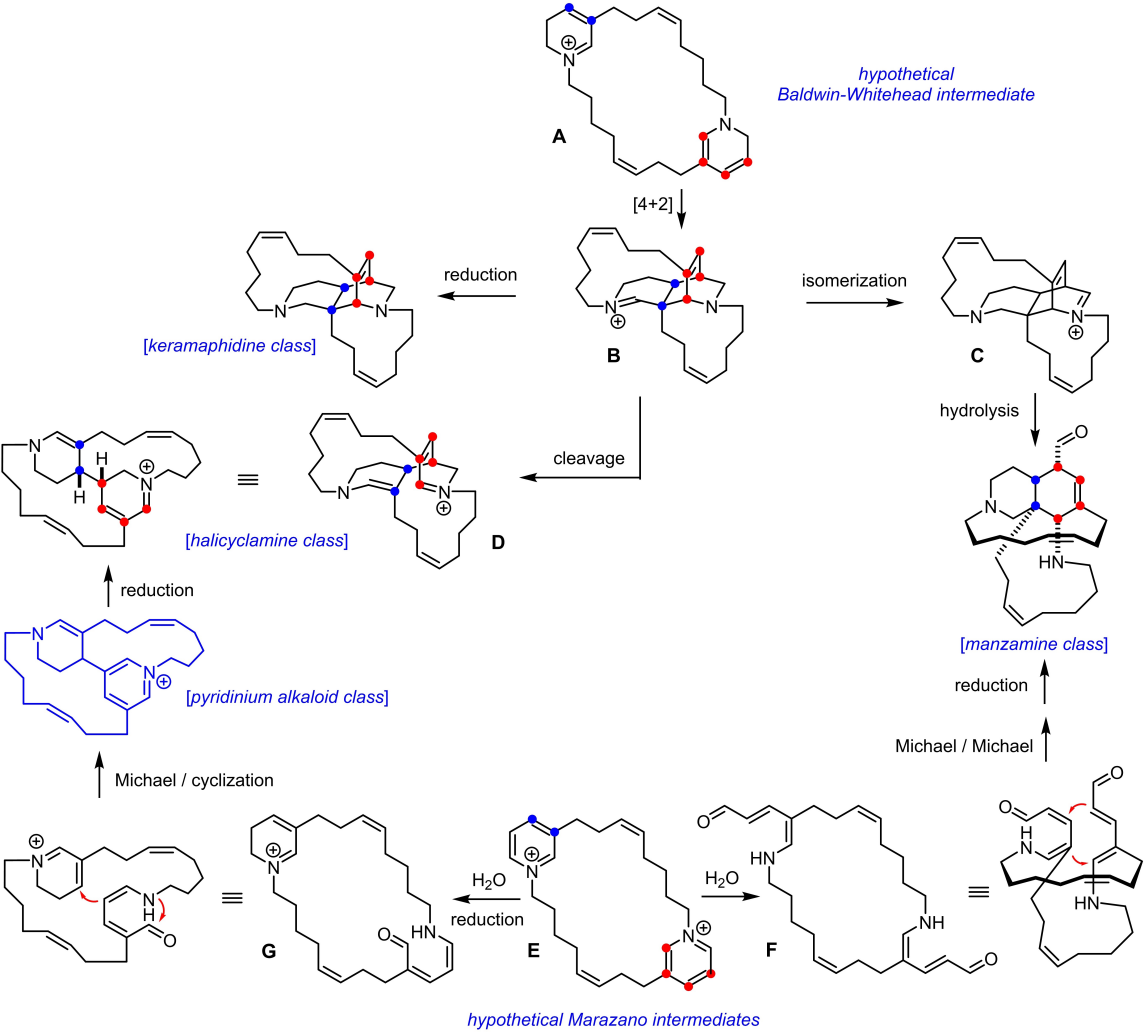
Summary of the two proposed biosynthesis pathways converting (partly reduced) 3‐alkylpyridine units into polycyclic alkaloids.

Two related yet distinct biosynthesis pathways were proposed in the literature to explain the genesis of an extraordinary suite of marine alkaloids from (partly reduced) 3‐alkylpyridine precursors. Of them, the “Baldwin‐Whitehead postulate” became particularly famous, which centers upon a transannular Diels–Alder cycloaddition of an intermediate of type **A** with formation of iminium ion **B** in the first place (Scheme 1).^[1]^ Simple reduction connects **B** to a prominent class of pentacyclic products such as keramaphidine and congeners,^[2]^ whereas isomerization to the new iminium species **C** followed by hydrolysis opens entry into the manzamine series. Moreover, cleavage of the C−C bond at one of the bridgeheads of **B** via a retro‐Mannich‐type reaction gives **D** and explains the formation of the large set of tetracyclic alkaloids of the halicyclamine class.[^3^, ^4^] Despite considerable efforts, however, all attempts at emulating the triggering transannular Diels–Alder reaction in the laboratory were met with limited success, furnishing—at best—0.2–0.3 % of the expected cycloadducts.[^5^, ^6^]

The circumstantial evidence for the alternative biosynthesis pathway proposed by Marazano and co‐workers is arguably stronger. These authors advocated for the intervention of 5‐amino‐2,4‐pentadienal derivatives such as **F** or **G** as key intermediates, which are thought to evolve via (transannular) 1,4‐conjugate additions either into the tetracyclic or pentacyclic estate as shown in Scheme 1.^[7]^ The critical C−C bond formation, for example **G**→**D**, is accompanied by the generation of a pyridinium ring, which may get (partly or fully) reduced once the core is formed. Several biomimetic studies provide strong evidence that all of the proposed key steps are feasible and facile.^[8]^ The isolation of several alkaloids comprising an intact pyridinium subunit lends further credence to this scenario.[^9^, ^10^]

**Figure 1:**
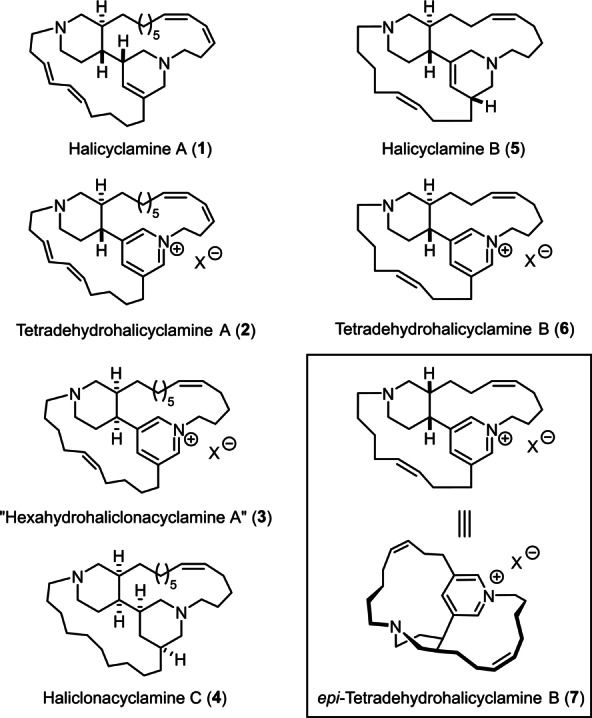
Representative members of the tetracyclic “halicyclamine” alkaloid family and the related pyridinium series; the perspective drawing of **7** shows the dominant strained conformer.

Many alkaloids of this lineage soon became prominent synthetic targets,[^11^, ^12^] except for the members of the tetracyclic series which attracted surprisingly little attention.[^13^, ^14^] They differ from each other in terms of size, unsaturation and substitution of the enveloping macrocycles, and in the relative and absolute configuration of the heterocyclic core (Figure 1).[^3^, ^4^, ^15^, ^16^, ^17^] They exhibit a broad and diverse biological activity profile as cytotoxic, antifungal and antibacterial agents; particularly noteworthy is the fact that halicyclamine A (**1**)^[3]^ as well as the pyridinium derivative hexahydrohaliclonacyclamine A (**3**) inhibit the growth of *Mycobacterium tuberculosis* H37Ra under both aerobic and hypoxic conditions.[^18^, ^19^] Because such dual effectiveness against the active as well as the dormant state of the strain is highly desirable, these alkaloids and their relatives deserve closer examination in the quest for new anti‐tuberculosis drugs.

## Results and Discussion

Intrigued by this outlook, we extended our program on macrocyclic natural products^[20]^ to this potentially relevant but largely ignored family. For strategic reasons, the tetracyclic pyridinium alkaloids were deemed the appropriate focal point for the first foray, none of which had ever been made before. These compounds are particularly scarce despite their presumed biosynthetic primacy (see above). Moreover, they represent the top of the synthetic hierarchy: for the ease of reduction of a pyridinium ring, conquest of **2**, **3**, **6** or **7** might eventually bring (partly) saturated siblings such as **1**, **4** and **5** (and/or isomers thereof) into reach. Other late‐stage molecular editing exercises are equally conceivable as part of more detailed future studies into structure/activity relationships.

**Scheme 2:**
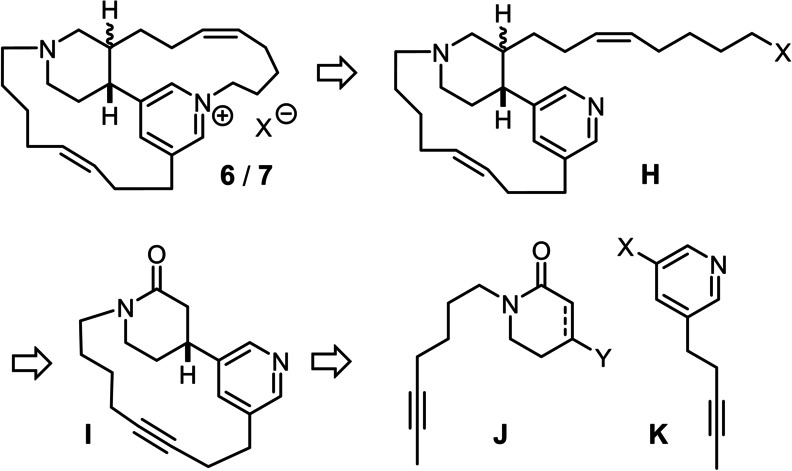
Retrosynthetic analysis.

Specifically, we opted for tetradehydrohalicyclamine B (**6**) and its *cis*‐configured epimer **7** as our principle targets, which had been isolated from marine sponges of the genus *Acanthostrongylophora ingens* collected off the cost of Sulawesi, Indonesia, by two independent groups. One team only found **6** (and its reduced sibling **5**) in the sponge specimen subjected to extraction; these compounds were described as moderately potent proteasome inhibitors but were not tested for anti‐tubercular activity.^[21]^ Interestingly, the other isolation team obtained an inseparable mixture of the two epimers **6** and **7**, with the latter being the major constituent (**6** : **7**≈35 : 65).^[22]^ For this complication, structure elucidation by spectroscopic means was supplemented by extensive computational studies.^[23]^ These data forecast considerable synthetic challenges in that the 13‐membered cycle of **7** is very rigid.^[24]^ The strain is partly transmitted by pyramidal inversion of the stereogenic N‐atom to the annellated 1,3,4‐trisubstituted piperidine ring, which favors a twist‐boat over an ordinary chair conformation (Figure 1).^[22]^ In addition to these structural issues, a successful synthetic venture must account for the reactive nature of the central pyridinium salt, which is amenable, inter alia, to reduction, nucleophilic attack, hydrolysis, and facile ring opening.^[25]^ Therefore it seemed advisable to carry the quarternization out as late as possible; to this end, we planned to resort to intramolecular N‐alkylation as a way of crafting the strained 13‐membered ring in the final stages of the synthesis (Scheme 2). In this case, however, the reaction has to take place in the presence of the more nucleophilic tertiary amine of the adjacent piperidine, which renders the maneuver delicate;^[26]^ at the same time, all transformations leading to the required precursor **H** must be compatible with one or both of these basic entities. Under this proviso, we opted for ring closing alkyne metathesis (RCAM) followed by semi‐reduction of the triple bond for the formation of the other macrocycle,[^27^, ^28^, ^29^] since molybdenum alkylidynes endowed with silanolate ligands are remarkably tolerant towards many different donor sites,[^13^, ^30^, ^31^, ^32^] most notably complexes with a tripodal silanolate ligand framework (“canopy catalysts”).[^33^, ^34^, ^35^, ^36^] The projected current application provides a stringent test for this notion.[^2^, ^37^]

**Scheme 3:**
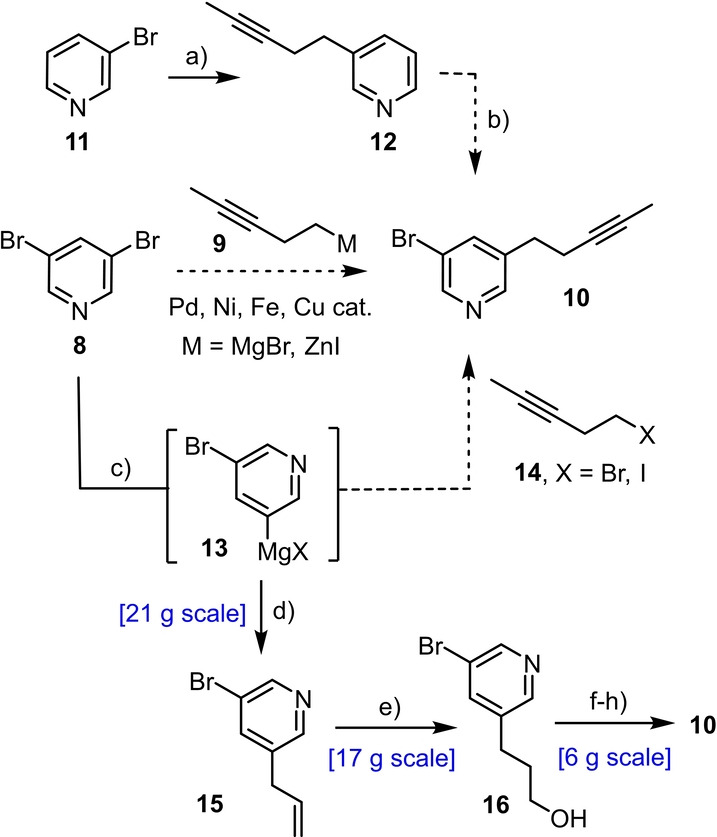
a) (i) **14** (X=I), Zn, DMF, 55 °C; (ii) Pd_2_(dba)_2_ (5 mol %), RuPhos (10 mol %), DMF, 50 °C, 67 %; b) (i) B_2_pin_2_, [Ir(cod)OMe]_2_ (5 mol %), 4,4′‐di‐*tert*‐butyl‐2,2′‐dipyridine (10 mol %), THF, reflux; (ii) CuBr_2_, MeOH, H_2_O, 80 °C; c) *i*PrMgCl⋅LiCl, THF, −15 °C; d) allyl bromide, CuCN⋅2 LiCl (1 mol %), 0 °C, 98 %; e) (i) BF_3_⋅OEt_2_, then 9‐H‐9‐BBN, THF, 0 °C→RT; (ii) tmeda, H_2_O_2_, NaOH, 0 °C→RT, 75 %; f) Dess–Martin periodinane, CH_2_Cl_2_, 0 °C, 95 %; g) MeC(O)C(N_2_)P(O)(OMe)_2_, K_2_CO_3_, MeOH, 90 %; h) (i) LiHMDS, THF, −78 °C; (ii) MeI, −78 °C→RT, 81 %; 9‐BBN=9‐borabicyclo[3.3.1]nonane; cod=1,5‐cyclooctadiene; dba=dibenzylideneacetone; RuPhos=2‐dicyclohexylphosphino‐2′,6′‐diisopropoxybiphenyl; tmeda=tetramethylethylenediamine; LiHMDS=lithium hexamethyldisilazide.

A scalable route to the required core fragment, though perceptively simple, proved surprisingly difficult to establish for more than one reason (Scheme 3). Specifically, direct cross coupling of pent‐3‐ynylzinc (magnesium) reagents **9** with 3,5‐dibromopyridine (**8**) under palladium, nickel, copper, or iron catalysis failed to afford manageable quantities of the desired mono‐substitution product **10**.[^38^, ^39^] While the analogous reaction with 3‐bromopyridine (**11**) proceeded uneventfully, attempted borylation/bromination of the resulting product **12** was also unsuccessful.^[40]^ Likewise, several attempts at alkylating the Grignard reagent **13** derived from **8** with the primary halide **14** (X=Br, I) in the presence or absence of (sub‐ or super‐stoichiometric) CuCN were to no avail either. This result is all the more surprising since the analogous reaction with allyl bromide as the electrophile proceeded almost quantitatively to give **15** even on >20 g scale.^[41]^ We therefore chose to elaborate this well accessible compound into **10**; key to success was the blocking of the pyridine N‐atom with stoichiometric BF_3_⋅OEt_2_ to render the hydroboration of the terminal alkene possible.[^42^, ^43^] Though more pedestrian, this route proved robust, scalable and efficient (51 % yield over five steps, 6 g scale).

**Scheme 4:**
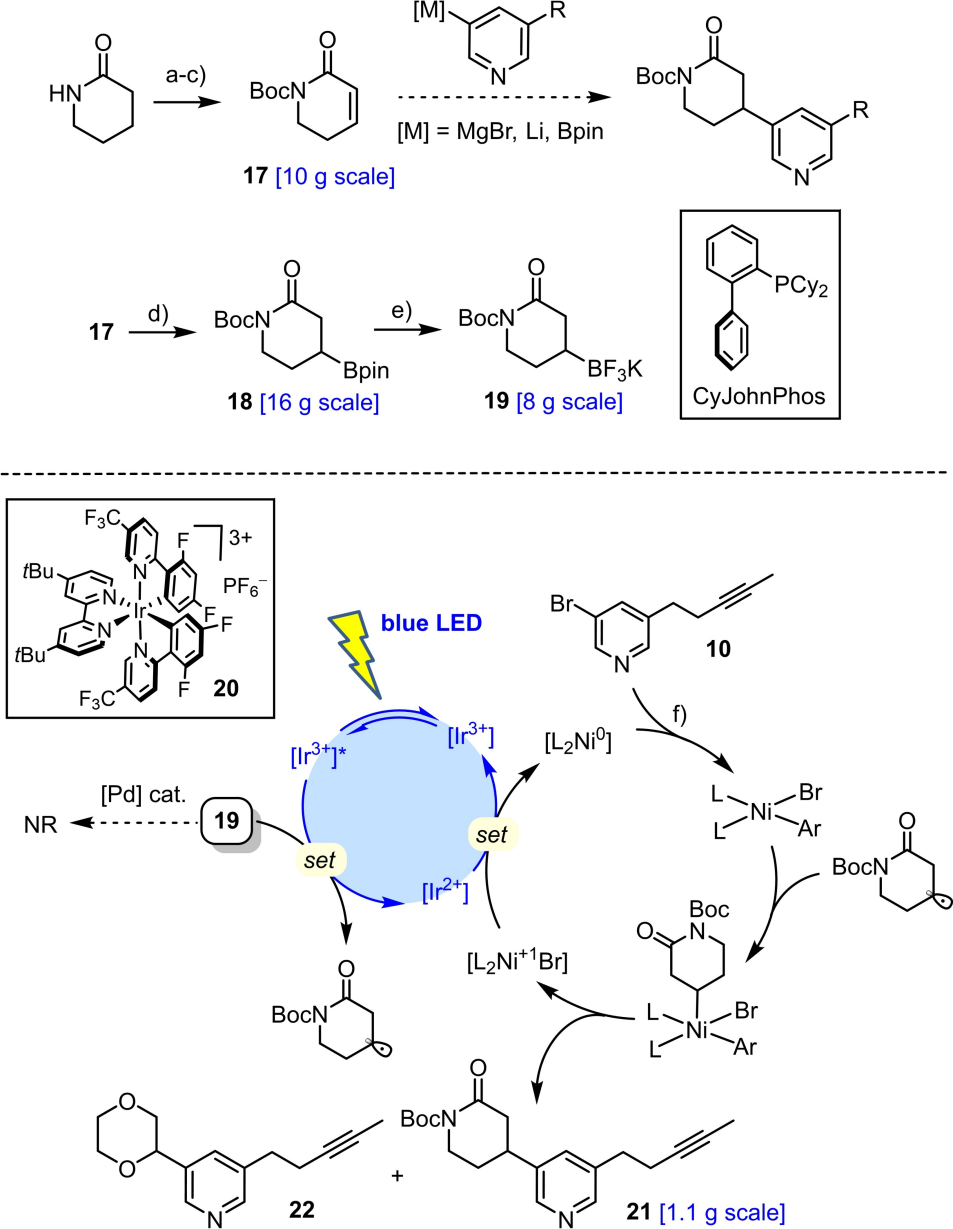
a) *n*BuLi, Boc_2_O, THF, −78 °C→0 °C, 98 %; b) LiHMDS, PhSeCl, THF, −78 °C→0 °C, 55–73 %; c) H_2_O_2_, THF, 0 °C, 96 %; d) B_2_pin_2_, CuCl (1.5 mol %), CyJohnPhos (1.5 mol %), *t*BuONa, EtOH, 0 °C, 98 %; e) KHF_2_, H_2_O, MeOH, 62 %; f) **10**, **19** (1.4 equiv), NiCl_2_⋅dme (3 mol %), 4,4′‐di‐*tert*‐butyl‐2,2′‐dipyridine (3 mol %), **20** (1 mol %), Cs_2_CO_3_, 1,4‐dioxane, blue LED (475 nm), 60 % (1.1 g scale; 74 % @ 630 mg scale).

Equally unrewarding were numerous attempts at conjugate addition of either pyridylboron or magnesium (lithium) reagents derived from **10** (or even simple pyridine model compounds) to the known Michael acceptor **17** ^[44]^ in the presence of rhodium[^45^, ^46^] or copper catalysts,^[47]^ respectively; although the lactam is activated by the additional N‐Boc substituent, the desired products were formed in trace amounts at best (Scheme 4). Polarity inversion was therefore deemed the way forward. To this end, **17** was subjected to a copper catalyzed conjugate borylation to give multigram quantities of boronate ester **18**.^[48]^ Whereas an attempted regular alkyl‐Suzuki coupling of this compound or of the derived trifluoroborate salt **19** with bromopyridine **10** also met with failure,^[49]^ the more recent cross coupling chemistry based on photoredox nickel/iridium dual catalysis provided a convenient solution.[^50^, ^51^] Thus, irradiation of a mixture comprising **10** and **19**, the commercial iridium photocatalyst **20** (1 mol %), NiCl_2_⋅dme (3 mol %), di‐*tert*‐butyldipyridine (3 mol %), and Cs_2_CO_3_ (1.5 equiv) in 1,4‐dioxane with a blue LED (475 nm) afforded the desired adduct **21** in 60 % yield on a >1.1 g scale (74 %, 630 mg scale).[^52^, ^53^] The reaction proceeded slowly (72 h) but cleanly; only trace amounts (≤5 %) of by‐product **22** derived from 1,4‐dioxane were detected, which could be separated by flash chromatography.

**Scheme 5:**
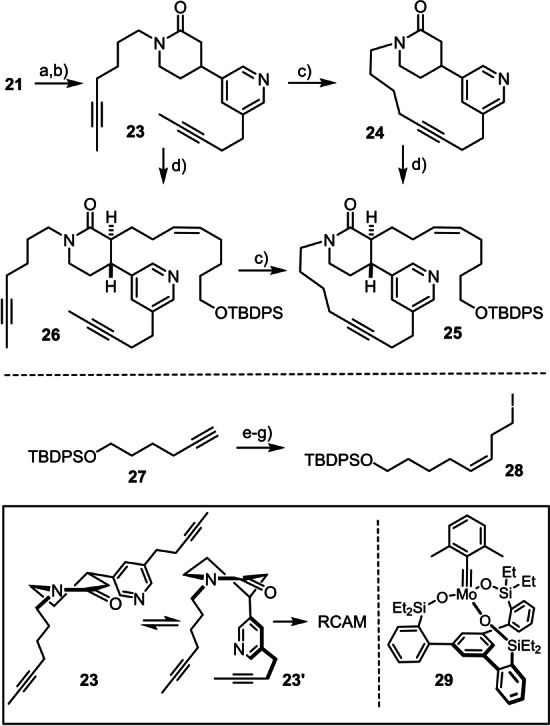
a) TFA, CH_2_Cl_2_, 0 °C, 64 %; b) NaH, 7‐iodo‐2‐heptyne, DMF, then *i*PrOH, 88 %; c) **29** (30 mol %), MS 5 Å, toluene, reflux, 91 % (**23**→**24**); 61 % (**26**→**25**); d) LDA, THF/DMPU, then **28**, −78 °C, 90 % (**24**→**25**); 85 % (**23**→**26**); e) *n*BuLi, BF_3_⋅OEt_2_, ethylene oxide, THF, −78 °C, 91 %; f) Ni(OAc)_2_⋅H_2_O, NaBH_4_, H_2_, ethylenediamine, EtOH, 84 %; g) I_2_, PPh_3_, imidazole, MeCN, Et_2_O, 93 %; DMPU=N,N′‐dimethyl‐propyleneurea; LDA=lithium diisopropylamide; MS=molecular sieves; TFA=trifluoroacetic acid.

Compound **21** was then elaborated into diyne **23** in readiness for the first macrocyclization event (Scheme 5).^[54]^ In line with our expectations, exposure to the latest‐generation canopy catalyst **29**[^34^, ^55^] in refluxing toluene readily gave the 15‐membered macrocycle **24** in 91 % yield despite the confined “*meta/para*‐bridging” array. In assessing this result, one has to consider that any unhindered pyridine, as the one found in substrate and product in the present case, binds to the high‐valent and hence inherently Lewis‐acidic Mo(+6) center of the operative molybdenum alkylidyne unit;^[55]^ by virtue of the silanolate ancillary ligand sphere, however, this coordination is reversible and does not prevent the catalyst from activating the triple bonds. Although a fairly high loading was chosen in order not to run the risk of losing valuable material at this stage, the remarkable chemoselectivity manifested in this transformation is best appreciated if one takes into account that even the proficiently tolerant Grubbs carbenes for olefin metathesis usually fail when applied to substrates comprising unhindered pyridine rings.^[56]^

**Figure 2:**
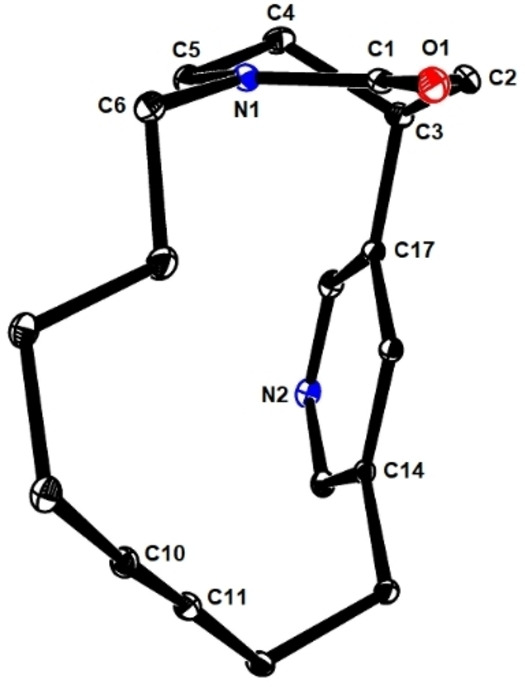
Structure of cycloalkyne **24** in the solid state.

The conformation that diyne **23** has to adopt for macrocyclization to proceed is preserved in the half‐chair structure of the piperidinone ring of product **24** in the solid state (Figure 2).^[57]^ For the planarity and rigidity of the lactam linkage, it takes little structural reorganization to interconvert the two conceivable half‐chair forms of the cyclization precursor **23**, such that the productive conformer is well populated in solution, in which the heteroarene is pseudo‐axial and the appended alkyne hence close enough in space to the chain branching off the N‐atom (Scheme 5). This advantage is likely lost upon reduction of the lactam: since the resulting piperidine is unconstrained at this stage, it adopts an ordinary chair conformation, which renders the axial disposition of the pyridine unit unfavorable and hence almost certainly impedes or even prevents macrocyclization.^[58]^

For this reason, it was clear that RCAM had to precede reduction of the piperidinone, whereas the best timing for the attachment of the handle needed for the projected second macrocyclization was less apparent. Because it could go before or after the RCAM reaction, both orders of events were explored. Specifically, deprotonation of **24** with LDA in rigorously degassed THF/DMPU at −78 °C followed by alkylation of the resulting enolate with the readily available iodide **28** furnished product **25** as a single diastereomer. Although formation of the *trans*‐isomer was expected, the recorded spectroscopic data were inconclusive, likely as a consequence of the conformational peculiarity of the now trisubstitued piperidinone ring inscribed into an unsaturated macrocyclic shackle (for details, see the Supporting Information). No such ambiguity was noticed when the enolate derived from **23** was alkylated with **28**; the *trans*‐disubstitution of the resulting product **26** is clearly manifested in the coupling constant for the protons at the branching points (^3^ *J*_H,H_=10 Hz). Treatment of this functionalized diyne with the “canopy catalyst” **29** ^[55]^ also entailed smooth macrocyclization; the resulting product **25** was identical in all regards with the compound formed by alkylation of **24**. However, its separation from the silanolate residues derived from the catalyst during work‐up proved somewhat more difficult than in the case of **24**, resulting in an isolated yield of only 61 %.

**Scheme 6:**
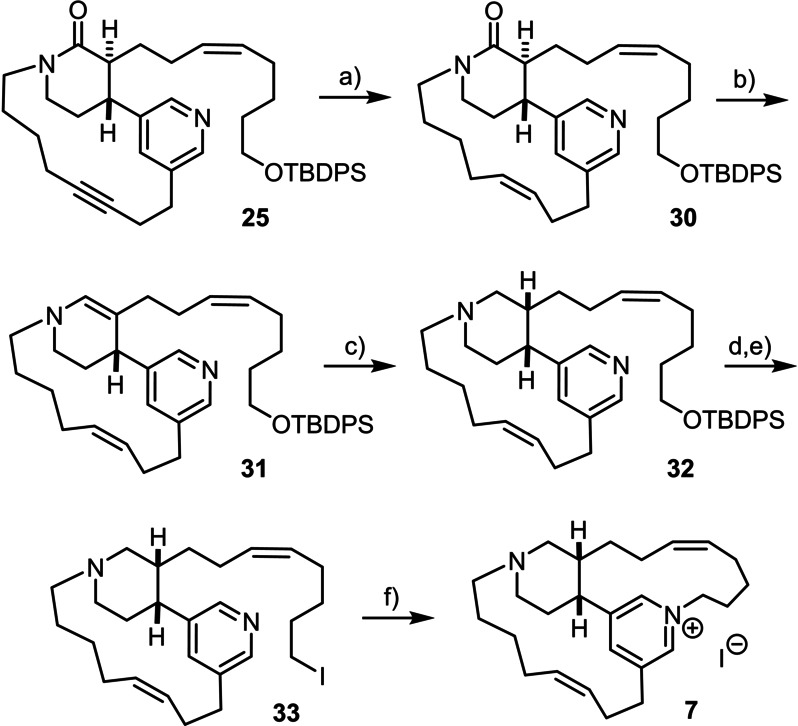
a) Ni(OAc)_2_⋅H_2_O, NaBH_4_, H_2_, EtOH, ethylenediamine, 94 %; b) [IrCl(CO)(PPh_3_)_2_] (12 mol %), tetramethyldisiloxane, toluene; c) NaBH_3_CN, HOAc, 63 % (over both steps); d) TBAF, THF, 87 %; e) I_2_, PPh_3_, imidazole, MeCN, Et_2_O, 79 %; f) MeCN (2 mM), reflux, 49 %.

With the two routes converging at product **25**, which was selectively hydrogenated over excess nickel boride to furnish the corresponding *Z*‐alkene **30**,[^59^, ^60^] we faced the difficult task of achieving full reduction of the amide, ideally in a catalytic fashion, without affecting the pyridine; classical reducing agents such as LiAlH_4_ or LiBHEt_3_ do not meet this requirement.^[61]^ At the same time, the stereochemcial and positional integrity of the peripheral double bonds must be ensured, a condition which ruled the use of Dibal‐H or B_2_H_6_ out (Scheme 6). The required chemoselectivity profile is unusual to the extent that it seems to have little precedent, if any. Only a highly oxophilic reagent with pronounced affinity to an amide carbonyl might have a chance to meet these stringent criteria.^[62]^ Inspiration was drawn from the work of Nagashima and co‐workers, who showed that the combination of [IrCl(CO)(PPh_3_)_2_] (Vaska's complex) as catalyst with tetramethyldisiloxane (TMDS)^[63]^ effects the selective reduction of amides over esters, ketones, enones and other potentially reducible sites.[^64^, ^65^] The reaction (and variants thereof) has already stood the test of target‐oriented synthesis,^[66]^ although we are unaware of any application to a substrate containing a pyridine or related heterocycle. Gratifyingly, this catalyst system allowed compound **30** to be transformed into the corresponding enamine **31** as the expected primary product;^[64]^ the reaction was slow but clean, without any sign of competing attack at the pyridine ring. The crude material could therefore be directly subjected to reduction to the desired tertiary amine via the corresponding iminium intermediate re‐generated in situ upon protonation. Although the pyridine might interfere again at this stage, the combination NaBH_3_CN/HOAc in MeOH proved effective, furnishing product **32** in 63 % yield over both steps as the only detectable product. This step proceeded stereoselectively, in that protonation occurred exclusively from the less hindered side to give the *cis*‐configured piperidine.^[67]^ This stereochemical outcome was confirmed by detailed analysis of the spectral data of **32** and, ultimately, by conversion of this product into *epi*‐tetradehydrohalicyclamine B (**7**).

With **32** in hand, the stage was set for the final macrocyclization via pyridine‐N‐alkylation. To this end, the TBDPS‐ether was cleaved and the resulting primary alcohol converted into iodide **33** under standard conditions.^[68]^ Stirring of a dilute solution (2 mM) of this compound in MeCN at reflux temperature furnished **7** in 49 % yield; this outcome is notable if one considers the strain and rigidity of the incipient 13‐membered ring^[24]^ and the fact that the substrate comprises an *a priori* more nucleophilic tertiary amine in vicinity to the pyridine: it is the even higher strain and less favorable kinetics of closure of the 11‐membered ring, which would be formed on N‐alkylation of the piperidine, that prevent this potentially competing site from interfering. Despite the ionic nature of and the presence of a tertiary amine in the resulting product **7**, this compound could be isolated as the corresponding iodide salt by conventional flash chromatography on silica, without need to resort to reverse phase HPLC.^[54]^

Regardless of the uncertainty concerning the escorting anion in the natural product derived for the marine sponge,^[69]^ the spectral data of synthetic **7** matched the literature very well.^[22]^ Most characteristic is the upfield shift of one of the protons each of the C8 and C13 methylene groups, which the isolation team had observed only in the case of *epi*‐tetradehydrohalicyclamine B comprising the *cis*‐configured piperidine unit. This spectral fingerprint (see the Supporting Information) attests to the stiffness of the 13‐membered ring that places these protons in the anisotropy cone of the pyridine and the C10–C11 alkene;^[24]^ at the same time, it confirms that the two‐step reduction of lactam **30** via enamine **31** exclusively provides the *cis*‐configured piperidine **32**. The price to pay for this selective course is the need to find an alternative route to the isomeric natural product tetradehydrohalicyclamine B (**6**), which will be subject to further investigations in this laboratory.^[67]^

## Conclusion

The first conquest of a tetracyclic pyridinium alkaloid of prominent biosynthetic pedigree bears witness to significant recent advances in chemoselectivity management beyond the scope of classical organic synthesis. If one considers that pyridine derivatives inhibit countless acidic reagents and metal catalysts, it is truly remarkable that nickel/iridium photoredox dual catalysis triumphs over canonical cross coupling as well as 1,4‐addition reactions when it comes to the elaboration of such building blocks. Equally noteworthy is the fact that the latest generation of alkyne metathesis catalysts is not obstructed by their presence either; this observation is particularly striking since the operative molybdenum alkylidyne unit comprises the early transition metal in the highest possible oxidation state. Finally, the current total synthesis hinged upon the stunning selectivity profile of a catalyst system comprised of Vaska's complex/TMDS that allows for direct catalytic reduction of a lactam without touching a flanking pyridine. Taken together, these virtues of contemporary catalysis open access to valuable material that cannot easily be obtained from the producing marine organism; relevant bio‐data will be reported in due course.

## Conflict of interest

The authors declare no conflict of interest.

1

## Supporting information

As a service to our authors and readers, this journal provides supporting information supplied by the authors. Such materials are peer reviewed and may be re‐organized for online delivery, but are not copy‐edited or typeset. Technical support issues arising from supporting information (other than missing files) should be addressed to the authors.

Supporting Information

## Data Availability

The data that support the findings of this study are available in the Supporting Information of this article.
