# Supplementary material for: Total Synthesis of the Tetracyclic Pyridinium Alkaloid epi‐Tetradehydrohalicyclamine B
Source: Angew Chem Int Ed Engl. 2022 Sep 2;61(41):e202209651. doi: 10.1002/anie.202209651 (PMC9826155; doi:10.1002/anie.202209651)

Angewandte Chemie
Eine Zeitschrift der Gesellschaft Deutscher Chemiker

Supporting Information

Total Synthesis of the Tetracyclic Pyridinium Alkaloid *epi*-Tetradehydrohalicyclamine B

*A. G. Dalling, G. Späth, A. Fürstner**

Supporting Crystallographic Information

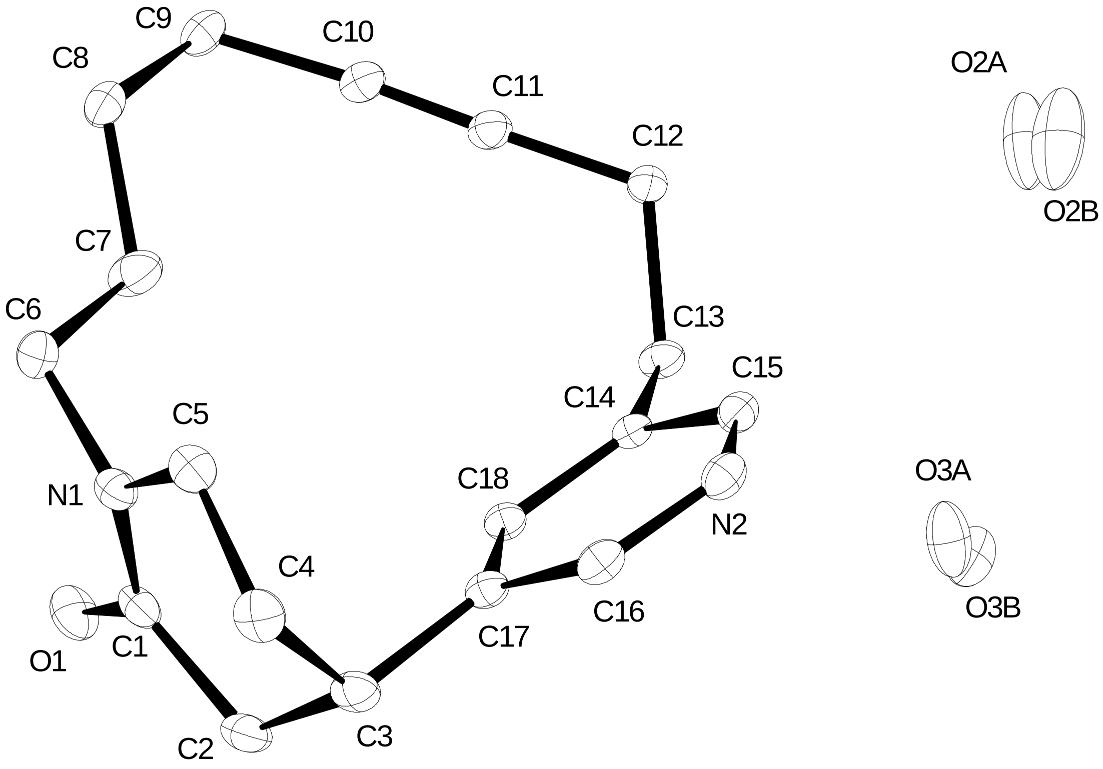

Figure S1. Structure of cycloalkyne **24** in the solid state; arbitrary numbering scheme; the unit cell also contains two disordered (50:50 occupancy) water molecules.

X-ray Crystal Structure Analysis of Compound 24: $C_{18}H_{26}N_2O_3$, M_r = 318.41 g mol^{-1}, colorless prism, crystal size 0.113 x 0.100 x 0.061 mm^3, monoclinic, space group $P2_1$/c [14], a = 8.9450(6) Å, b = 8.0539(5) Å, c = 23.1040(15) Å, β = 90.993(2)°, V = 1664.21(19) Å^3, T = 100(2) K, Z = 4, D_{calc} = 1.271 g·cm^3, λ = 0.71073 Å, $\mu(Mo\text{-}K_\alpha)$ = 0.087 mm^{-1}, Gaussian absorption correction (T_{min} = 1.00, T_{max} = 0.99), Bruker-AXS Kappa Mach3 with APEX-II detector and IµS microfocus source, 1.763 < θ < 35.215°, 118734 measured reflections, 7394 independent reflections, 6547 reflections with $I > 2\sigma(I)$, R_{int} = 0.0285. The structure was solved by *SHELXT* and refined by full-matrix least-squares (*SHELXL*) against F^2 to R_1 = 0.041 [$I > 2\sigma(I)$], wR_2 = 0.116, S = 1,045, 238 parameters, largest diff. peak and hole = 0.7 (0.68 Å from C17) and –0.3 (0.75 Å from O2B) e · Å^{-3}.

The complete dataset is available under the CCDC number **CCDC-2180239.**

General

Unless otherwise stated, all reactions were performed in oven-dried (80 °C) or flame-dried glassware in anhydrous solvents under argon, applying standard Schlenk techniques. Dry argon (>99.5%) was purchased from Air Liquide.

The following solvents were purified by distillation over the indicated drying agents and transferred under argon: tetrahydrofuran and diethyl ether (Mg/anthracene), dichloromethane (CaH_2), hexanes and toluene (Na/K), methanol (Mg, stored over molecular sieves (3 Å)). Acetonitrile, dimethyl sulfoxide, dimethylformamide, pyridine and triethylamine were dried using an adsorption (molecular sieves) solvent purification system. During work-up, solvents were removed under reduced pressure below 40 °C using a rotary evaporator.

Thin layer chromatography (TLC) was performed on Macherey-Nagel precoated plates (POLYGRAM® SIL/UV254); the compounds were detected by UV light (254 nm) and/or heating of the plate with a heat gun after treatment with a potassium permanganate stain solution. Flash chromatography was performed with VWR silica gel 60 (40 – 63 µm), unless stated otherwise.

NMR spectra were recorded on Bruker AV 400, AV 500 or AVIII 600 spectrometers in the solvents indicated. The solvent signals were used as references; chemical shifts were converted to the TMS scale and reported as follows: chemical shift in ppm (multiplicity, coupling constant *J* in Hz, number of protons). Multiplets are designated by the following abbreviations: s for singlet, d for doublet, t for triplet, q for quartet, quint for quintet, m for complex pattern (multiplet); the abbreviation br indicates a broad signal. ^{13}C NMR spectra were recorded in {^{1}H}-decoupled mode. Melting points were determined using a Büchi B-540 apparatus. IR spectra were recorded on a Bruker Alpha Platinum ATR spectrometer at room temperature. Mass spectra were recorded using the following instruments: MS (EI): Finnigan MAT 8200 (70 eV), ESI-MS: Bruker ESQ3000, accurate mass determinations: Bruker APEX III FT-MS (7 T magnet) or Finnigan MAT 95. GC-MS samples were processed on a Shimadzu GCMS-QP2010 Ultrainstrument.

Experimental Procedures

3-Allyl-5-bromopyridine (15). *i*-PrMgCl•LiCl (1.30 M in THF, 88 mL, 114 mmol) was transferred *via*

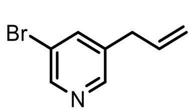

cannula to a 500-mL jacketed flask under argon, and the solution was cooled to −15 °C. 3,5-Dibromopyridine (25.8 g, 109 mmol) was added portionwise over 2 min, followed by THF (6 mL) to rinse the sides of the vessel. The resulting orange-brown suspension was stirred at −15 °C for 5 min, after which the temperature was increased to −10 °C. The suspension was stirred at this temperature for 40 min, during which the 3,5-dibromopyridine had almost fully dissolved. THF (10 mL) was added to aid dissolution and the reaction was stirred for a further 5 min. Allyl bromide (10.4 mL, 121 mmol) was added dropwise *via* syringe over 2 min, followed by a solution of CuCN•2LiCl (1 M in THF, 1 mL, 1 mmol). The resulting deep-red and homogeneous solution was warmed to 0 °C, and after approximately 7 min, the reaction had turned to a thick beige suspension. This suspension was stirred at 0 °C for 45 min before the reaction was quenched with sat. aq. NH_4Cl (50 mL) and water (50 mL). The suspension was warmed to room temperature and diluted with EtOAc (100 mL). Stirring was continued for 20 min until two layers had formed. The layers were separated and the aqueous phase was extracted with EtOAc (2 × 100 mL). The combined organic layers were washed with brine (100 mL), dried over anhydrous Na_2SO_4, filtered, and concentrated under reduced pressure to afford a red oil. The oil was loaded onto a short pad of silica gel, which was flushed with *tert*-butyl methyl ether (400 mL) and EtOAc (400 mL) to afford the title compound (21.2 g, 98% yield) as an orange oil. ^{1}H NMR (400 MHz, CD_2Cl_2): δ 8.51 (br s, 1H), 8.36 (br s, 1H), 7.68 (t, *J* = 2.1 Hz, 1H), 5.94 (ddt, *J* = 16.9, 10.1, 6.7 Hz, 1H), 5.22 – 5.05 (m, 2H), 3.38 (dq, *J* = 6.7, 1.1 Hz, 2H); ^{13}C NMR (101 MHz, CD_2Cl_2): δ 149.0, 148.7, 138.9, 137.8, 135.9, 120.9, 117.5, 37.1; IR (film) ν / cm^{-1}: 3036, 1639, 1579, 1555, 1420, 1095, 1021, 918, 668; MS (EI): *m/z* (%): 117 (100), 198 (49); HRMS (ESI): calcd. for C_8H_9NBr [M+H$^+$]: 197.9914; found: 197.9913.

The spectroscopic properties of this compound were consistent with the data available in the literature.[1]

3-(5-Bromopyridin-3-yl)propan-1-ol (16). BF_3•OEt_2 (13.6 mL, 111 mmol) was added

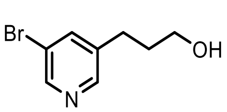

dropwise *via* syringe over 5 min to a solution of alkene **15** (20.5 g, 103 mmol) in THF (400 mL) at 0 °C in a three-necked 1-L jacketed flask equipped with addition funnel and thermometer. A freshly prepared solution of 9-borabicyclo[3.3.1]nonane (0.50 M in THF, 16.4 g, 134 mmol) was added dropwise *via* the addition funnel over 40 min at such a rate that the internal temperature remained at approximately 2 °C. After complete addition, the funnel was rinsed with THF (10 mL) and the thermometer was replaced with a glass stopper. The orange, homogeneous solution was stirred at 0 °C for 1 h, warmed to 10 °C and stirred for another hour, before the mixture was warmed to 20 °C and stirred at this temperature for 18 h. The by then cloudy solution was treated with anhydrous *N*,*N*,*N*′,*N*′-tetramethylethylenediamine (8.52 mL, 56.8 mmol) and cooled to 0 °C. The stopper was replaced

with a thermometer before aq. NaOH (3 M, 100 mL) was added dropwise over 40 min, maintaining the rate of addition such that the internal temperature remained at approximately 2 °C. During the addition, the color of the solution changed from yellow to orange-red. aq. H_2O_2 (75 mL, 35% *w*/*w*) was added dropwise over 20 min, ensuring that the internal temperature did not rise above 15 °C. After complete addition, the mixture was warmed to room temperature and stirred for 1 h. During this time, the color changed from orange-red to beige. The mixture was transferred slowly into ice-cold sat. aq. $Na_2S_2O_3$ (400 mL) to quench the excess peroxide. The aqueous mixture was then extracted with EtOAc (2 × 200 mL), and the combined organic extracts were washed with sat. aq. $Na_2S_2O_3$ (3 × 200 mL; *the concentration of peroxides in the organic layer was checked using colorimetric test strips until the test was negative*) and brine (500 mL), dried over anhydrous Na_2SO_4, filtered, and concentrated under reduced pressure to afford an orange oil. Purification by flash chromatography (5% MeOH/CH_2Cl_2) on silica gel (*SiO_2 with 0.015 – 0.04 mm particle size was required to separate the product from 1,5-cyclooctanediol*) afforded the title compound (16.7 g, 75% yield) as a yellow oil. ^{1}H NMR (400 MHz, CD_2Cl_2): δ 8.47 (d, *J* = 2.0 Hz, 1H), 8.36 (d, *J* = 2.0 Hz, 1H), 7.69 (t, *J* = 2.0 Hz, 1H), 3.62 (td, *J* = 6.3, 5.1 Hz, 2H), 2.76 – 2.63 (m, 2H), 1.91 – 1.76 (m, 2H), 1.71 – 1.61 (m, 1H); ^{13}C NMR (101 MHz, CD_2Cl_2): δ 148.7, 148.6, 139.7, 138.8, 120.9, 61.8, 34.0, 29.2; IR (film) ν / cm^{-1}: 3314, 2931, 2863, 1421, 1054, 1020, 704; MS (EI): *m/z* (%): 117 (50), 198 (98), 215 (3); HRMS (ESI): calcd. for $C_8H_{10}NOBr$ [M$^+$]: 214.9941; found: 214.9940.

3-(5-Bromopyridin-3-yl)propanal (S1). Dess-Martin periodinane (28.6 g, 67.5 mmol) was added

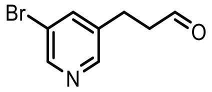

portionwise over 5 min to a stirring solution of alcohol **16** (9.72 g, 45.0 mmol) in anhydrous CH_2Cl_2 (400 mL) at 0 °C. The resulting suspension was stirred at 0 °C for 1.5 h before it was warmed to room temperature and stirred for another 1.5 h. At this point, full consumption of the starting material was reached (*as judged by ^{1}H NMR analysis of an aliquot*), and sat. aq. $NaHCO_3$ (150 mL) was added dropwise over 10 min, followed by sat. aq. $Na_2S_2O_4$ (150 mL). The biphasic mixture was stirred vigorously for 1 h before the layers were separated. The aqueous phase was extracted with CH_2Cl_2 (2 × 100 mL) and the combined organic portions were washed with sat. aq. $NaHCO_3$ (200 mL) and brine (200 mL), dried over Na_2SO_4, filtered, and concentrated under reduced pressure to afford the title compound (9.19 g, 95% yield) as an orange oil without need for purification. ^{1}H NMR (400 MHz, CD_2Cl_2): δ 9.79 (t, *J* = 1.0 Hz, 1H), 8.50 (d, *J* = 2.0 Hz, 1H), 8.39 (d, *J* = 2.0 Hz, 1H), 7.71 (m, 1H), 2.92 (ddt, *J* = 7.7, 7.0, 1.0 Hz, 2H), 2.81 (ddt, *J* = 7.7, 7.0, 1.0 Hz, 2H); ^{13}C NMR (101 MHz, CD_2Cl_2): δ 200.5, 149.1, 148.5, 138.8, 138.4, 120.9, 44.8, 25.1; IR (film) ν / cm^{-1}: 2828, 2727, 1719, 1421, 1097, 883, 698; MS (EI): *m/z* (%): 79 (65), 104 (70), 184 (100), 213 (6); HRMS (EI): calcd. for C_8H_9NOBr [M+H$^+$]: 213.9862; found: 213.9861.

3-Bromo-5-(but-3-yn-1-yl)pyridine (S2). A solution of dimethyl (1-diazo-2-oxopropyl)phosphonate

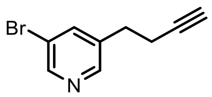

(9.92 g, 51.6 mmol) in anhydrous MeOH (20 mL) was added dropwise over 10 min *via* an addition funnel to a stirred suspension of aldehyde **S1** (8.50 g, 39.7 mmol) and K_2CO_3 (11.0 g, 79.4 mmol) in anhydrous MeOH (200 mL) at room temperature. The resulting yellow suspension gradually became green, and stirring was continued at room temperature for 1.5 h. sat. aq. $NaHCO_3$ (100 mL) and water (20 mL) were added to quench the reaction, followed by EtOAc (200 mL). The layers were separated and the aqueous phase was extracted with EtOAc (2 × 100 mL). The combined organic portions were washed with water (100 mL) and brine (100 mL), dried over anhydrous Na_2SO_4, filtered, and concentrated under reduced pressure. Purification of the residue by flash chromatography (25% EtOAc/hexanes) on silica afforded the title compound (7.50 g, 90% yield) as a pale yellow oil. ^{1}H NMR (400 MHz, CD_2Cl_2): δ 8.53 (d, J = 2.3 Hz, 1H), 8.41 (d, J = 1.9 Hz, 1H), 7.75 (m, 1H), 2.81 (t, J = 7.2 Hz, 2H), 2.51 (td, J = 7.2, 2.6 Hz, 2H), 2.05 (t, J = 2.6 Hz, 1H); ^{13}C NMR (101 MHz, CD_2Cl_2): δ 149.3, 148.7, 138.9, 137.9, 120.8, 82.9, 70.1, 31.7, 20.3; IR (film) ν / cm^{-1}: 3298, 1581, 1556, 1421, 1096, 1022, 704; MS (EI): *m/z* (%): 91 (43), 130 (100), 170 (28), 210 (34); HRMS (EI): calcd. for C_9H_9NBr [M+H$^+$]: 209.9913; found: 209.9914.

3-Bromo-5-(pent-3-yn-1-yl)pyridine (10). A freshly prepared solution of LiHMDS (1 M in THF, 43 mL,

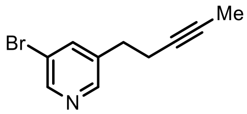

43 mmol) was added dropwise over 10 min to a solution of alkyne **S2** (7.00 g, 33.3 mmol) in THF (300 mL) at −78 °C. The resulting solution was stirred for 1 h at −78 °C, during which time the mixture became cloudy. A solution of iodomethane (10.3 mL, 166 mmol) in THF (20 mL) was added *via* syringe over 5 min before the mixture was warmed to 0 °C, which led to the formation of a homogeneous, pale yellow solution. After 40 min, the reaction was quenched with water (100 mL), the mixture was diluted with *tert*-butyl methyl ether (200 mL), and the layers were separated. The aqueous portion was extracted with *tert*-butyl methyl ether (2 × 100 mL), and the combined organic phases were washed with water (200 mL) and brine (200 mL), dried over anhydrous Na_2SO_4, filtered, and concentrated under reduced pressure. Purification of the residue by flash chromatography (10% EtOAc/hexanes) on silica afforded the title compound (6.06 g, 81% yield) as a colorless crystalline solid. m.p. 52 – 54 °C; ^{1}H NMR (400 MHz, CD_2Cl_2): δ 8.51 (d, J = 2.1 Hz, 1H), 8.39 (d, J = 2.1 Hz, 1H), 7.74 (t, J = 2.1 Hz, 1H), 2.76 (t, J = 7.1 Hz, 2H), 2.43 (tq, J = 7.1, 2.5 Hz, 2H), 1.74 (t, J = 2.5 Hz, 3H); ^{13}C NMR (101 MHz, CD_2Cl_2): δ 234.2, 149.0, 148.7, 138.9, 138.5, 120.7, 77.6, 77.5, 32.4, 20.7, 3.4; IR (film) ν / cm^{-1}: 2948, 2915, 1555, 1421, 1096, 1019, 863; MS (EI): *m/z* (%): 171 (100), 210 (25), 222 (52), 224 (44); HRMS (ESI): calcd. for $C_{10}H_{11}NBr$ [M+H$^+$]: 224.0070; found: 224.0070.

***tert*-Butyl 2-oxopiperidine-1-carboxylate (S3).** In a 1-L three-necked jacketed flask, *n*-BuLi (1.6 M in hexanes, 68 mL, 109 mmol) was added dropwise over 30 min *via* addition funnel to a solution of 2-piperidone (11.2 g, 113 mmol) in THF (500 mL) at −78 °C. After complete addition, the addition funnel was rinsed with anhydrous THF (20 mL) and the pale yellow, homogeneous solution was stirred for further 30 min at −78 °C. A solution of di-*tert*-butyl dicarbonate (28.3 g, 130 mmol) in THF (100 mL) was added *via* the addition funnel over 20 min. The mixture was stirred for further 45 min before being warmed to 0 °C. The reaction was qenched by dropwise addition of water (80 mL) *via* the addition funnel, EtOAc (200 mL) was introduced and the layers were separated. The aqueous phase was extracted with EtOAc (2 × 200 mL), and the combined organic layers were washed with water (300 mL) and brine (200 mL), dried over anhydrous Na_2SO_4, filtered and concentrated under reduced pressure. Purification of the residue by flash chromatography (15 – 30% EtOAc/hexanes) on silica afforded the title compound (22.1 g, 98% yield) as a colorless oil. ^{1}H NMR (400 MHz, $CDCl_3$): δ 3.65 (dddd, *J* = 6.3, 3.7, 2.9, 1.5 Hz, 2H), 2.55 – 2.43 (m, 2H), 1.89 – 1.76 (m, 4H), 1.52 (s, 9H); ^{13}C NMR (101 MHz, $CDCl_3$): δ 171.5, 152.9, 83.0, 46.4, 35.0, 28.2, 22.9, 20.7; IR (film) ν / cm^{-1}: 2956, 1769, 1705, 1287, 1246, 1135, 1058; MS (EI): *m/z* (%): 41 (70), 99 (100), 144 (69); HRMS (ESI): calcd. for $C_{10}H_{17}NO_3Na$ [M+Na$^+$]: 222.1101; found: 222.1103.

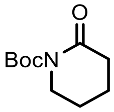

The spectroscopic properties of this compound were consistent with the data available in the literature.[2]

***tert*-Butyl 2-oxo-3-(phenylselanyl)piperidine-1-carboxylate (S4).** A freshly prepared solution of LiHMDS (1 M in THF, 120 mL, 120 mmol) was added dropwise over 20 min *via* an addition funnel to a stirring solution of lactam **S3** (20.0 g, 100 mmol) in THF (400 mL) at −78 °C in a 1-L three-necked jacketed flask. The resulting mixture was stirred for 1 h at −78 °C before a solution of phenylselenyl chloride (21.1 g, 110 mmol) in anhydrous THF (50 mL) was introduced over 10 min *via* the addition funnel (*a moderate rate of addition is important to minimize the formation of the diselenated side-product; lower yields were obtained with a slower rate of addition (i.e. >20 min))*. The resulting orange solution was maintained at −78 °C for 30 min before being analyzed by TLC. Upon consumption of the starting material, the mixture was warmed to 0 °C and the reaction was quenched by the dropwise addition of water (100 mL). EtOAc (200 mL) was introduced and the resulting layers were separated. The aqueous phase was extracted with EtOAc (2 × 200 mL) and the combined organic extracts were washed with water (300 mL) and brine (300 mL), dried over anhydrous Na_2SO_4, filtered and concentrated under reduced pressure to afford an orange oil. Purification by flash chromatography (10 – 25% EtOAc/hexanes) on silica afforded the title compound (19.5 g, 55% yield) as an orange oil. ^{1}H NMR (400 MHz, $CDCl_3$): δ 7.72 – 7.62 (m, 2H), 7.37 – 7.26 (m, 3H), 3.97 (td, *J* = 5.7, 0.9 Hz, 1H), 3.75 – 3.56 (m, 2H), 2.27 – 2.16 (m, 1H), 2.13 – 1.93 (m, 2H), 1.82 – 1.71 (m, 1H), 1.53 (s, 9H); ^{13}C NMR (101 MHz, $CDCl_3$): δ 170.6, 153.2, 135.9, 129.2, 128.7, 128.5, 83.1, 46.2, 45.1, 28.9, 28.2,

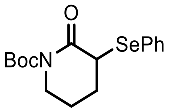

21.3; IR (film) ν / cm^{-1}: 2978, 1706, 1284, 1246, 1138, 738; MS (EI): *m/z* (%): 157 (25), 174 (100), 255 (28); HRMS (ESI): calcd. for $C_{16}H_{21}NO_3SeNa$ [M+Na$^+$]: 378.0576; found: 378.05787.

The spectroscopic properties of this compound were consistent with the data available in the literature.[3]

***tert*-Butyl 6-oxo-3,6-dihydropyridine-1(2*H*)-carboxylate (17).** A solution of aq. H_2O_2 (35% *w*/*w*, 13.4 mL, 138 mmol) was added dropwise *via* addition funnel over 10 min to a stirring solution of selenide **S4** (19.5 g, 55.0 mmol) in THF (300 mL) at 0 ºC. The resulting yellow, homogeneous solution was stirred for 15 min at 0 °C before being warmed to room temperature and stirred for additional 30 min at this temperature. Upon consumption of the starting material, the mixture was diluted with CH_2Cl_2 (200 mL) and washed with a sat. aq. $NaHCO_3$ (200 mL). The aqueous layer was extracted with CH_2Cl_2 (2 × 200 mL) and the combined organic phases were washed with sat. aq. $Na_2S_2O_3$ (200 mL), dried over anhydrous Na_2SO_4, filtered, and concentrated under reduced pressure to afford a yellow oil. Purification by flash chromatography (25 – 40% EtOAc/hexanes) on silica afforded the title compound (10.4 g, 96% yield) as a pale yellow oil. ^{1}H NMR (400 MHz, $CDCl_3$): δ 6.82 – 6.71 (m, 1H), 5.96 (dt, *J* = 9.8, 1.9 Hz, 1H), 3.93 – 3.78 (t, *J* = 6.6 Hz, 2H), 2.46 – 2.34 (m, 2H), 1.54 (s, 9H); ^{13}C NMR (101 MHz, $CDCl_3$): δ 163.9, 152.8, 143.5, 126.5, 83.1, 43.7, 28.2, 24.9; IR (film) ν / cm^{-1}: 2980, 1762, 1698, 1307, 1289, 1229, 1154, 1130, 813; MS (ESI): *m/z*: 220 [M+Na$^+$]; HRMS (ESI): calcd. for $C_{10}H_{15}NO_3Na$ [M+Na$^+$]: 220.0944; found: 220.0948.

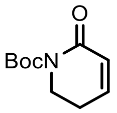

The spectroscopic properties of this compound were consistent with the data available in the literature.[3]

***tert*-Butyl 2-oxo-4-(4,4,5,5-tetramethyl-1,3,2-dioxaborolan-2-yl)piperidine-1-carboxylate (18).** A three-necked 500-mL jacketed flask under argon was charged with bis(pinacolato)diboron (14.1 g, 55.8 mmol), 2-(dicyclohexylphosphino)biphenyl (267 mg, 0.762 mmol), NaO*t*-Bu (1.46 g, 15.2 mmol) and CuCl (75.3 mg, 0.761 mmol). Anhydrous EtOH (50 mL) was added and the resulting brown suspension was cooled to 0 ºC. A solution of lactam **17** (10.0 g, 50.7 mmol) in anhydrous EtOH (120 mL) was then added *via* an addition funnel over 10 min. The resulting brown solution was stirred for 10 min at 0 °C, warmed to 5 ºC and stirred for a further 15 min before being filtered through Celite, rinsing with EtOAc (50 mL). The filtrate was concentrated under reduced pressure and the residue was purified by flash chromatography (10 – 30% EtOAc/hexanes) on silica (*flash chromatography should be conducted quickly as the boronic ester will decompose on extended exposure to acidic silica*) to afford the title compound (16.1 g, 98% yield) as a colorless oil. ^{1}H NMR (400 MHz, $CDCl_3$): δ 3.71 (ddd, *J* = 12.8, 5.7, 5.0 Hz, 1H), 3.57 (ddd, *J* = 12.8, 9.3, 5.0 Hz, 1H), 2.57 (m, 1H), 2.45 (dd, *J* = 17.3, 10.8 Hz, 1H), 1.95 (m, 1H), 1.74 (m, 1H), 1.52 (s, 9H), 1.44 (m, 1H), 1.23 (s, 12H); ^{13}C NMR (101 MHz, $CDCl_3$): δ 171.7, 153.0, 83.9, 82.9, 46.9, 36.2, 28.2, 24.9; *the ^{13}C signal corresponding to the boron-bound carbon atom was not observed due*

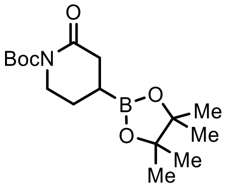

to quadrupolar relaxation; ^{11}B NMR (128 MHz, $CDCl_3$): δ 33.4; IR (film) ν / cm^{-1}: 2978, 1702, 1367, 1322, 1286, 1139, 853; MS (EI): *m/z* (%): 57 (100), 126 (29), 225 (32) HRMS (ESI): calcd. for $C_{16}H_{28}NO_5BNa$ [M+Na$^+$]: 348.1953; found: 348.1953.

Potassium (1-(*tert*-butoxycarbonyl)-2-oxopiperidin-4-yl)trifluoroborate (19). A solution of

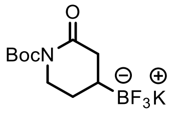

KHF_2 (13.4 g, 172 mmol) in water (38 mL) was added over 2 min to a stirred solution of boronate **18** (14.0 g, 43.0 mmol) in MeOH (200 mL) at 0 °C. After 5 min at 0 °C, the suspension was warmed to room temperature and stirred for 1 h. Volatile material was removed under reduced pressure (50 mbar, 45 – 50 °C), and remaining pinacol was removed azeotropically by repeatedly dissolving the white slurry in MeOH (50 mL) and water (50 mL) and removing the volatile material under reduced pressure (this process was repeated four times). The slurry was then transferred to a Büchner funnel, washed with *tert*-butyl methyl ether (2 × 100 mL) and dried under vacuum. At this point, ^{1}H NMR analysis indicated that all residual pinacol (CD_3CN: δ_H = 1.39, s) had been removed. The remaining free-flowing white solid, containing the desired product admixed with inorganic impurities, was purified by Soxhlet extraction (72 h) with refluxing acetone (1.5 L). The acetone was removed under reduced pressure to afford the title compound (8.20 g, 62% yield) as a white powder. m.p. 186 – 188 °C; ^{1}H NMR (400 MHz, CD_3CN): δ 3.64 (m, 1H), 3.46 (ddd, *J* = 12.2, 9.8, 4.8 Hz, 1H), 2.29 – 2.11 (m, 2H), 1.71 (m, 1H), 1.50 (m, 1H), 1.46 (s, 9H), 0.64 (m, 1H); ^{13}C NMR (101 MHz, CD_3CN): δ 176.2, 154.2, 82.3, 48.9, 38.8, 28.2, 26.2; *the ^{13}C signal corresponding to the boron-bound carbon atom was not observed due to quadrupolar relaxation*; ^{11}B NMR (128 MHz, CD_3CN): δ 4.50; ^{19}F (282 MHz, CD_3CN): δ −148.7; IR (film) ν / cm^{-1}: 2969, 1769, 1367, 1280, 1246, 1148, 1024, 851, 740; MS (ESI): *m/z*: 266 [M-K$^-$]; HRMS (ESI): calcd. for $C_{10}H_{16}NO_3F_3B$ [M$^-$]: 266.1181; found: 266.1183.

***tert*-Butyl 2-oxo-4-(5-(pent-3-yn-1-yl)pyridin-3-yl)piperidine-1-carboxylate (21).** A 100-mL flame-

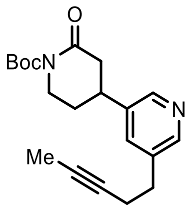

dried Schlenk tube under argon was charged with $NiCl_2$•dme (33.0 mg, 0.150 mmol) and 4,4′-di-*tert*-butyl-2,2′-dipyridyl (40.3 mg, 0.150 mmol). THF (10 mL) was added and the tube was heated gently with a heat gun until the nickel and ligand were fully solubilized, affording a pale green, homogeneous solution. After cooling to room temperature, the solvent was removed *in vacuo* to afford a pale green solid. The tube was refilled with argon, and charged with aryl bromide **10** (1.12 g, 5.00 mmol), trifluoroborate **19** (2.14 g, 7.00 mmol), (Ir[dF(CF_3)ppy]$_2$(dtbpy))PF_6 (**20**, 56.1 mg, 0.0500 mmol) and Cs_2CO_3 (2.44 g, 7.50 mmol). The tube was evacuated and refilled with argon three times, before 1,4-dioxane (dried over CaH_2, degassed by freeze-pump-thaw cycling, 100 mL) was added. The reaction vessel was vigorously stirred and irradiated with a 475 nm Hepatochem blue LED bulb (placed 10 – 15 cm away from the Schlenk tube) for 72 h. A fan was used to cool the reaction, and a bubbler was placed between the argon line and the Schlenk tube to avoid the build-up of pressure should the temperature increase (see pictures below). After 72 h, the suspension

was filtered through a short pad of Celite®, eluting with *tert*-butyl methyl ether, followed by filtration with fluted filter paper. The filtrate was concentrated under reduced pressure, and the residue was purified by flash chromatography (1.5 – 2% MeOH/CH_2Cl_2) on silica (SiO_2 with 0.015 – 0.04 mm particle size) to afford the title compound (1.03 g, 60% yield) as a yellow oil. ^{1}H NMR (400 MHz, CD_2Cl_2): δ 8.38 – 8.31 (m, 2H), 7.40 (t, J = 2.0 Hz, 1H), 3.88 (ddd, J = 12.9, 5.0, 3.9 Hz, 1H), 3.61 (ddd, J = 12.9, 11.2, 4.0 Hz, 1H), 3.13 (m, 1H), 2.82 – 2.73 (m, 3H), 2.58 (dd, J = 15.0, 11.5 Hz, 1H), 2.42 (tq, J = 7.3, 2.5 Hz, 2H), 2.18 (ddt, J = 11.8, 5.0, 2.0 Hz, 1H), 1.96 (dtd, J = 13.6, 11.3, 5.0 Hz, 1H), 1.74 (t, J = 2.5 Hz, 3H), 1.52 (s, 9H); ^{13}C NMR (101 MHz, CD_2Cl_2): δ 69.9, 152.9, 149.1, 146.8, 138.5, 136.6, 134.3, 83.1, 78.0, 77.3, 46.1, 42.2, 36.5, 32.7, 30.4, 28.1, 21.0, 3.5; IR (film) ν / cm^{-1}: 2979, 2930, 1767, 1710, 1288, 1233, 1145, 720; MS (ESI): *m/z*: 343 [M+H$^+$]; HRMS (ESI): calcd. for $C_{20}H_{26}N_2O_3Na$ [M+Na$^+$]: 365.1836; found: 365.1834.

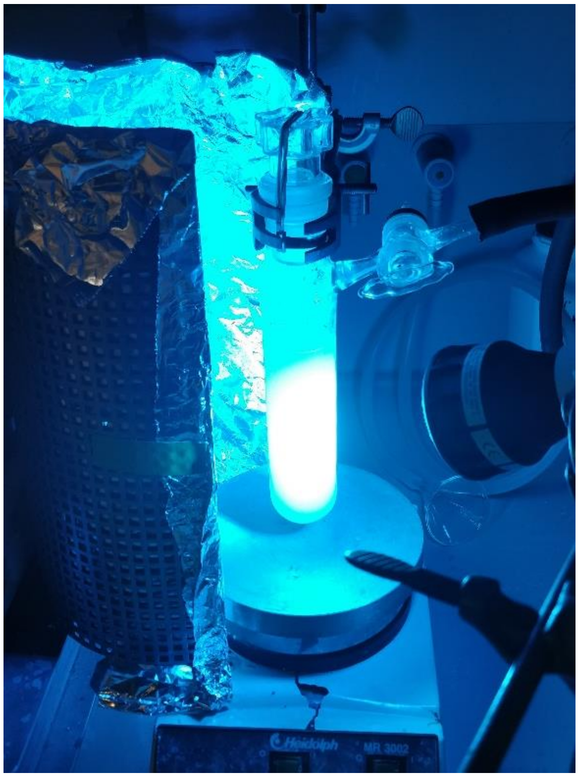

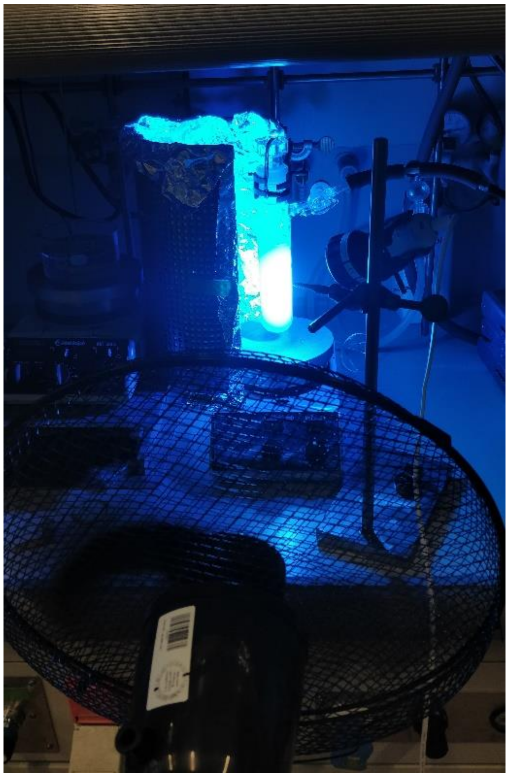

4-(5-(Pent-3-yn-1-yl)pyridin-3-yl)piperidin-2-one (S5). Trifluoroacetic acid (2.90 mL, 38.0 mmol) was added dropwise to a stirring solution of carbamate **21** (1.00 g, 2.92 mmol) in anhydrous CH_2Cl_2 (30 mL) at 0 °C. The resulting solution was stirred at 0 °C for 2 h before being concentrated under reduced pressure. sat. aq. Na_2CO_3 (15 mL) was added to the residue to adjust the pH to ≈ 11. CH_2Cl_2 (20 mL) was added and the layers were separated. The aqueous phase was extracted with CH_2Cl_2 (3 × 20 mL), and the combined organic portions were washed with water (50 mL) and brine (50 mL), dried over anhydrous Na_2SO_4, and concentrated under reduced pressure. Purification of the residue by flash chromatography (CH_2Cl_2/MeOH, 30:1 – 10:1) on silica afforded the title compound (454 mg, 64% yield) as a colorless powder. m.p. 145 – 146 °C; ^{1}H NMR (400 MHz, CD_2Cl_2): δ 8.35 (t, J = 2.3 Hz, 2H), 7.42 (t, J = 2.3 Hz, 1H), 6.02 (s, 1H), 3.40 (tt, J = 6.0, 2.2 Hz, 2H), 3.12 (tdd, J = 11.4, 5.3, 3.1 Hz, 1H), 2.77 (t, J = 7.2 Hz, 2H), 2.62 (ddd, J = 17.4, 5.3, 2.0 Hz, 1H), 2.48 – 2.37 (m, 3H), 2.06 (m, 1H), 1.93 (m, 1H), 1.75 (t, J = 2.5 Hz, 3H); ^{13}C NMR (101 MHz, CD_2Cl_2): δ 171.0, 148.9, 146.9, 138.9, 136.6, 134.4, 78.0, 77.2, 41.8, 38.9, 36.6, 32.7, 29.7, 21.0, 3.5; IR (film) ν / cm^{-1}: 3247, 2961, 2856, 1654, 1624, 1496, 1340, 1126; MS (ESI): *m/z*: 242 [M+H$^+$]; HRMS (ESI): calcd. for $C_{15}H_{19}N_2O$ [M+H$^+$]: 243.1492; found: 243.1494.

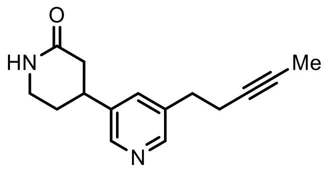

1-(Hept-5-yn-1-yl)-4-(5-(pent-3-yn-1-yl)pyridin-3-yl)piperidin-2-one (23). Solid piperidinone **S5** (272 mg, 1.12 mmol) was added to a suspension of NaH (158 mg, 6.58 mmol) in DMF (8.4 mL) at room temperature. The resulting brown suspension was stirred for 15 min before neat 7-iodohept-2-yne (350 mg, 1.58 mmol)[4] was introduced *via* syringe in one portion at room temperature. After stirring for 1.5 h, isopropanol (0.5 mL) was carefully added under cooling in an ice bath to quench the reaction (*gas formation!*). The mixture was warmed to room temperature and stirred for 20 min before *tert*-butyl methyl ether (30 mL) and water (20 mL) were added. The layers were separated and the organic layer was washed with water (3 × 15 mL) and brine (15 mL). The aqueous phase were extracted with *tert*-butyl methyl ether (3 × 15 mL) and the combined organic layers were dried over anhydrous Na_2SO_4. The drying agent was filtered off and the solvent removed under reduced pressure. Purification of the residue by flash chromatography (CH_2Cl_2/MeOH, 40:1 – 20:1) on silica afforded the title compound admixed with traces of remaining DMF, which were removed by aqueous extraction from *tert*-butyl methyl ether (25 mL, 3 × 10 mL H_2O), to furnish the pure title compound as a light amber gum (331 mg, 88% yield). ^{1}H NMR (400 MHz, CD_2Cl_2): δ 8.33 (dd, J = 5.4, 2.2 Hz, 2H), 7.40 (t, J = 2.2 Hz, 1H), 3.48 – 3.28 (m, 4H), 3.10 (tdd, J = 11.4, 5.3, 3.1 Hz, 1H), 2.76 (t, J = 7.2 Hz, 2H), 2.65 (ddd, J = 17.3, 5.4, 2.2 Hz, 1H), 2.50 – 2.35 (m, 3H), 2.20 – 2.07 (m, 3H), 1.97 (dddd, J = 13.2, 11.5, 10.0, 5.5 Hz, 1H), 1.79 – 1.72 (m, 6H), 1.69 – 1.61 (m, 2H), 1.51 – 1.43 (m, 2H); ^{13}C NMR (101 MHz, CD_2Cl_2): δ 168.4,

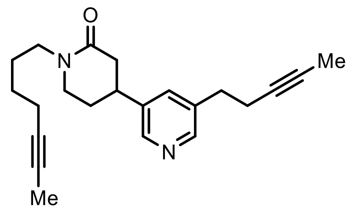

148.8, 146.9, 138.9, 136.5, 134.3, 79.1, 78.0, 77.2, 76.0, 47.1, 46.6, 39.6, 36.9, 32.8, 30.4, 26.8, 21.0, 18.8, 3.5 (*two signals*); IR (film) ν / cm^{-1}: 2918, 2859, 1634, 1494, 1433, 1344, 719; MS (EI): *m/z*: 336 [M^+]; HRMS (ESI): calcd. for $C_{22}H_{29}N_2O$ [$M+H^+$]: 337.2274; found: 337.2275.

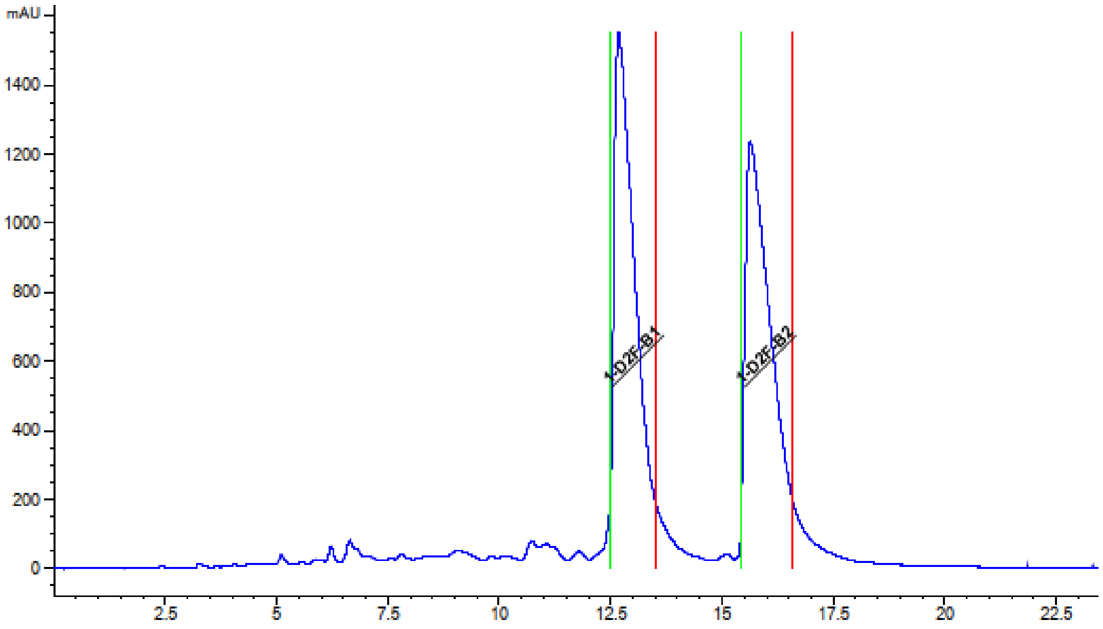

Chiral resolution by preparative HPLC was possible under the following conditions: Agilent 1260 Infinity pump, Chiralpak IC 5 μm column: 250 mm length, ∅ 20 mm; eluent: MeCN/H_2O (90:10 *v/v*); UV detection at 220 nm; the more rapidly eluting enantiomer is levorotatory

***tert*-Butyl(hex-5-yn-1-yloxy)diphenylsilane (27).** *tert*-Butyldiphenylchlorosilane (2.91 mL, 11.2 mmol)

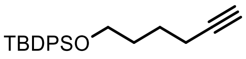

was added to a solution of imidazole (1.39 g, 20.4 mmol) and hex-5-yn-1-ol (1.00 g, 10.2 mmol) in CH_2Cl_2 (20 mL) at 0 °C. The resulting solution was warmed to room temperature and stirred for 16 h, before the reaction was quenched with water (50 mL). The layers were separated and the aqueous phase was extracted with CH_2Cl_2 (2 × 30 mL). The combined organic layers were washed with water (50 mL) and brine (50 mL), dried over anhydrous Na_2SO_4, filtered, and concentrated under reduced pressure. Purification of the residue by flash chromatography (1% *tert*-butyl methyl ether/hexanes) on silica afforded the title compound (2.43 g, 71% yield) as a colorless oil. ^1H NMR (400 MHz, $CDCl_3$): δ 7.69 – 7.63 (m, 4H), 7.44 – 7.34 (m, 6H), 3.74 – 3.59 (m, 2H), 2.19 (td, *J* = 6.8, 2.6 Hz, 2H), 1.93 (t, *J* = 2.7 Hz, 1H), 1.71 – 1.58 (m, 4H), 1.05 (s, 9H); ^{13}C NMR (101 MHz, $CDCl_3$): δ 135.7, 134.1, 129.7, 127.8, 84.7, 68.4, 63.5, 31.7, 27.0, 25.1, 19.4, 18.3; IR (film) ν / cm^{-1}: 3306, 2931, 2858, 1106, 700; MS (ESI): *m/z*: 337 [$M+H^+$]; HRMS (ESI): calcd. for $C_{22}H_{28}OSiNa$ [$M+Na^+$]: 359.1802; found: 359.1805.

The spectroscopic properties of this compound were consistent with the data available in the literature.[5]

8-((*tert*-Butyldiphenylsilyl)oxy)oct-3-yn-1-ol (S6). *n*-BuLi (1.60 M in hexanes, 4.83 mL, 7.73 mmol) was added dropwise over 5 min to a solution of alkyne **27** (2.00 g, 5.94 mmol) in THF (60 mL) at −78 °C. The resulting colorless solution was stirred at −78 °C for 30 min before BF_3•OEt_2 (1.03 mL, 8.32 mmol) was added *via* syringe in one portion, followed by ethylene oxide (2.5 – 3.3 M in THF, 3.17 mL, approx. 9.50 mmol). Stirring was continued at −78 °C for 1 h before the mixture was warmed to 0 °C and the reaction quenched with sat. aq. NH_4Cl (10 mL). Water (30 mL) and *tert*-butyl methyl ether (30 mL) were added and the layers were separated. The aqueous phase was extracted with *tert*-butyl methyl ether (2 × 30 mL) and the combined organic portions were washed with brine (100 mL), dried over anhydrous Na_2SO_4, filtered, and concentrated under reduced pressure. Purification of the residue by flash chromatography (15% EtOAc/hexanes) on silica afforded the title compound (2.06 g, 91% yield) as a colorless oil. ^{1}H NMR (400 MHz, $CDCl_3$): δ 7.69 – 7.64 (m, 4H), 7.44 – 7.35 (m, 6H), 3.72 – 3.61 (m, 4H), 2.42 (tt, *J* = 6.2, 2.4 Hz, 2H), 2.17 (tt, *J* = 6.8, 2.4 Hz, 2H), 1.69 – 1.51 (m, 4H), 1.05 (s, 9H); ^{13}C NMR (101 MHz, $CDCl_3$): δ 135.7, 134.2, 129.7, 127.8, 82.7, 76.6, 63.6, 61.5, 31.9, 27.0, 25.6, 23.3, 19.4, 18.7; IR (film) ν / cm^{-1}: 3379, 2931, 2858, 1106, 700; MS (ESI): *m/z*: 381 [M+H$^+$]; HRMS (ESI): calcd. for $C_{24}H_{32}O_2SiNa$ [M+Na$^+$]: 403.2064; found: 403.2066.

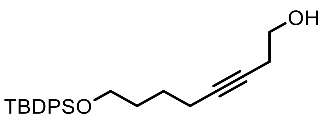

The spectroscopic properties of this compound were consistent with the data available in the literature.[6]

(*Z*)-8-((*tert*-Butyldiphenylsilyl)oxy)oct-3-en-1-ol (S7). $NaBH_4$ (258 mg, 6.83 mmol) was added portionwise to a solution of $Ni(OAc)_2$•$4H_2O$ (1.45 g, 5.80 mmol in EtOH (30 mL) at 0 °C. The resulting black suspension was vigorously stirred for 30 min at 0 °C before ethylenediamine (0.57 mL, 8.5 mmol) was added *via* syringe in one portion. After further 30 min, a solution of alkyne **S6** (1.30 g, 3.41 mmol) in EtOH (5 mL) was added *via* syringe. A balloon filled with H_2 was connected to the flask and hydrogen was bubbled through the black suspension for 2 min. The suspension was stirred for 2 h under the hydrogen atmosphere at 0 °C before it was filtered through a pad of Celite®, rinsing with EtOAc. The pink filtrate was concentrated under reduced pressure and the residue was purified by flash chromatography (20% EtOAc/hexanes) on silica to afford the title compound (1.10 g, 84% yield) as a colorless oil. ^{1}H NMR (400 MHz, $CDCl_3$): δ 7.70 – 7.63 (m, 4H), 7.44 – 7.35 (m, 6H), 5.54 (m, 1H), 5.37 (dtt, *J* = 10.6, 7.4, 1.6 Hz, 1H), 3.72 – 3.57 (m, 4H), 2.30 (tddd, *J* = 6.5, 5.9, 1.5, 0.7 Hz, 2H), 2.06 (tddd, *J* = 7.4, 6.8, 6.2, 1.7 Hz, 2H), 1.62 – 1.52 (m, 2H), 1.49 – 1.38 (m, 2H), 1.05 (s, 9H); ^{13}C NMR (101 MHz, $CDCl_3$): δ 135.7, 134.3, 133.4, 129.7, 127.7, 125.3, 63.9, 62.5, 32.3, 31.0, 27.2, 27.0, 26.0, 19.4; IR (film) ν / cm^{-1}: 3300, 2930, 2857, 1106, 699; MS (ESI): *m/z*: 383 [M+H$^+$]; HRMS (ESI): calcd. for $C_{24}H_{34}O_2SiNa$ [M+Na$^+$]: 405.2220; found: 405.2223.

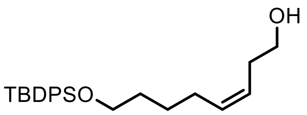

The spectroscopic properties of this compound were consistent with the data available in the literature.[6]

(*Z*)-*tert*-Butyl((8-iodooct-5-en-1-yl)oxy)diphenylsilane (28). Iodine (995 mg, 3.92 mmol) was added portionwise over 5 min to a solution of triphenylphosphine (1.04 g, 3.92 mmol) in MeCN (3 mL) and Et_2O (12 mL) at 0 °C. The resultant beige slurry was stirred for 20 min at 0 °C before imidazole (267 mg, 3.92 mmol) was added portionwise over 2 min. Next, alcohol **S7** (1.00 g, 2.61 mmol) was added dropwise over 2 min *via* syringe and the slurry was stirred at 0 °C before being warmed to room temperature and stirred for 16 h. The reaction was quenched with sat. aq. $NaHCO_3$ (10 mL) and diluted with *tert*-butyl methyl ether (10 mL). The layers were separated and the aqueous phase was extracted with *tert*-butyl methyl ether (2 × 10 mL). The combined organic portions were washed with sat. aq. $Na_2S_2O_3$ (20 mL) and brine (20 mL), dried over anhydrous Na_2SO_4, filtered, and concentrated under reduced pressure. The residue was purified by flash chromatography (1% EtOAc/hexanes) on silica to afford the title compound (1.20 g, 93% yield) as a colorless oil. ^{1}H NMR (400 MHz, $CDCl_3$): δ 7.70 – 7.64 (m, 4H), 7.43 – 7.35 (m, 6H), 5.51 (dtt, *J* = 10.4, 7.3, 1.5 Hz, 1H), 5.32 (m, 1H), 3.66 (t, *J* = 6.3 Hz, 2H), 3.12 (t, *J* = 7.3 Hz, 2H), 2.67 – 2.52 (m, 2H), 2.01 (qd, *J* = 7.3, 1.7 Hz, 2H), 1.64 – 1.52 (m, 2H), 1.51 – 1.40 (m, 2H), 1.05 (s, 9H); ^{13}C NMR (101 MHz, $CDCl_3$): δ 135.7, 134.2, 132.6, 129.7, 128.1, 127.7, 63.8, 32.3, 31.6, 27.3, 27.0, 25.9, 19.4, 5.6; IR (film) ν / cm^{-1}: 2930, 2856, 1427, 1105, 699; MS (ESI): *m/z*: 493 [M+H$^+$]; HRMS (ESI): calcd. for $C_{24}H_{33}IOSiNa$ [M+Na$^+$]: 515.1238; found: 515.1241.

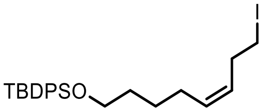

Cycloalkyne 24. A flame-dried 500-mL two-necked flask with a reflux condenser attached was charged with powdered molecular sieves 5 Å (approx. 8.5 g; dried at 150 °C under high vacuum overnight) and PhMe (300 mL). A solution of diyne **23** (210 mg, 0.624 mmol) in PhMe (10 mL) was added *via* syringe, and the suspension was degassed by bubbling argon through it for 15 min before being heated to reflux. A solution of molybdenum alkylidyne **29** (176 mg, 0.214 mmol) in PhMe (2 mL) was added dropwise over approx. 20 seconds, ensuring that the mixture remained at reflux. The mixture was stirred at reflux temperature for 1.5 h before the reaction was quenched with EtOH (10 mL). The mixture was cooled to room temperature, and filtered through a pad of Celite®, rinsing with CH_2Cl_2. The filtrate was concentrated under reduced pressure, and the residue was purified by flash chromatography (CH_2Cl_2/MeOH, 20:1) on silica to afford the title compound (160 mg, 91% yield) as a brown oil, which formed a crystalline beige solid suitable for X-ray diffraction upon brief standing at room temperature. m.p. 162 – 164 °C; ^{1}H NMR (400 MHz, CD_2Cl_2): δ 8.39 (d, *J* = 2.3 Hz, 1H), 8.29 (d, *J* = 2.3 Hz, 1H), 7.39 (t, *J* = 2.3 Hz, 1H), 4.38 (ddd, *J* = 13.7, 7.2, 4.0 Hz, 1H), 3.49 (m, 1H), 3.21 (td, *J* = 12.2, 4.4 Hz, 1H), 3.09 (dddd, *J* = 12.5, 5.6, 2.7, 0.7 Hz, 1H), 2.91 (ddd, *J* = 13.6, 5.7, 3.1 Hz, 1H), 2.71 (td, *J* = 5.3, 2.7 Hz, 2H), 2.66 (m, 1H), 2.60 (m, 1H), 2.42 – 2.30 (m, 2H), 2.26 (m, 1H), 2.15 (m, 1H), 2.11 – 1.98 (m, 2H), 1.68 – 1.55 (m, 2H), 1.46 (m, 1H),

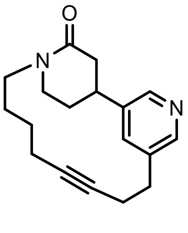

1.36 (ddt, J = 11.7, 6.6, 2.0 Hz, 1H); ^{13}C NMR (101 MHz, CD_2Cl_2): δ 168.5, 148.5, 148.4, 138.0, 136.6, 134.5, 82.0, 79.5, 47.3, 45.1, 35.7, 34.0, 32.8, 29.9, 28.4, 28.0, 20.9, 18.5; IR (film) ν / cm^{-1}: 3453, 2929, 2860, 1628, 1431, 716; MS (EI): *m/z* (%): 228 (45), 282 (100); HRMS (ESI): calcd. for $C_{18}H_{23}N_2O$ [M+H$^+$]: 283.1805; found: 283.1806.

Compound 25. *Careful degassing of the anhydrous THF by freeze-pump-thaw cycling is mandatory to avoid a side-reaction of the lithium enolate with oxygen.*

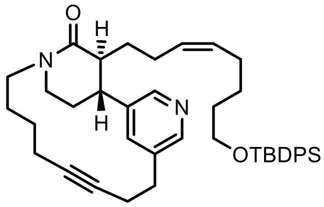

n-BuLi (1.6 M in hexanes, 0.44 mL, 0.70 mmol) was added dropwise to a stirred solution of *i*–Pr_2NH (0.10 mL, 0.71 mmol) in degassed THF (0.66 mL) at 0 °C (ice bath), and the resulting solution was stirred for 20 min. This cold solution of LDA (0.6 M in THF/hexanes, 0.47 mL, 0.28 mmol) was added dropwise to a solution of lactam **24** (58.8 mg, 0.208 mmol) and anhydrous DMPU (0.25 mL, 2.1 mmol) in degassed THF (1.2 mL) at −78 °C. The resulting very dark amber solution was stirred at −78 °C for 1 h before a solution of iodide **28** (205 mg, 0.416 mmol) in degassed THF (0.70 mL) was added *via* syringe. Stirring was continued at −78 °C for 3 h before sat. aq. $NaHCO_3$ (10 mL) and EtOAc (10 mL) were added, and the mixture was warmed to room temperature. The layers were separated and the aqueous phase was extracted with EtOAc (3 × 10 mL). The combined organic layers were dried over anhydrous Na_2SO_4, filtered, and concentrated under reduced pressure. Purification of the residue by flash chromatography (CH_2Cl_2/MeOH, 40:1) on silica afforded the title compound admixed with traces of remaining DMPU, which were removed by aqueous extraction from *tert*-butyl methyl ether (20 mL, 3 × 10 mL H_2O) to furnish the pure title compound as a pale yellowish gum (121 mg, 90% yield). ^{1}H NMR (600 MHz, CD_2Cl_2): δ 8.43 – 8.34 (m, 1H), 8.27 (s, 1H), 7.68 – 7.62 (m, 4H), 7.45 – 7.32 (m, 6H), 5.43 – 5.35 (m, 2H), 4.36 (dt, J = 13.7, 5.6 Hz, 1H), 3.62 (td, J = 6.4, 1.9 Hz, 2H), 3.27 (t, J = 4.0 Hz, 1H), 3.20 (td, J = 12.4, 4.6 Hz, 1H), 3.06 – 2.99 (m, 1H), 2.87 (ddd, J = 13.7, 6.0, 3.0 Hz, 1H), 2.74 – 2.61 (m, 2H), 2.57 (ddd, J = 13.7, 12.1, 2.9 Hz, 1H), 2.40 – 2.30 (m, 3H), 2.25 – 2.12 (m, 3H), 2.08 – 1.87 (m, 5H), 1.71 – 1.60 (m, 4H), 1.56 – 1.44 (m, 3H), 1.41 – 1.33 (m, 3H), 1.02 (s, 9H); ^{13}C NMR (151 MHz, CD_2Cl_2): δ 171.7, 148.7, 148.5, 138.6, 136.6, 135.9, 134.7, 134.6, 131.2, 129.9, 129.1, 128.0, 81.8, 79.6, 64.3, 54.2, 54.0, 53.8, 53.7, 53.5, 47.6, 45.0, 44.4, 37.9, 34.1, 32.9, 32.6, 28.5, 28.2, 27.4, 27.0, 26.3, 26.0, 26.0, 21.0, 19.5, 18.5; ^{29}Si NMR (119 MHz, CD_2Cl_2) δ −5.3; IR (film) ν / cm^{-1}: 2930, 2857, 1635, 1429, 1109, 704; MS (ESI): *m/z*: 649 [M+H$^+$]; HRMS (ESI): calcd. for $C_{42}H_{55}N_2O_2Si$ [M+H$^+$]: 649.4184; found: 649.4182.

The stereochemical assignment was proven by comparison with the compound formed by ring-closing alkyne metathesis of diyne **26** which was carried out in analogy to the procedure described above (61% yield, 17 mg). The spectral data of both samples were identical.

Compound 26. *Careful degassing the anhydrous THF by freeze-pump-thaw cycling is mandatory to avoid a side-reaction of the lithium enolate with oxygen.*

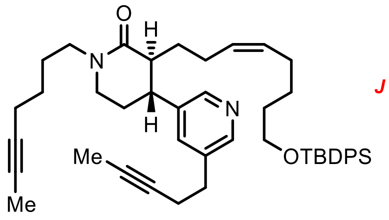

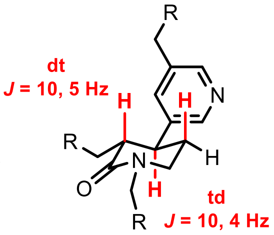

n-BuLi (1.6 M in hexanes, 0.44 mL, 0.70 mmol) was added dropwise to a stirred solution of *i*–Pr_2NH (0.10 mL, 0.71 mmol) in degassed THF (0.66 mL) at 0 °C (ice bath), and the resulting solution was stirred for 20 min. This solution of LDA (0.6 M in THF/hexanes, 0.47 mL, 0.28 mmol) was added dropwise to a solution of lactam **23** (72.1 mg, 0.214 mmol) and anhydrous DMPU (0.16 mL, 1.3 mmol) in degassed THF (1.2 mL) at −78 °C. The resulting very dark amber solution was stirred at −78 °C for 1 h before a solution of iodide **28** (191 mg, 0.388 mmol) in degassed THF (0.70 mL) was added *via* syringe. Stirring was continued at −78 °C for 3 h before sat. aq. $NaHCO_3$ (10 mL) and EtOAc (10 mL) were added, and the mixture was warmed to room temperature. The layers were separated and the aqueous phase was extracted with EtOAc (3 × 10 mL). The combined organic layers were dried over anhydrous Na_2SO_4, filtered, and concentrated under reduced pressure. Purification of the residue by flash chromatography (CH_2Cl_2/MeOH, 40:1 – 20:1) on silica afforded the title compound (128 mg, 85% yield) as a light amber oil. ^{1}H NMR (600 MHz, CD_2Cl_2): δ 8.33 (d, *J* = 2.1 Hz, 1H), 8.31 (d, *J* = 2.2 Hz, 1H), 7.70 – 7.63 (m, 4H), 7.45 – 7.34 (m, 7H), 5.31 – 5.19 (m, 2H), 3.66 (t, *J* = 6.4 Hz, 2H), 3.50 – 3.18 (m, 4H), 2.92 (td, *J* = 10.4, 3.9 Hz, 1H), 2.75 (t, *J* = 7.2 Hz, 2H), 2.53 (dt, *J* = 9.9, 4.8 Hz, 1H), 2.44 – 2.36 (m, 2H), 2.20 – 2.12 (m, 2H), 2.10 – 1.87 (m, 6H), 1.82 – 1.70 (m, 1H), 1.75 (t, *J* = 2.6 Hz, 3H), 1.73 (t, *J* = 2.5 Hz, 3H), 1.69 – 1.57 (m, 2H), 1.53 – 1.33 (m, 7H), 1.03 (s, 9H); ^{13}C NMR (151 MHz, CD_2Cl_2): δ 171.1, 148.9, 147.6, 139.1, 136.6, 135.9, 134.6, 134.6, 130.6, 129.9, 129.7, 128.0, 79.1, 78.0, 77.2, 75.9, 64.4, 47.2, 47.1, 47.0, 41.2, 32.8, 32.7, 31.0, 29.9, 27.3, 27.0, 26.8, 26.6, 26.4, 24.6, 21.0, 19.5, 18.8, 3.5, 3.5; IR (film) ν / cm^{-1}: 2930, 2857, 1637, 1490, 1429, 1110, 823, 741, 704, 614, 505; MS (ESI): *m/z*: 701 [$M+H^+$]; HRMS (ESI): calcd. for $C_{46}H_{61}N_2O_2Si$ [$M+H^+$]: 701.4497; found: 701.4493.

Compound 30. $NaBH_4$ (52.2 mg, 1.38 mmol) was added portionwise to a solution of $Ni(OAc)_2•4H_2O$ (312 mg, 1.26 mmol) in EtOH (10 mL) at 0 °C. The resulting black suspension was vigorously stirred for 30 min at 0 °C before ethylenediamine (0.34 mL, 5.0 mmol) was added *via* syringe. The suspension was warmed to room temperature and stirred for another 30 min. The suspension was transferred *via* cannula to a solution of alkyne **25** (203 mg, 0.314 mmol) in EtOH (8 mL) at 0 °C. A balloon filled with hydrogen was connected to the flask and hydrogen was bubbled through the black suspension for 2 min. The suspension was then stirred for 16 h at room temperature under the hydrogen atmosphere before it was filtered through a pad of silica, rinsing with CH_2Cl_2. The filtrate was concentrated under reduced pressure and the residue was purified by flash chromatography (1% MeOH/CH_2Cl_2) on silica to

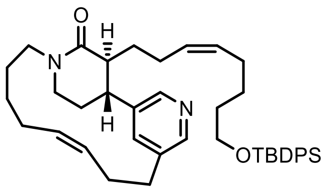

afford the title compound (192 mg, 94% yield) as a colorless oil. ^{1}H NMR (600 MHz, CD_2Cl_2): δ 8.39 (s, 1H), 8.18 (s, 1H), 7.68 – 7.63 (m, 4H), 7.43 – 7.34 (m, 6H), 7.32 (d, *J* = 2.2 Hz, 1H), 5.47 – 5.35 (m, 3H), 5.24 (td, *J* = 9.7, 4.7 Hz, 1H), 4.28 (ddd, *J* = 13.5, 8.8, 4.8 Hz, 1H), 3.62 (td, *J* = 6.4, 1.6 Hz, 2H), 3.25 (t, *J* = 3.8 Hz, 1H), 2.95 – 2.88 (m, 2H), 2.78 – 2.72 (m, 1H), 2.67 (ddd, *J* = 13.6, 11.7, 4.2 Hz, 1H), 2.63 – 2.55 (m, 1H), 2.41 – 2.26 (m, 3H), 2.25 – 2.13 (m, 2H), 2.05 – 1.93 (m, 5H), 1.73 – 1.63 (m, 2H), 1.54 – 1.33 (m, 6H), 1.11 – 1.05 (m, 1H), 1.02 (s, 9H), 0.86 – 0.75 (m, 1H); ^{13}C NMR (151 MHz, CD_2Cl_2): δ 171.9, 148.5 (*two signals*), 138.1, 136.8, 135.9, 134.6, 133.5, 131.2, 130.5, 130.0, 129.9, 129.1, 128.0, 64.2, 47.6, 44.2, 43.9, 36.9, 33.7, 32.6, 32.2, 27.6, 27.4 (*two signals*), 27.2, 27.0, 26.9, 26.3, 26.0, 25.1, 19.5; IR (film) ν / cm^{-1}: 2930, 2857, 2360, 1637, 1110, 704; MS (ESI): *m/z*: 647 [M+H$^+$]; HRMS (ESI): calcd. for $C_{42}H_{55}N_2O_2Si$ [M+H$^+$]: 647.4027; found: 647.4026.

Compound 32. In a 10-mL Schlenk tube, 1,1,3,3-tetramethyldisiloxane (52 µL, 0.29 mmol) was added at

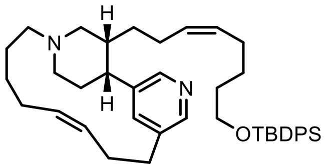

room temperature to a solution of lactam **30** (47.0 mg, 72.4 µmol) and $[IrCl(CO)(PPh_3)_2]$ (6.7 mg, 8.6 µmol) in toluene (1.4 mL). The resulting yellow solution was stirred for 2 h at room temperature, when a colorless solution had formed. MeOH (0.20 mL) was added dropwise, which caused effervescence of the mixture after brief stirring. Once the effervescence had ceased, the mixture was transferred to a solution of $NaBH_3CN$ (12 mg, 0.19 mmol) and AcOH (8.5 µL, 0.15 mmol) in methanol (0.20 mL), rinsing with an additional amount of methanol (0.20 mL). The resulting mixture was stirred at room temperature for 45 min before the reaction was quenched with sat. aq. Na_2CO_3 (10 mL). The mixture was diluted with *tert*-butyl methyl ether (10 mL), and the layers were separated. The aqueous phase was extracted with *tert*-butyl methyl ether (4 × 5 mL) and the combined organic portions were dried over anhydrous Na_2SO_4, filtered, and concentrated under reduced pressure. Purification of the residue by flash chromatography (CH_2Cl_2/MeOH, 20:1 – 10:1) on silica afforded the title compound (29.0 mg, 63% yield) as a light yellowish gum. ^{1}H NMR (600 MHz, C_6D_6): δ 8.45 (br s, 1H), 8.43 (d, *J* = 2.0 Hz, 1H), 8.42 (d, *J* = 2.2 Hz, 1H), 7.83 – 7.78 (m, 4H), 7.27 – 7.23 (m, 6H), 5.60 (q, *J* = 8.3 Hz, 1H), 5.40 (td, *J* = 9.8, 5.5 Hz, 1H), 5.42 – 5.34 (m, 1H), 5.33 – 5.26 (m, 1H), 3.70 (t, *J* = 6.4 Hz, 2H), 2.81 (ddd, *J* = 9.3, 4.9, 1.8 Hz, 1H), 2.52 (dt, *J* = 13.6, 7.8 Hz, 1H), 2.49 (ddt, *J* = 11.6, 8.1, 4.0 Hz, 1H), 2.45 (t, *J* = 11.0 Hz, 1H), 2.41 (dd, *J* = 8.5, 5.6 Hz, 1H), 2.39 – 2.37 (m, 2H), 2.32 – 2.26 (m, 1H), 2.27 (ddd, *J* = 11.4, 9.6, 6.0 Hz, 1H), 2.25 – 2.18 (m, 1H), 2.19 (dd, *J* = 11.0, 6.9 Hz, 1H), 2.09 – 2.01 (m, 1H), 2.05 – 2.00 (m, 2H), 2.05 – 1.96 (m, 1H), 1.95 (ddd, *J* = 11.6, 7.2, 4.6 Hz, 1H), 1.96 – 1.87 (m, 1H), 1.89 (dddd, *J* = 13.1, 9.4, 5.8, 3.6 Hz, 1H), 1.87 – 1.78 (m, 1H), 1.83 – 1.75 (m, 1H), 1.68 – 1.61 (m, 1H), 1.61 (ddt, *J* = 9.3, 8.2, 6.2 Hz, 2H), 1.45 (tt, *J* = 9.3, 6.0 Hz, 2H), 1.38 – 1.31 (m, 3H), 1.20 (s, 9H), 1.11 – 1.03 (m, 1H), 0.91 (ddt, *J* = 13.5, 9.1, 6.6 Hz, 1H); ^{13}C NMR (151 MHz, C_6D_6): δ 149.6, 147.9, 139.4, 137.4, 137.1, 136.1, 134.5, 131.4, 130.4, 130.0, 129.7, 129.5, 128.2, 128.1 (*two signals*), 127.9, 64.2, 56.9, 53.7, 46.2, 37.0, 35.5, 32.9

(*two signals*), 32.7, 31.3, 28.9, 27.6, 27.4, 27.2 (*two signals*), 26.4 (*two signals*), 25.0, 19.5; IR (film) ν / cm^{-1}: 2929, 2857, 1428, 1110, 702, 505; MS (EI): *m/z* (%): 281 (53), 577 (100), 634 (53); HRMS (ESI): calcd. for $C_{42}H_{59}N_2OSi$ [$M+H^+$]: 635.4391; found: 635.4392.

Compound S8. Following the same procedure, lactam **26** was converted into a separable mixture of isomeric piperidine derivatives **S8**. Which of these products is the *cis*- and which the *trans*-isomer could not be unambiguously be determined by NMR spectroscopy due to significant signal overlap and line broadening, even though spectra were recorded inn different solvents (C_6D_6, [D_6]-acetone, CD_2Cl_2) at different temperatures.

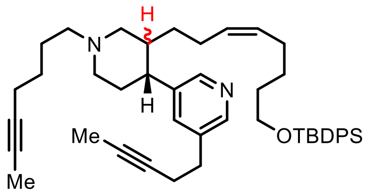

Spectral data of the major isomer (35.7 mg, 37% yield): ^{1}H NMR (600 MHz, CD_2Cl_2): δ 8.27 (d, *J* = 2.1 Hz, 1H), 8.25 (d, *J* = 2.1 Hz, 1H), 7.67 – 7.64 (m, 4H), 7.45 – 7.36 (m, 7H), 5.30 – 5.23 (m, 1H), 5.18 – 5.11 (m, 1H), 3.65 (t, *J* = 6.4 Hz, 2H), 3.12 (br d, *J* = 8.9 Hz, 1H), 2.99 (br d, *J* = 11.2 Hz, 1H), 2.73 (td, *J* = 7.0, 1.4 Hz, 2H), 2.42 – 2.37 (m, 2H), 2.37 – 2.31 (m, 2H), 2.20 – 2.11 (m, 3H), 2.00 – 1.88 (m, 4H), 1.86 – 1.76 (m, 2H), 1.75 (t, *J* = 2.6 Hz, 3H), 1.75 – 1.72 (br m, 2H), 1.73 (t, *J* = 2.6 Hz, 3H), 1.71 – 1.64 (br m, 1H), 1.62 – 1.56 (m, 2H), 1.56 – 1.46 (m, 4H), 1.41 – 1.33 (m, 2H), 1.17 – 1.09 (m, 1H), 1.04 (s, 9H), 1.02 – 0.94 (m, 1H); ^{13}C NMR (151 MHz, CD_2Cl_2): δ 148.3, 148.1, 140.4, 136.3, 135.9, 135.2, 134.6, 130.3, 129.90, 129.87, 128.0, 79.4, 78.1, 77.0, 75.8, 64.3, 59.9, 58.7, 54.4, 46.9, 40.9, 35.3, 32.8, 32.6, 32.2, 27.5, 27.3, 27.0, 26.6, 26.3, 24.7, 21.0, 19.5, 19.0, 3.54, 3.52; IR (film) ν / cm^{-1}: 2930, 2857, 1428, 1110, 823, 703, 614, 505; HRMS (ESI): calcd. for $C_{46}H_{63}N_2OSi$ [$M+H^+$]: 687.4704; found: 687.4708.

Spectral data of the minor isomer (17.8 mg, 18% yield): ^{1}H NMR (600 MHz, CD_2Cl_2): δ 8.31 – 8.20 (m, 2H), 7.68 – 7.63 (m, 4H), 7.44 – 7.40 (m, 2H), 7.39 – 7.35 (m, 5H), 5.28 – 5.21 (m, 1H), 5.15 – 5.07 (m, 1H), 3.64 (td, *J* = 6.4, 1.0 Hz, 2H), 3.06 – 2.95 (m, 2H), 2.87 – 2.80 (m, 1H), 2.74 (t, *J* = 7.2 Hz, 2H), 2.42 – 2.37 (m, 2H), 2.37 – 2.23 (m, 2H), 2.16 – 2.11 (m, 2H), 2.10 – 1.99 (m, 3H), 1.97 – 1.87 (m, 3H), 1.84 – 1.79 (m, 1H), 1.74 (t, *J* = 2.6 Hz, 3H), 1.73 (t, *J* = 2.5 Hz, 3H), 1.72 – 1.69 (br m, 1H), 1.67 – 1.62 (m, 1H), 1.57 – 1.46 (m, 6H), 1.42 – 1.30 (m, 2H), 1.03 (s, 9H), 0.89 – 0.79 (m, 2H); ^{13}C NMR (151 MHz, CD_2Cl_2): δ 147.7, 147.6, 139.6, 135.9, 134.6, 130.1, 130.1, 129.9, 128.0, 79.4, 78.1, 77.0, 75.7, 64.3, 58.5, 57.1, 54.9, 43.3, 40.3, 32.8, 32.6, 27.5, 27.3, 27.0, 26.7, 26.3, 25.8, 25.65, 25.63, 21.0, 19.5, 19.0, 3.54, 3.53; IR (film) ν / cm^{-1}: 2929, 2856, 1715, 1429, 1258, 1110, 823, 800, 703, 614, 505; HRMS (ESI): calcd. for $C_{46}H_{63}N_2OSi$ [$M+H^+$]: 687.4704; found: 687.4706.

Attempted ring closing metathesis on treatment of either isomer with catalytic or even over-stoichiometric amounts of the alkylidyne catalyst **29** met with failure. Since tertiary amines were previously shown to be

compatible with catalysts of this type,[9] the lack of reactivity is almost certainly rooted in an unfavorable conformation adopted by these substrates rather than in chemoselectivity issues.

Compound S9. In a 10-mL Schlenk tube, TBAF (1.0 M in THF, 0.19 mL, 0.19 mmol) was added to a solution of silyl ether **32** (38.8 mg, 61.1 µmol) in THF (0.61 mL) at room temperature. The resulting light amber solution was stirred for 3.5 h before it was poured into a mixture comprised of sat. aq. Na_2CO_3 (10 mL) and EtOAc (10 mL). The layers were separated and the aqueous phase was extracted with EtOAc (4 × 10 mL). The combined organic layers were dried over anhydrous Na_2SO_4, filtered, and concentrated under reduced pressure. Purification of the residue by flash chromatography (CH_2Cl_2/MeOH, 7:1 – 4:1) on silica afforded the title compound (21.1 mg, 87% yield) as a pale red gum. ^{1}H NMR (400 MHz, C_6D_6): δ 8.47 (t, *J* = 2.1 Hz, 1H), 8.35 (d, *J* = 2.1 Hz, 1H), 8.30 (d, *J* = 2.1 Hz, 1H), 5.66 – 5.53 (m, 1H), 5.47 – 5.25 (m, 3H), 3.66 (qt, *J* = 10.6, 6.2 Hz, 2H), 3.38 – 3.10 (m, 1H), 2.84 (ddd, *J* = 9.3, 4.8, 2.0 Hz, 1H), 2.54 – 2.44 (m, 2H), 2.42 – 2.31 (m, 4H), 2.30 – 2.06 (m, 6H), 2.06 – 1.94 (m, 2H), 1.94 – 1.72 (m, 5H), 1.67 – 1.53 (m, 4H), 1.47 (dq, *J* = 9.8, 6.9 Hz, 2H), 1.38 – 1.30 (m, 2H), 0.90 (td, *J* = 7.3, 6.5, 5.1 Hz, 2H); ^{13}C NMR (101 MHz, C_6D_6): δ 149.0, 147.4, 139.6, 138.0, 137.5, 131.3, 130.5, 129.5 (*two signals*), 62.2, 57.0, 53.8, 46.3, 36.5, 36.0, 33.2, 32.9, 32.8, 31.1, 28.9, 27.6 (*two signals*), 27.2, 26.5 (*two signals*), 25.3; IR (film) ν / cm^{-1}: 3291, 2926, 2857, 1434, 724; MS (ESI): *m/z*: 397 [M+H$^+$]; HRMS (ESI): calcd. for $C_{26}H_{41}N_2O$ [M+H$^+$]: 397.3213; found: 397.3215.

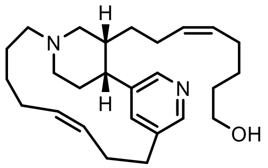

***epi*-Tetradehydrohalicyclamine B (7).** Iodine (20.2 mg, 79.6 µmol) was added to a solution of triphenylphosphine (21.0 mg, 80.1 µmol) in MeCN (0.50 mL) and Et_2O (0.50 mL) at 0 °C. The resultant beige slurry was stirred for 20 min at 0 °C before imidazole (5.4 mg, 79 µmol) was added. After stirring for 10 min, a solution of alcohol **S9** (19.7 mg, 49.7 µmol) in MeCN/Et_2O (0.40/0.60 mL) was added, and the resulting mixture was warmed to room temperature and stirred for 40 min. The light amber solution was diluted with *tert*-butyl methyl ether (10 mL) and washed with water (2 × 8 mL) and sat. aq. $Na_2S_2O_3$ (5 mL). The aqueous phases were extracted with *tert*-butyl methyl ether (4 × 5 mL), and the combined organic layers were dried over anhydrous Na_2SO_4, filtered, and concentrated under reduced pressure. Purification of the residue by flash chromatography (CH_2Cl_2/MeOH, 10:1 – 5:1) on silica afforded alkyl iodide **33** (19.8 mg, 79% yield) as a light amber gum, which was used immediately in the next step. MS (ESI): *m/z*: 507 [M+H$^+$]; HRMS (ESI): calcd. for $C_{26}H_{40}IN_2$ [M+H$^+$]: 507.2231; found: 507.2236.

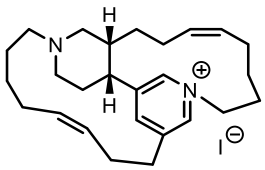

In a 50-mL two-necked flask equipped with a reflux condenser, a solution of iodide **33** in MeCN (19 mL) was stirred at reflux temperature for 24 h. The mixture was cooled to room temperature, and the solvent was removed under reduced pressure. Purification of the residue by flash chromatography (CH_2Cl_2/MeOH,

6:1 – 4:1) on silica afforded the title compound (9.6 mg, 49% yield) as a pale yellowish gum, which formed a pale yellowish solid when treated with dichloromethane. ^{1}H NMR (600 MHz, D_3COD): δ 9.42 (s, 1H), 9.09 (s, 1H), 8.77 (s, 1H), 5.77 – 5.71 (m, 1H), 5.51 – 5.42 (m, 3H), 4.89 – 4.84 (m, 1H), 4.67 (td, *J* = 13.3, 4.0 Hz, 1H), 3.59 (ddd, *J* = 9.9, 5.1, 1.7 Hz, 1H), 3.11 (ddd, *J* = 14.8, 8.7, 7.6 Hz, 1H), 2.92 – 2.82 (m, 2H), 2.67 – 2.53 (m, 6H), 2.53 – 2.45 (m, 1H), 2.40 – 2.13 (m, 6H), 2.13 – 1.97 (m, 3H), 1.80 – 1.71 (m, 1H), 1.71 – 1.49 (m, 6H), 1.35 – 1.27 (m, 1H), 0.24 – 0.15 (m, 1H), 0.08 – 0.00 (m, 1H); ^{13}C NMR (151 MHz, D_3COD): δ 149.4, 146.2, 145.6, 144.3, 141.6, 132.1, 130.9, 130.5, 130.1, 60.0, 57.6, 54.7, 46.9, 37.2, 36.4, 35.0, 33.6, 30.5, 30.3, 28.8, 28.3, 27.7, 26.7, 26.5, 25.6, 25.2; IR (film) ν / cm^{-1}: 3441 (br), 3006, 2926, 2858, 2803, 1624, 1494, 1461, 724, 707; MS (ESI^{+}): *m/z*: 379 [M^{+}]; MS (ESI^{-}): *m/z*: 127 [I^{-}]; HRMS (ESI^{+}): calcd. for $C_{26}H_{39}N_2$ [M^{+}]: 379.3108; found: 379.3109.

As discussed in the isolation paper of *epi*-tetradehydrohalicyclamine B,[7] the magnetic resonance at unusually low chemical shift of one of the hydrogen atoms each at C13 and C8 is particularly indicative of the *cis*-configuration of the piperidine core.

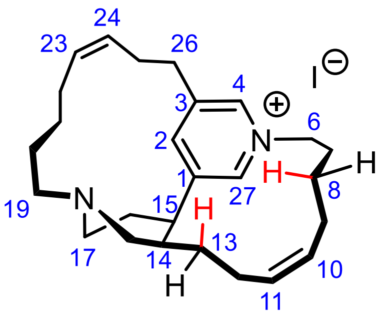

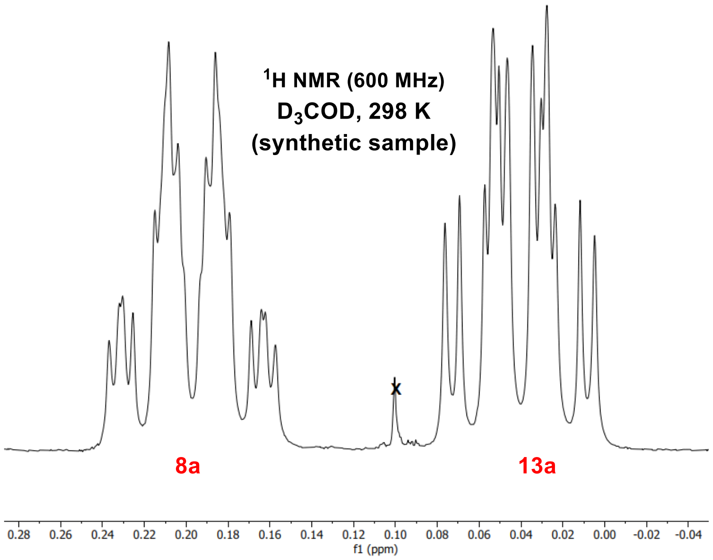

Table S1. Comparison of the NMR Data of Natural[7] (700 MHz) and Synthetic (600 MHz) *epi*-tetradehydrohalicyclamine B (**7**) in [D_4]-MeOH; numbering scheme as shown in the insert

Position	δ_C natural	δ_C synthetic	$\Delta\delta_C$	δ_H (*J*/Hz) natural	δ_H (*J*/Hz) synthetic	$\Delta\delta_H$
1	146.2	146.2	0.0	-	-	-
2	149.4	149.4	0.0	9.44 s	9.42 s	–0.02
3	145.7	145.6	–0.1	-	-	-
4	141.5	141.6	+0.1	8.73 s	8.77 s	+0.04
5 (N)	-	-	-	-	-	-
6	60.0	60.0	0.0	4.64 ddd (13.4, 13.4, 4.1)	4.67 td (13.3, 4.0)	+0.03
				4.84 ddd (13.4, 5.6, 1.5)	4.85 m	+0.01
7	30.2	30.3	+0.1	2.02 br dd (14.6, 13.3)	2.02 m	0.00
				2.23 m	2.24 m	+0.01
8	26.4	26.5	+0.1	0.20 ddddd (14.6, 13.3, 13.1, 4.0, 2.9)	0.20 m	0.00
				1.54 ddddd (14.6, 14.0, 4.1, 4.0, 4.0)	1.54 m	0.00
9	25.5	25.6	+0.1	1.62 dddd (13.2, 13.2, 5.5, 4.0)	1.63 m	+0.01
				2.58 dddd (14.0, 13.0, 9.0, 4.1)	2.59 m	+0.01
10	130.1	130.1	0.0	5.48 m	5.47 m	-0.01
11	130.9	130.9	0.0	5.45 m	5.46 m	+0.01
12	25.0	25.2	+0.2	1.68 tt (13.0, 4.3)	1.68 m	0.00
				2.58 dddd (14.0, 14.0, 8.5, 4.0)	2.59 m	+0.01
13	35.0	35.0	0.0	0.02 ddd (9.8, 5.2, 1.4)	0.04 m	+0.02
				1.31 dddd (14.3, 14.3, 4.0, 3.0)	1.31 m	0.00
14	37.1	37.2	+0.1	2.30 m	2.30 m	0.00
15	36.4	36.4	0.0	3.56 ddd (9.8, 5.2, 1.4)	3.59 ddd (9.9, 5.1, 1.7)	+0.03
16	30.5	30.5	0.0	2.09 dddd (14.4, 10.3, 7.6, 1.4)	2.08 m	–0.01
				2.32 m	2.32 m	0.00
17	46.8	46.9	+0.1	2.55 ddd (11.6, 10.3, 5.2)	2.56 m	+0.01

				2.86 ddd (11.6, 7.6, 2.8)	2.87 m	+0.01
18 (N)	-	-	-	-	-	-
19	57.5	57.6	+0.1	2.55 m	2.56 m	+0.01
				2.62 m	2.62 m	0.00
20	26.6	26.7	+0.1	1.58 m	1.56 m	–0.02
				1.68 m	1.66 m	-0.02
21	28.3	28.3	0.0	1.55 m	1.56 m	+0.01
				1.74 m	1.73 m	-0.01
22	27.7	27.7	0.0	2.04 dddd (13.5, 10.2, 7.8, 5.5)	2.05 m	+0.01
				2.20 m	2.22 m	+0.02
23	132.2	132.1	–0.1	5.75 ddd (10.1, 8.2, 8.2)	5.74 m	–0.01
24	130.4	130.5	+0.1	5.48 m	5.48 m	0.00
25	28.8	28.8	0.0	2.49 dddd (15.2, 10.0, 8.2, 4.8)	2.49 m	0.00
				2.56 dddd (15.2, 8.8, 8.2, 7.0)	2.57 m	+0.01
26	33.6	33.6	0.0	2.85 ddd (14.6, 8.8, 4.8)	2.85 m	0.00
				3.08 ddd (14.6, 8.2, 8.2)	3.11 ddd (14.8, 8.7, 7.6)	+0.03
27	144.2	144.3	+0.1	9.04 s	9.09 s	+0.05
28	54.8	54.7	–0.1	2.35 m	2.32 m	–0.03
				2.35 m	2.34 m	–0.01

References

(1) Krasovskiy, A.; Knochel, P. A LiCl-Mediated Br/Mg Exchange Reaction for the Preparation of Functionalized Aryl- and Heteroarylmagnesium Compounds from Organic Bromides. *Angew. Chem. Int. Ed.* **2004**, *43*, 3333.

(2) Garnier, E. C.; Liebeskind, L. S. Organometallic Enantiomeric Scaffolding: General Access to 2-Substituted Oxa- and Azabicyclo[3.2.1]octenes via a Brønsted Acid Catalyzed [5 + 2] Cycloaddition Reaction. *J. Am. Chem. Soc.* **2008**, *130*, 7449.

(3) Villar, I. S. d.; Gradillas, A.; Domínguez, G.; Pérez-Castells, J. Nitrogen ylide-mediated cyclopropanation of lactams and lactones. *Tetrahedron Lett.* **2010**, *51*, 3095.

(4) Yiannakas, E.; Grimes, M. I.; Whitelegge, J. T.; Fürstner, A.; Hulme, A. N. An Alkyne-Metathesis-Based Approach to the Synthesis of the Anti-Malarial Macrodiolide Samroiyotmycin A. *Angew. Chem. Int Ed.* **2021**, *60*, 18504.

(5) Brandt, D.; Bellosta, V.; Cossy, J. Stereoselective Synthesis of Conjugated Trienols from Allylic Alcohols and 1-Iodo-1,3-dienes. *Org. Lett.* **2012**, *14*, 5594.

(6) Nikas, S. P.; D'Souza, M.; Makriyannis, A. Enantioselective synthesis of (10*S*)- and (10*R*)-methyl-anandamides. *Tetrahedron* **2012**, *68*, 6329.

(7) Esposito, G.; Mai, L. H.; Longeon, A.; Mangoni, A.; Durieu, E.; Meijer, L.; Van Soest, R.; Costantino, V.; Bourguet-Kondracki, M.-L. A Collection of Bioactive Nitrogen-Containing Molecules from the Marine Sponge *Acanthostrongylophora ingens*. *Mar. Drugs* **2019**, *17*, 472.

(8) Meng, Z.; Spohr, S. M.; Tobegen, S.; Farès, C.; Fürstner, A. A Unified Approach to Polycyclic Alkaloids oft he Ingenamine Estate: Total Syntheses of Keramaphidin B, Ingenamine, and Nominal Njaoamine I. *J. Am. Chem. Soc*. **2021**, *143*, 13489-13494.

Copies of NMR spectra

^{1}H NMR spectrum of 16 (400 MHz, CD_2Cl_2)

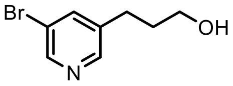

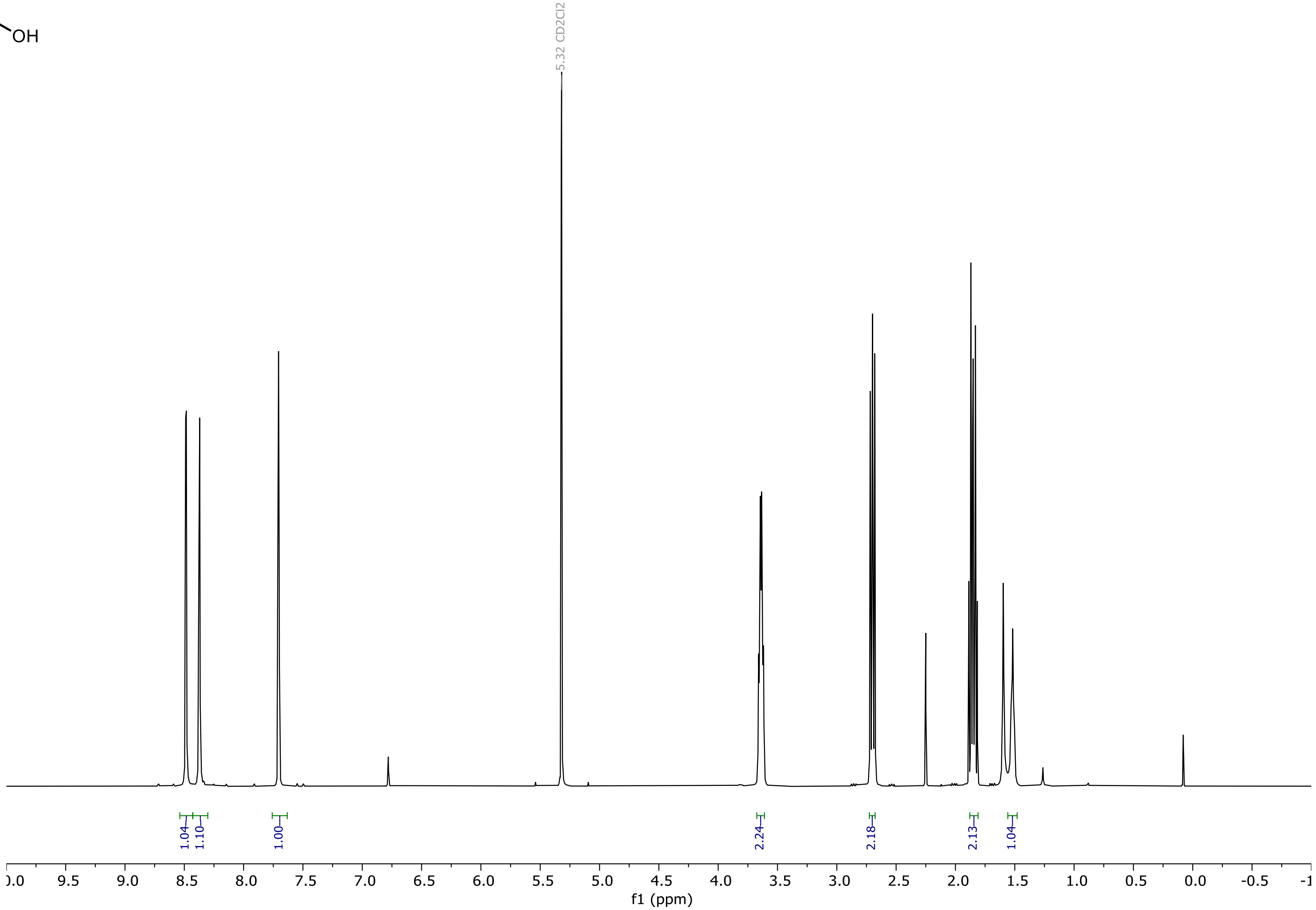

^{13}C NMR spectrum of 16 (101 MHz, CD_2Cl_2)

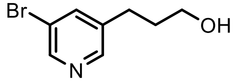

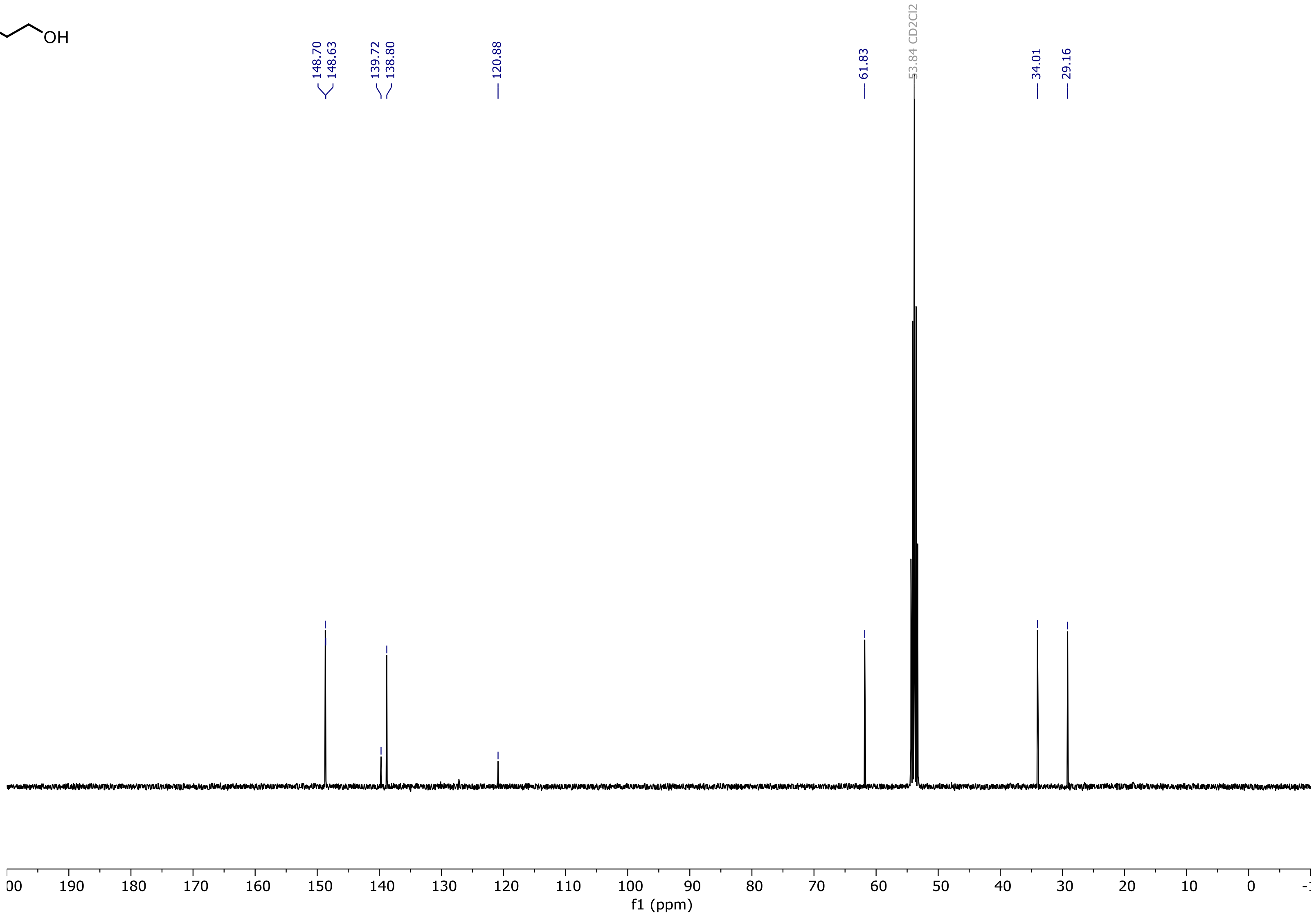

^{1}H NMR spectrum of S1 (400 MHz, CD_2Cl_2)

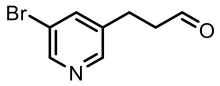

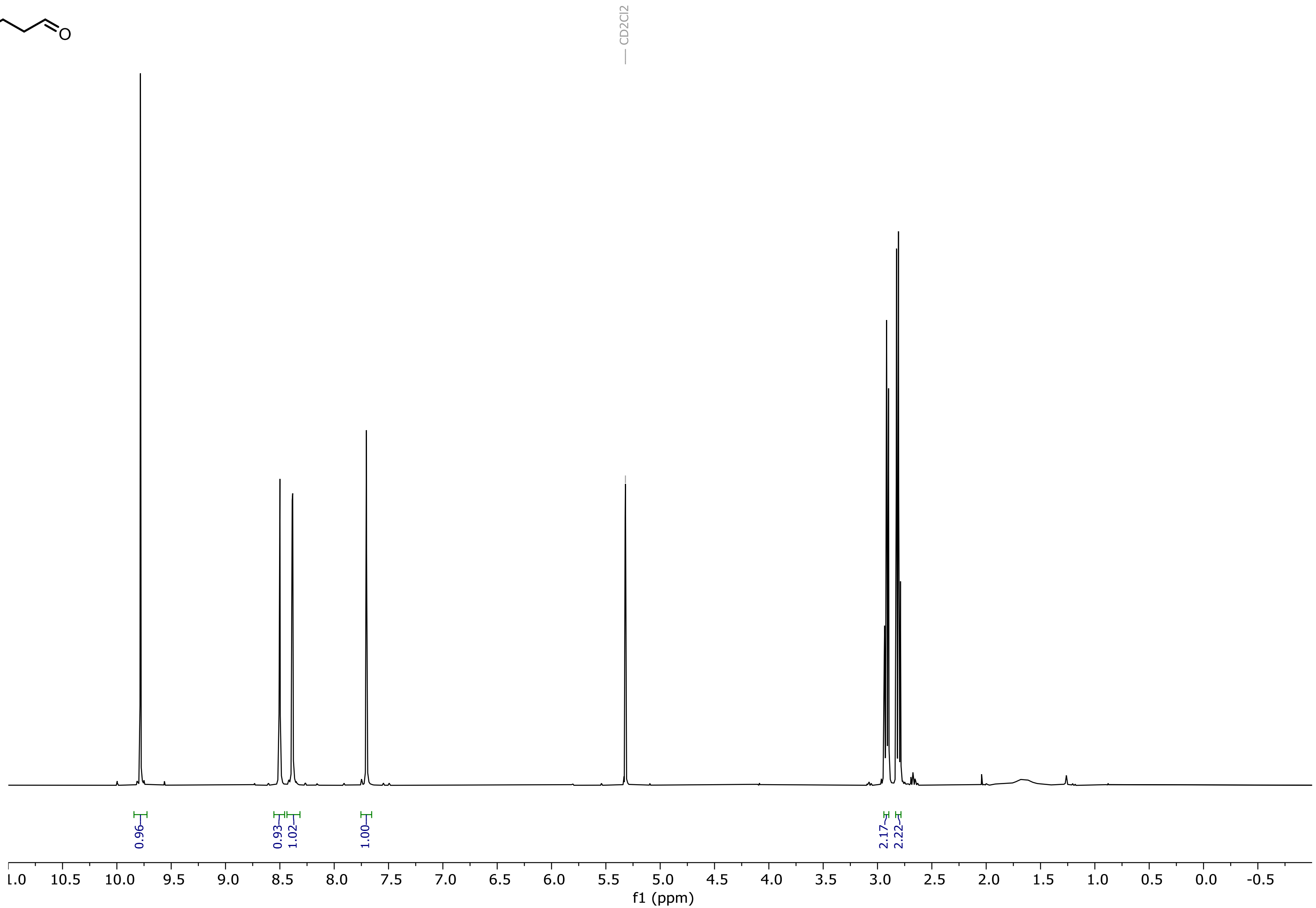

^{13}C NMR spectrum of S1 (101 MHz, CD_2Cl_2)

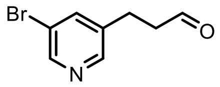

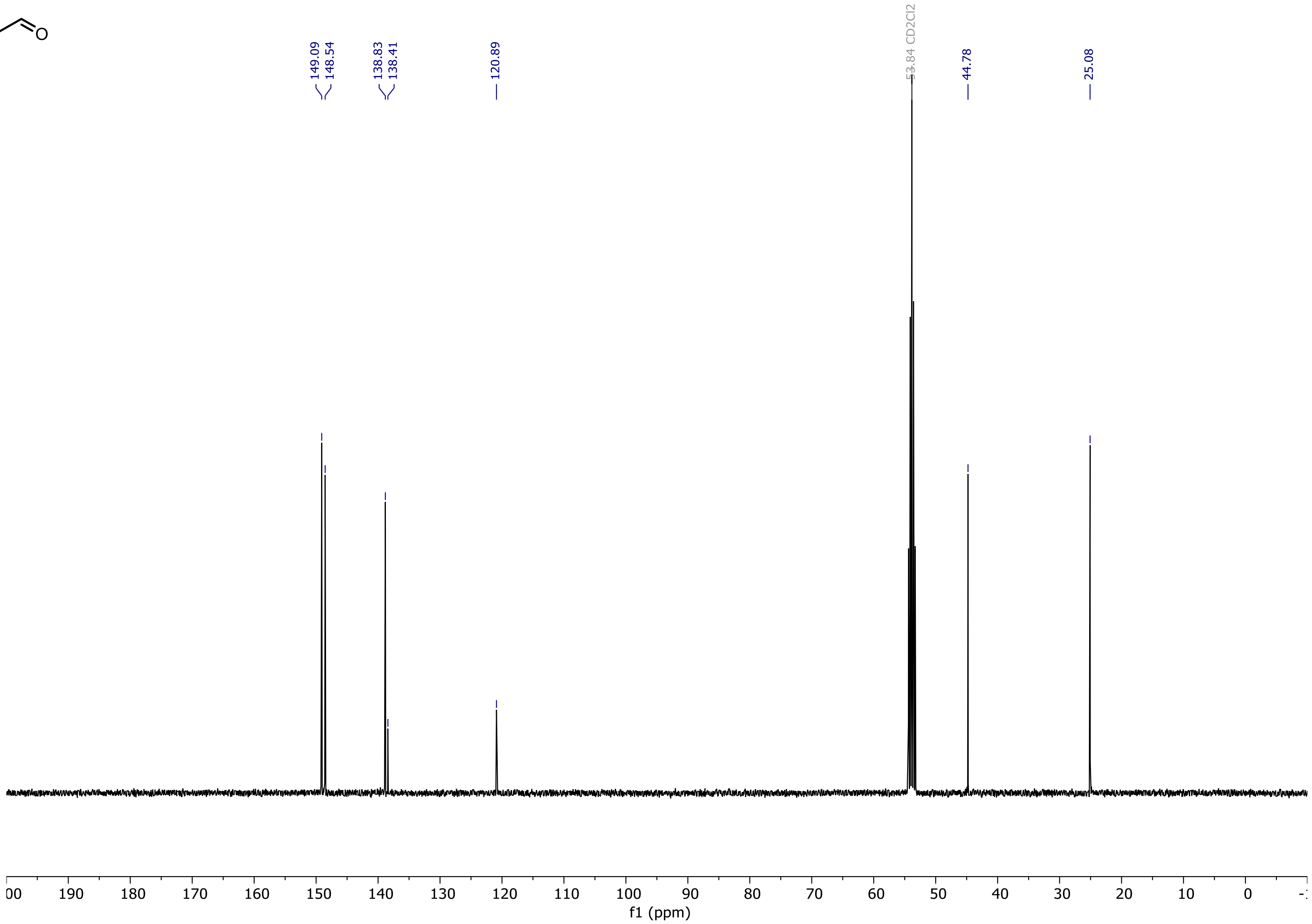

^{1}H NMR spectrum of S2 (400 MHz, CD_2Cl_2)

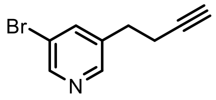

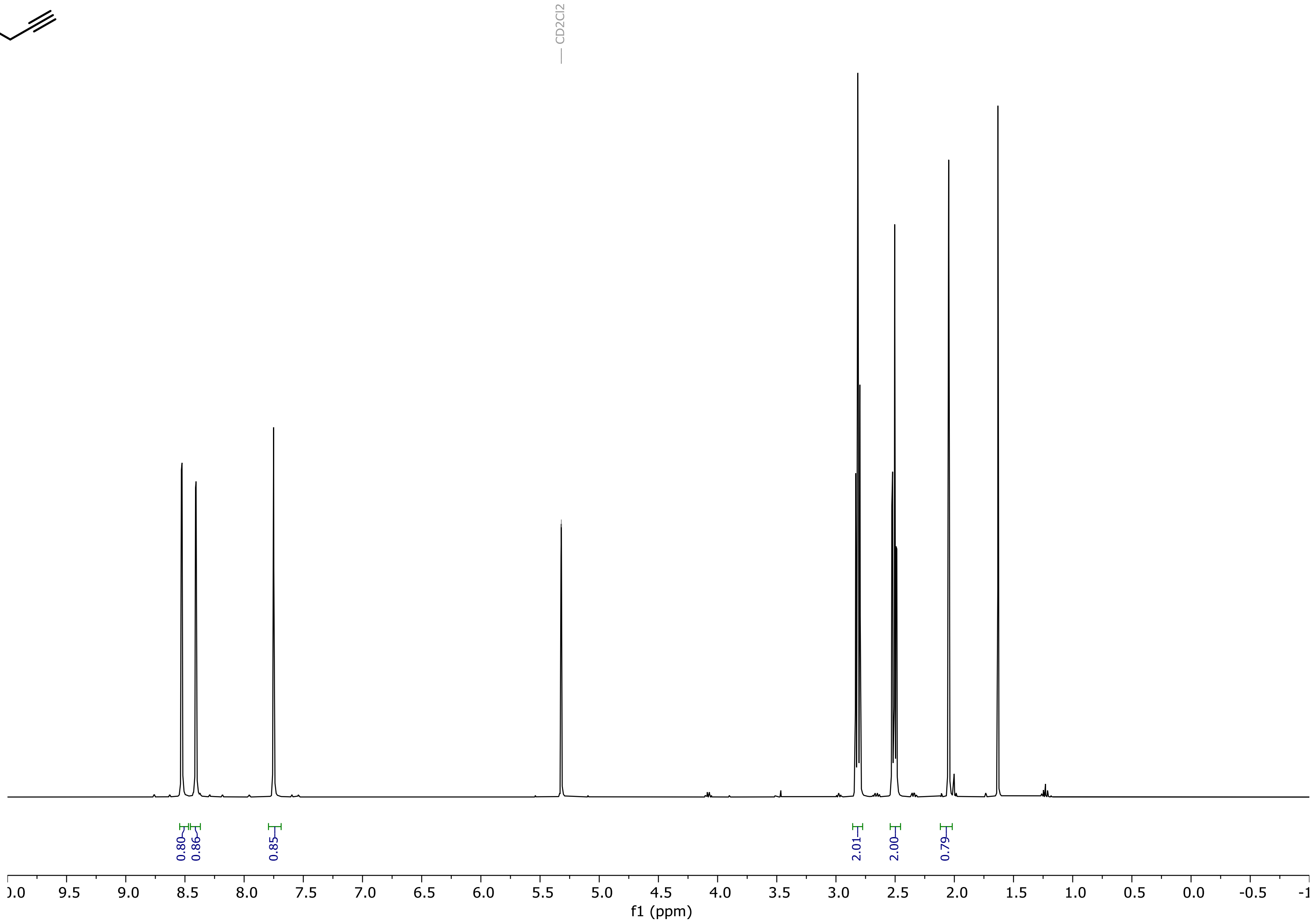

^{13}C NMR spectrum of S2 (101 MHz, CD_2Cl_2)

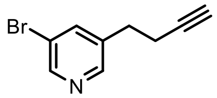

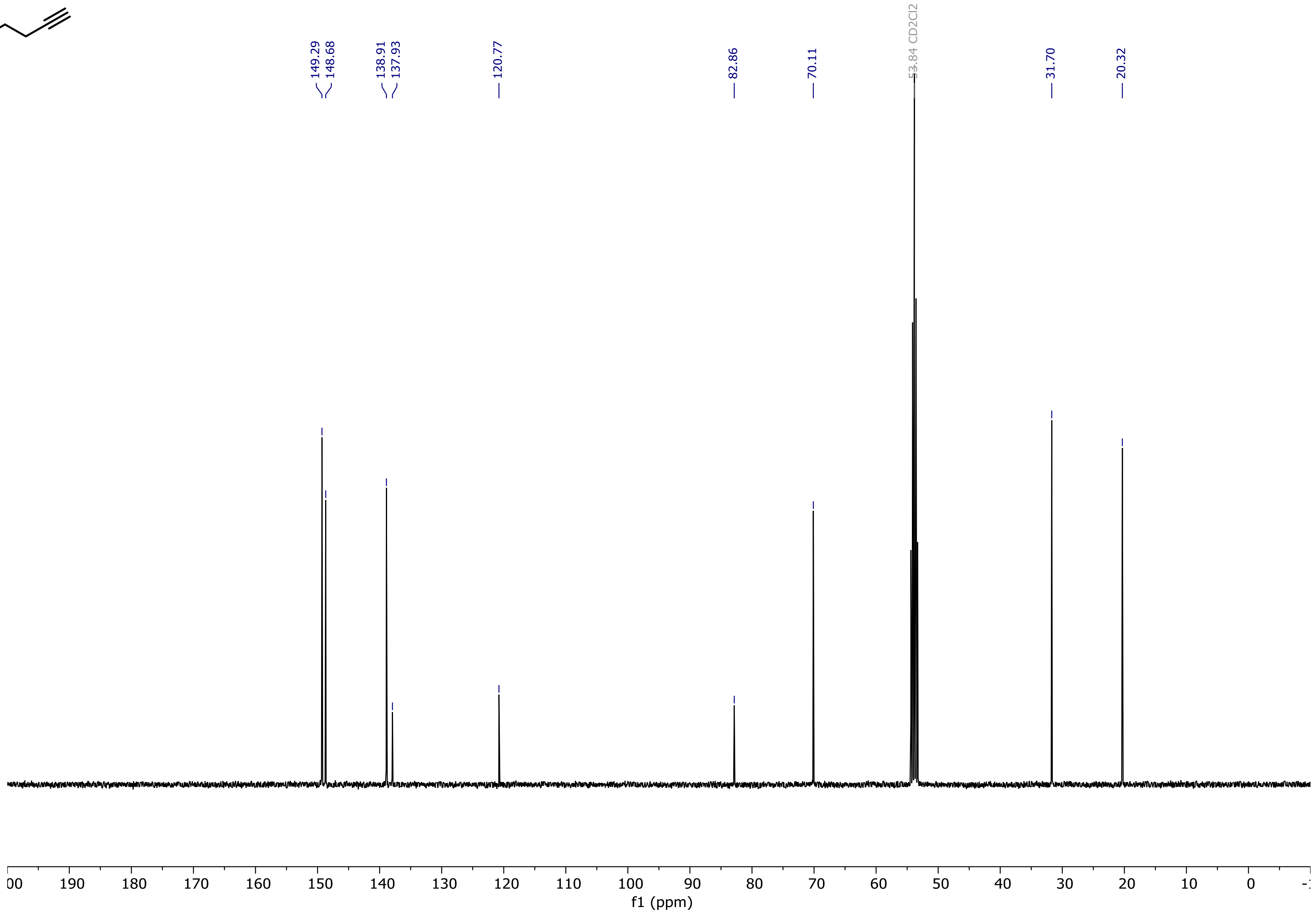

^{1}H NMR spectrum of 10 (400 MHz, CD_2Cl_2)

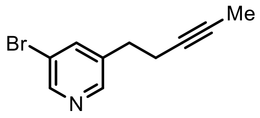

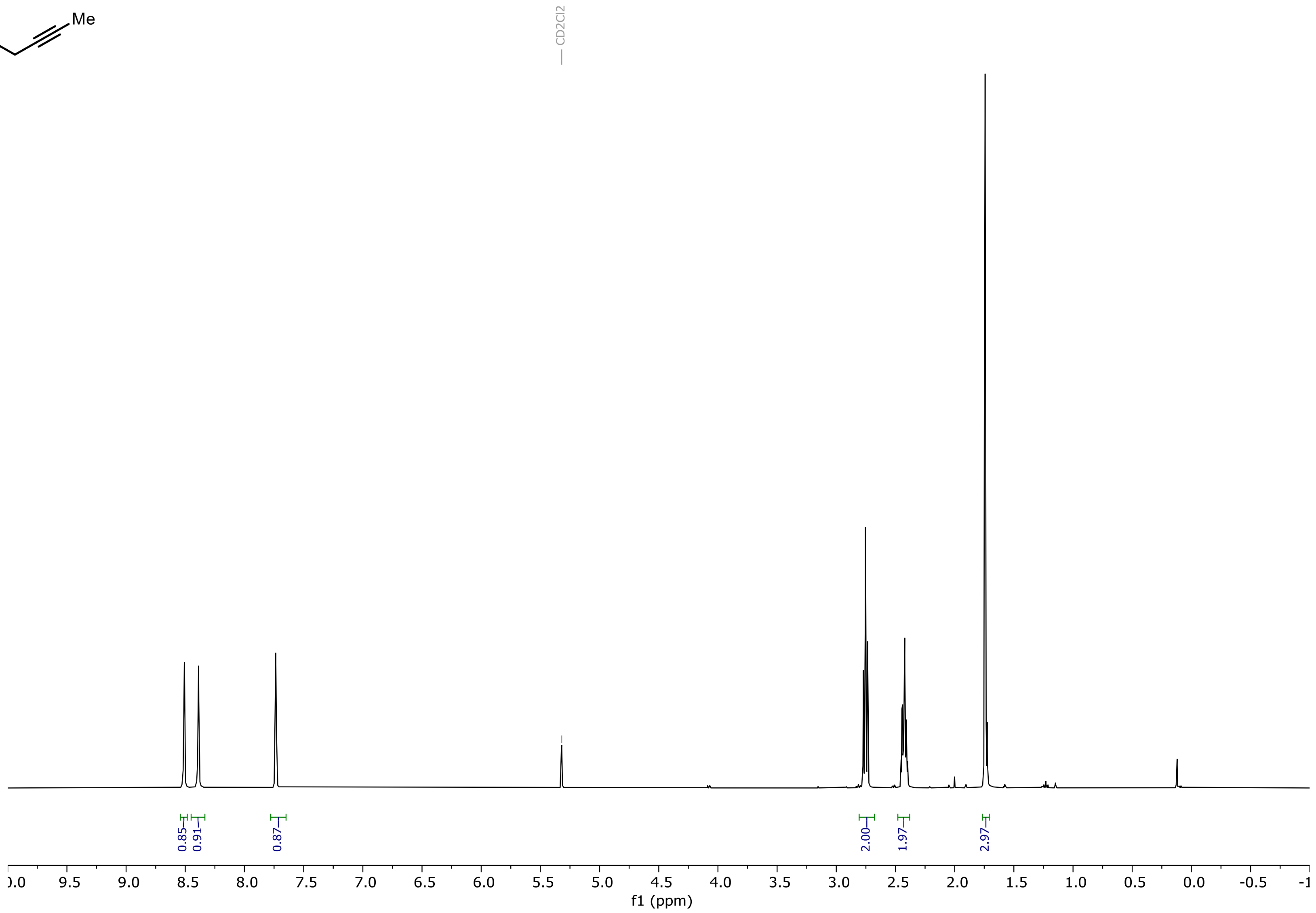

^{13}C NMR spectrum of 10 (101 MHz, CD_2Cl_2)

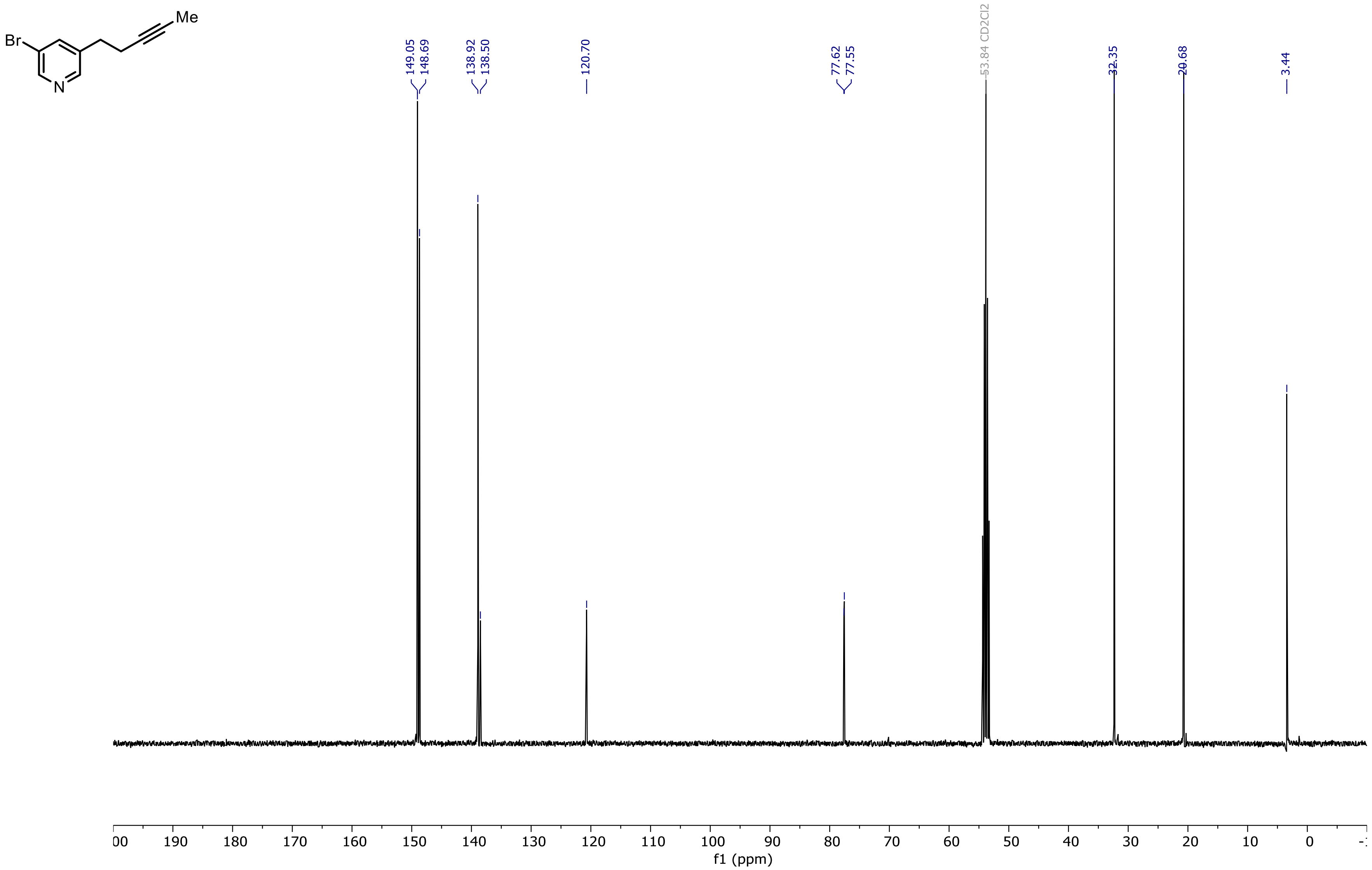

^{1}H NMR spectrum of 18 (400 MHz, CDCl$_3$)

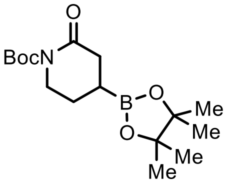

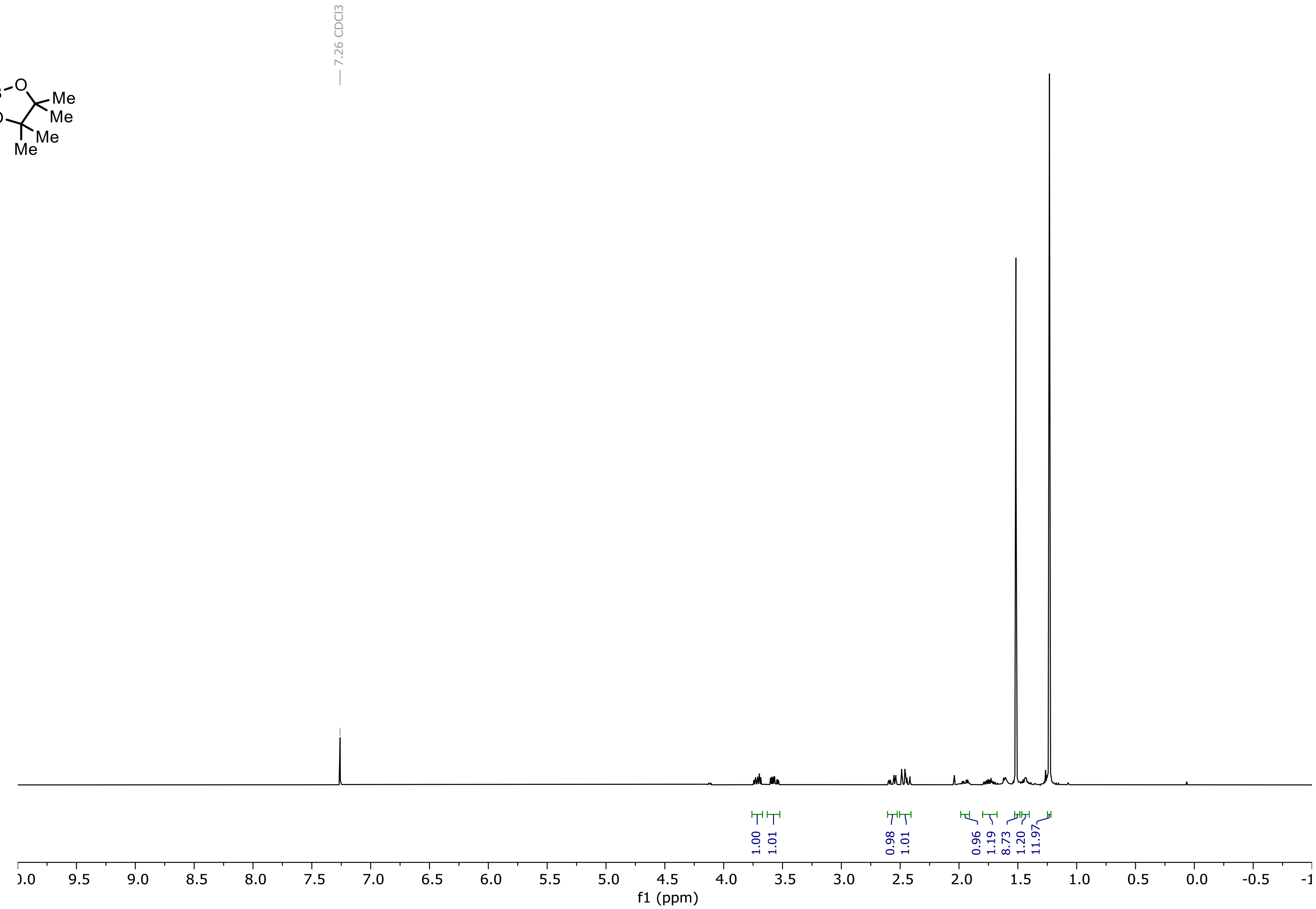

^{13}C NMR spectrum of 18 (101 MHz, $CDCl_3$)

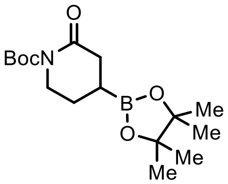

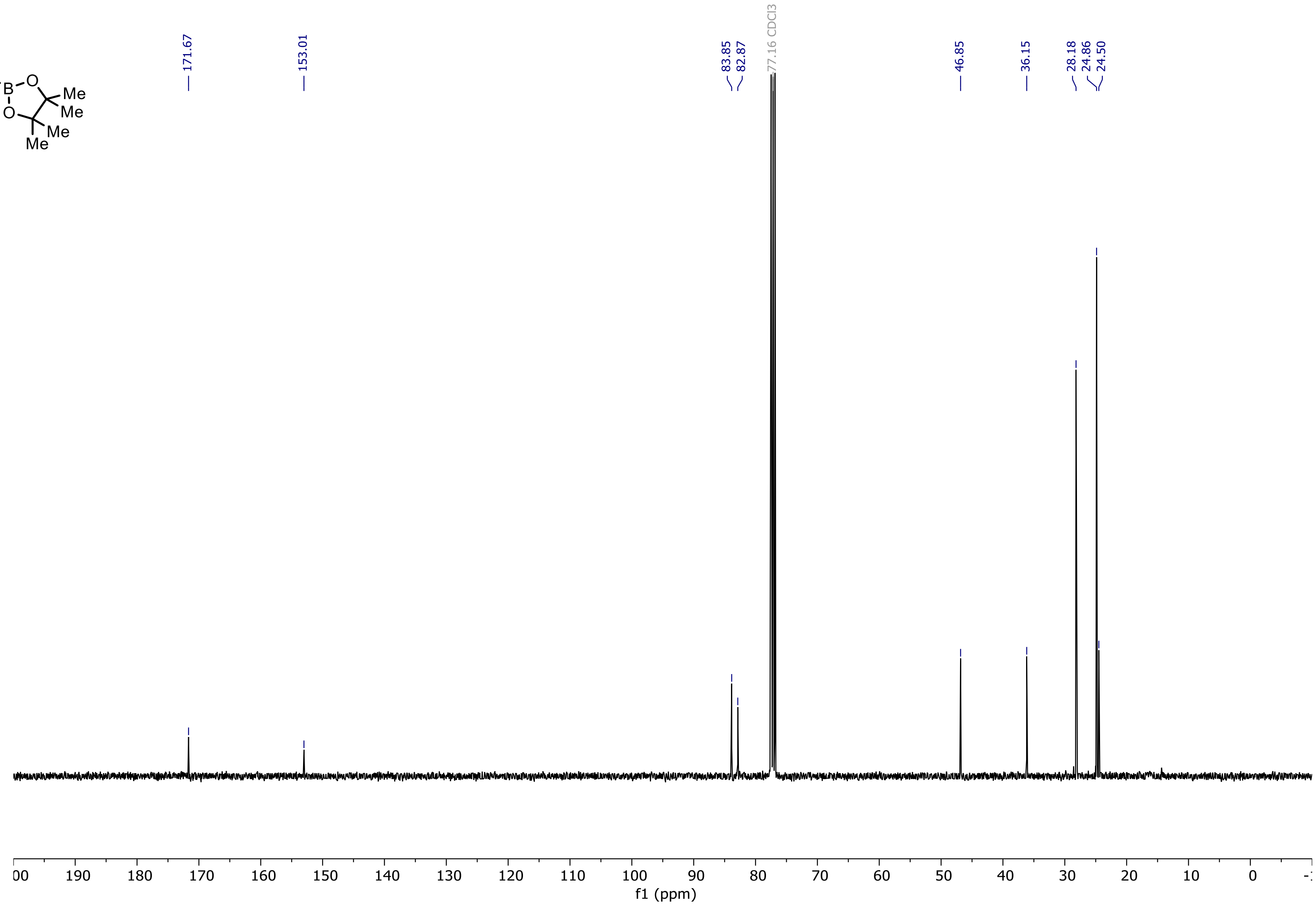

^{1}H NMR spectrum of 19 (400 MHz, CD_3CN)

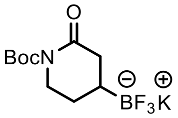

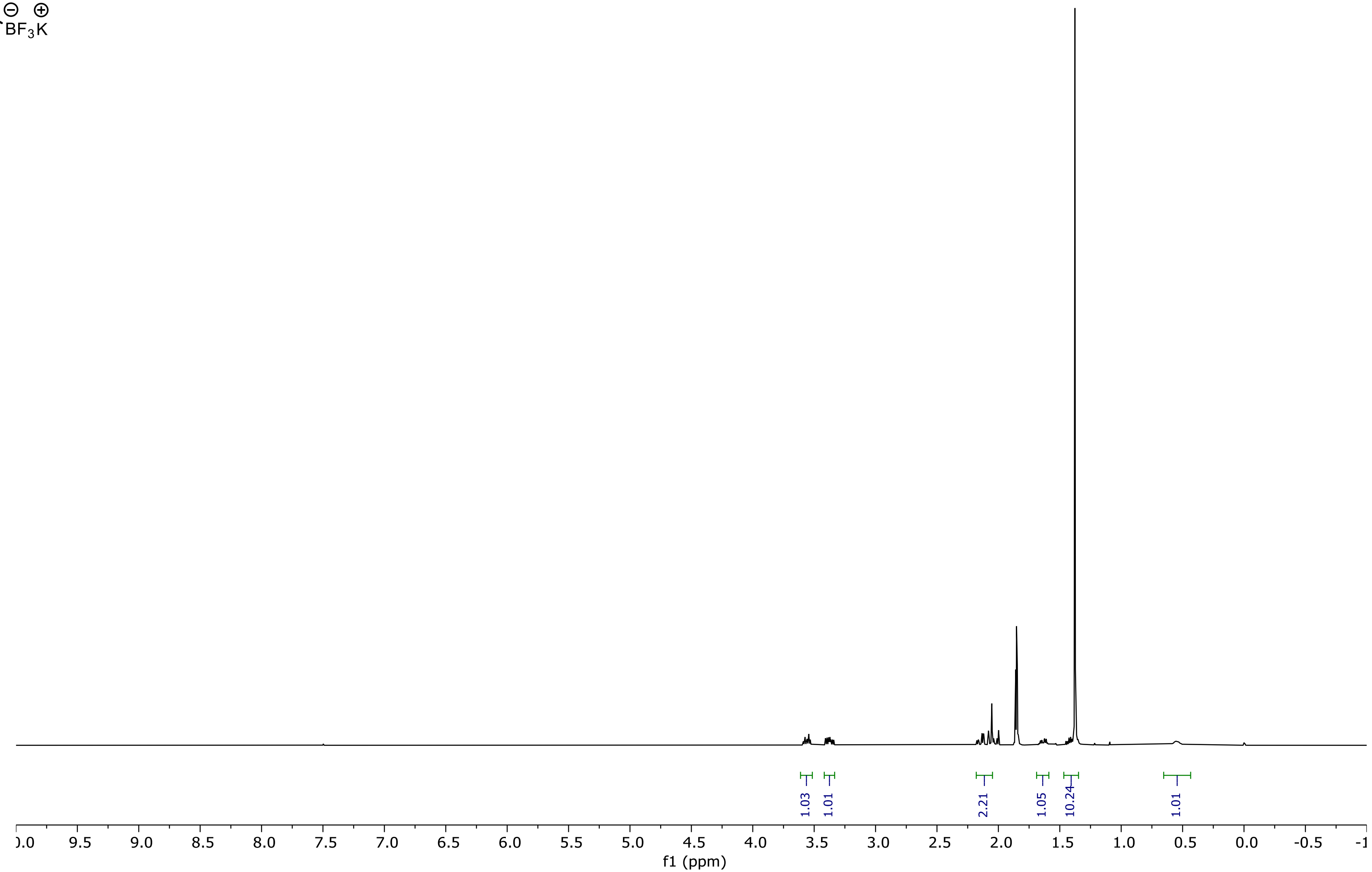

^{13}C NMR spectrum of 19 (101 MHz, CD_3CN)

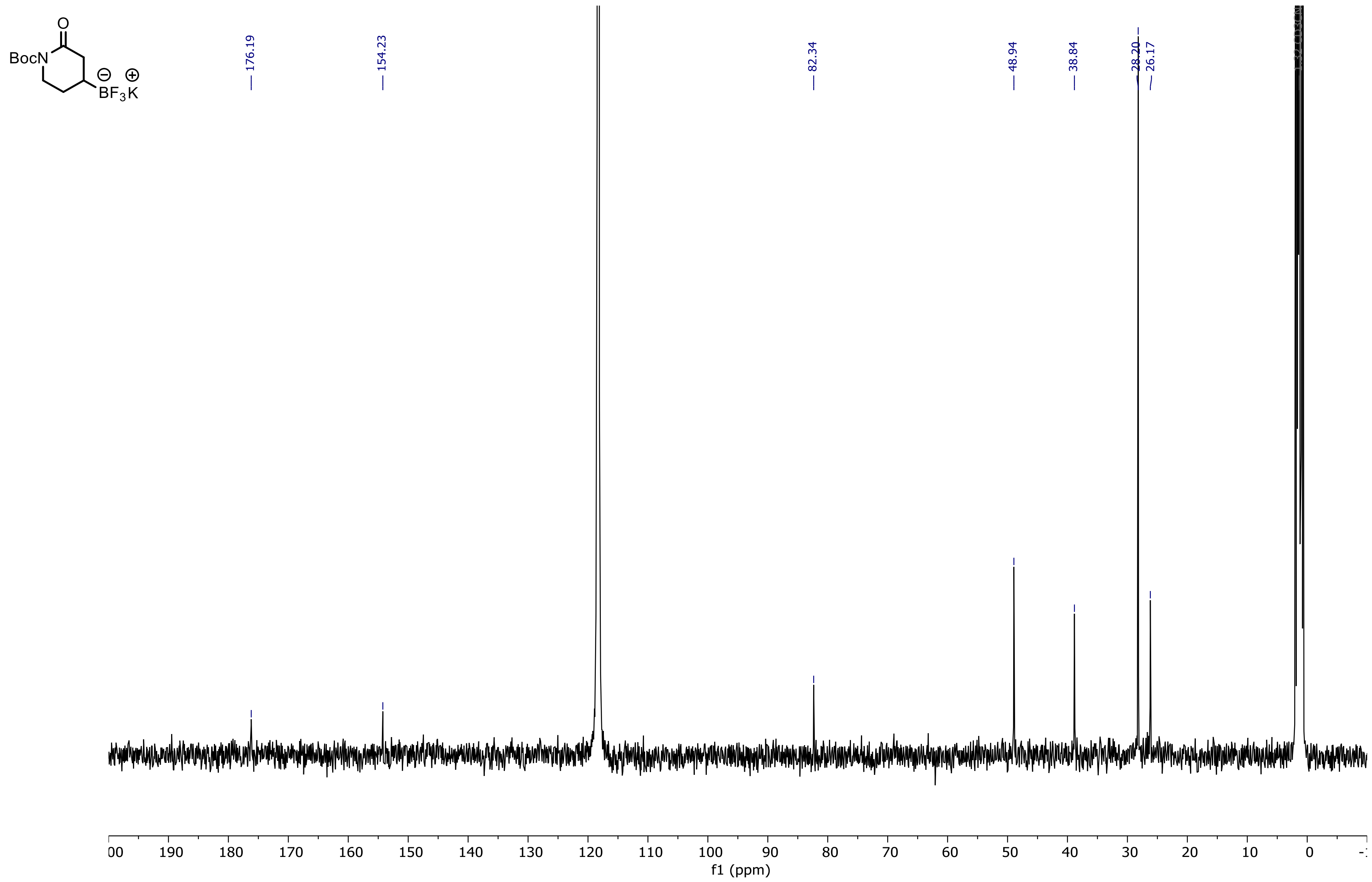

^{1}H NMR spectrum of 21 (400 MHz, CD_2Cl_2)

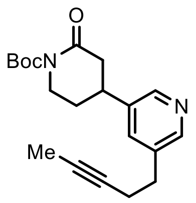

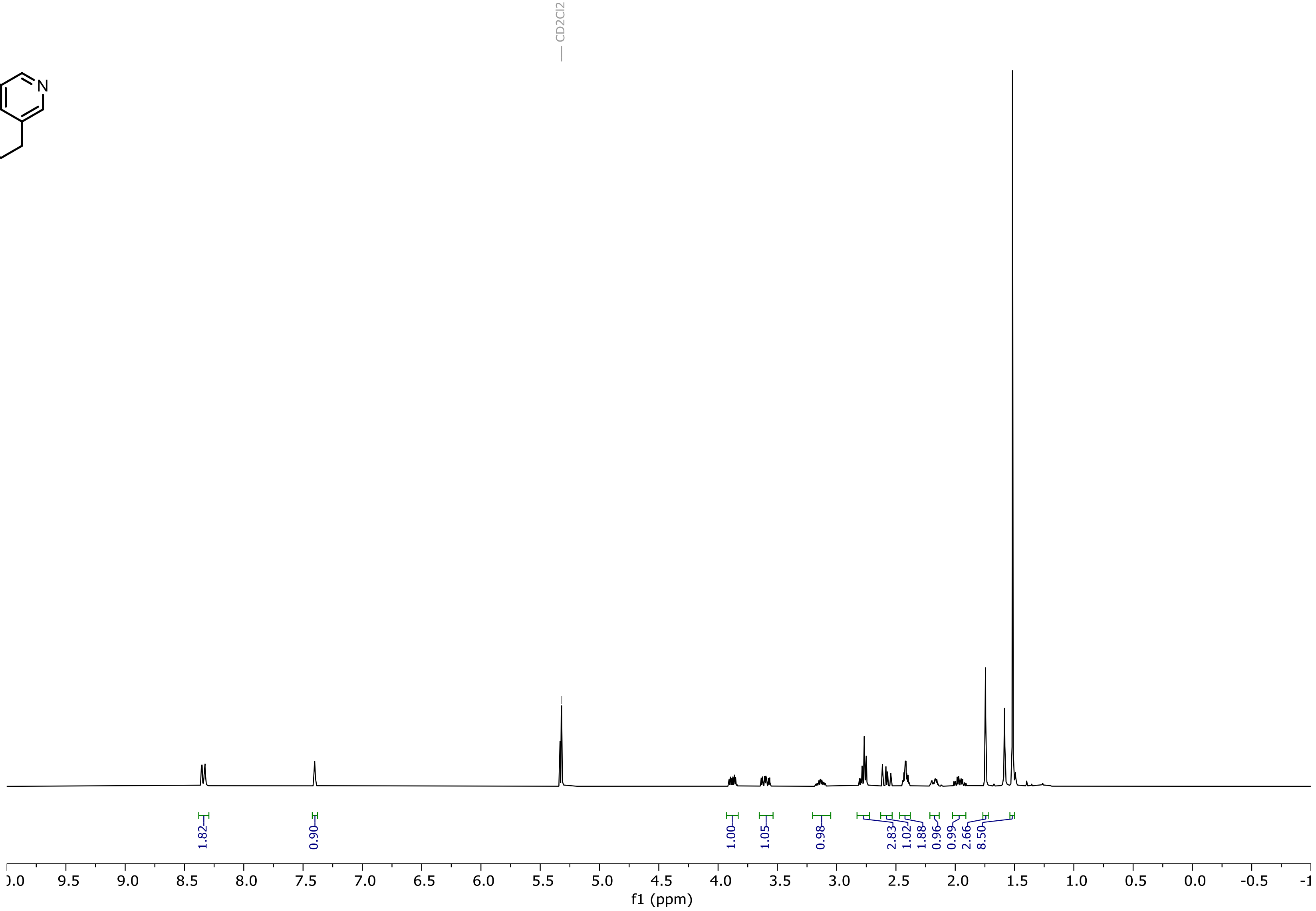

^{13}C NMR spectrum of 21 (101 MHz, CD_2Cl_2)

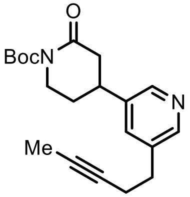

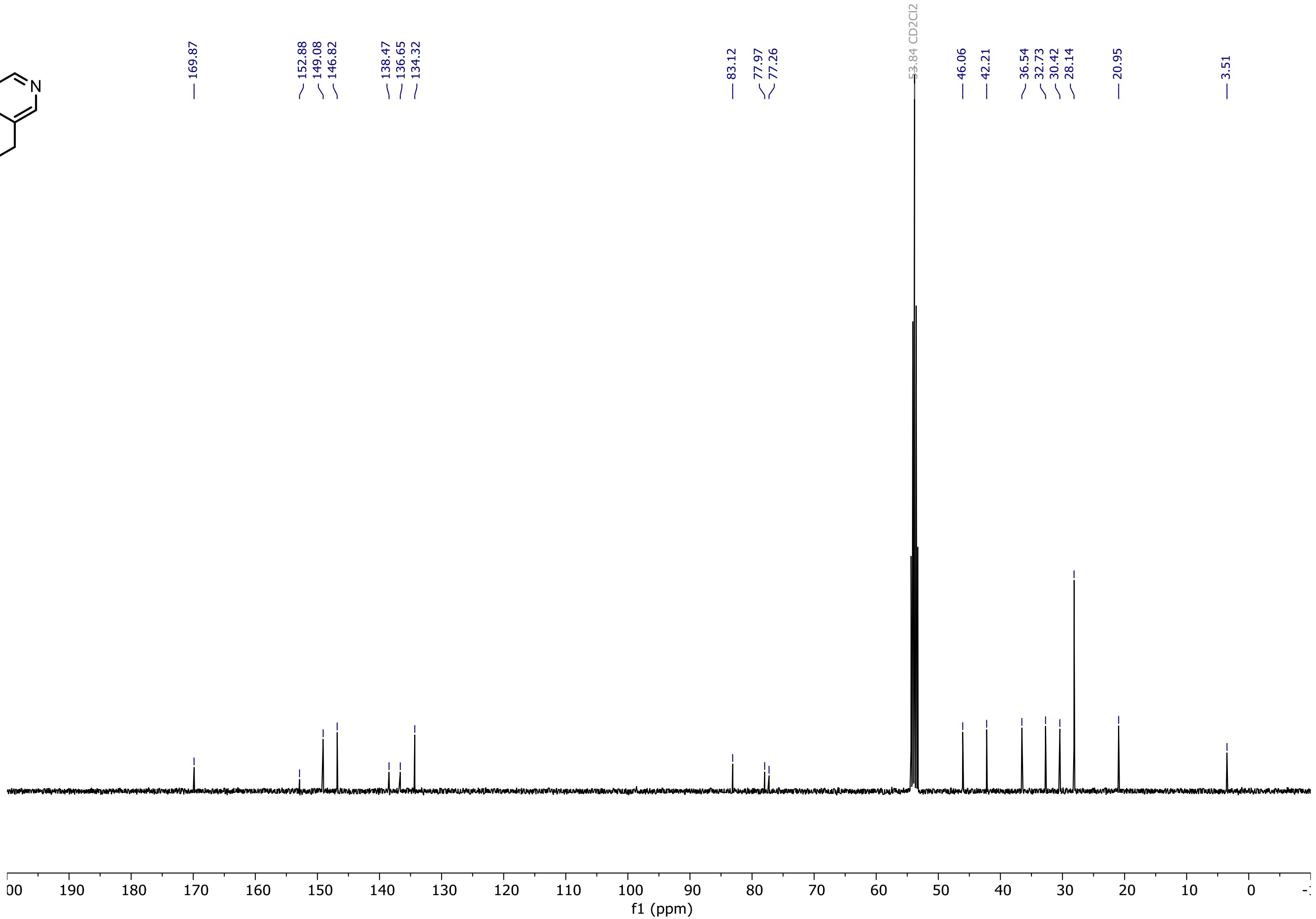

^{1}H NMR spectrum of S5 (400 MHz, CD_2Cl_2)

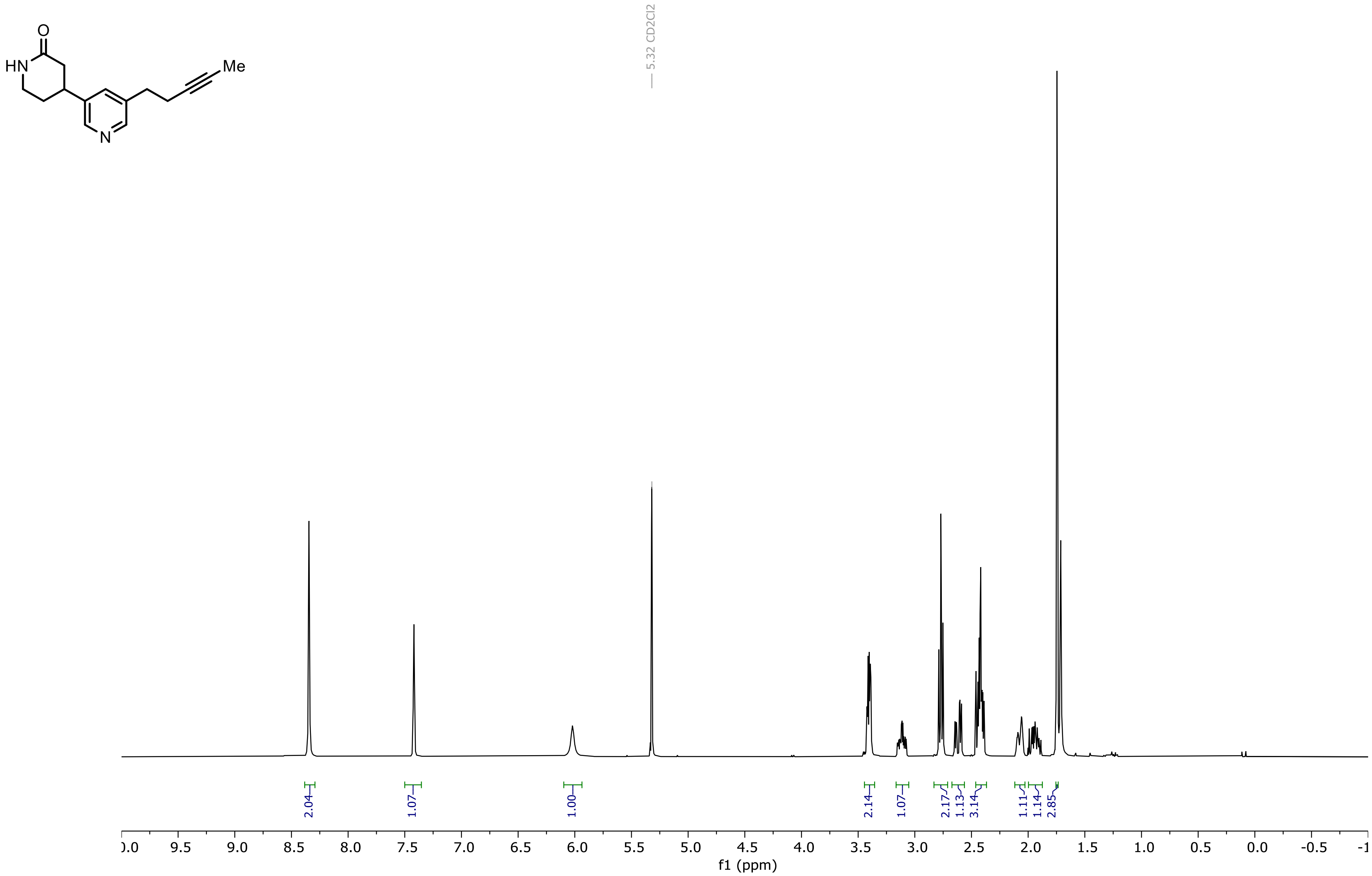

^{13}C NMR spectrum of S5 (101 MHz, CD_2Cl_2)

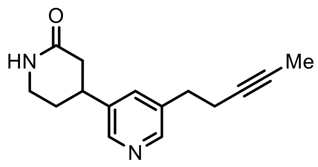

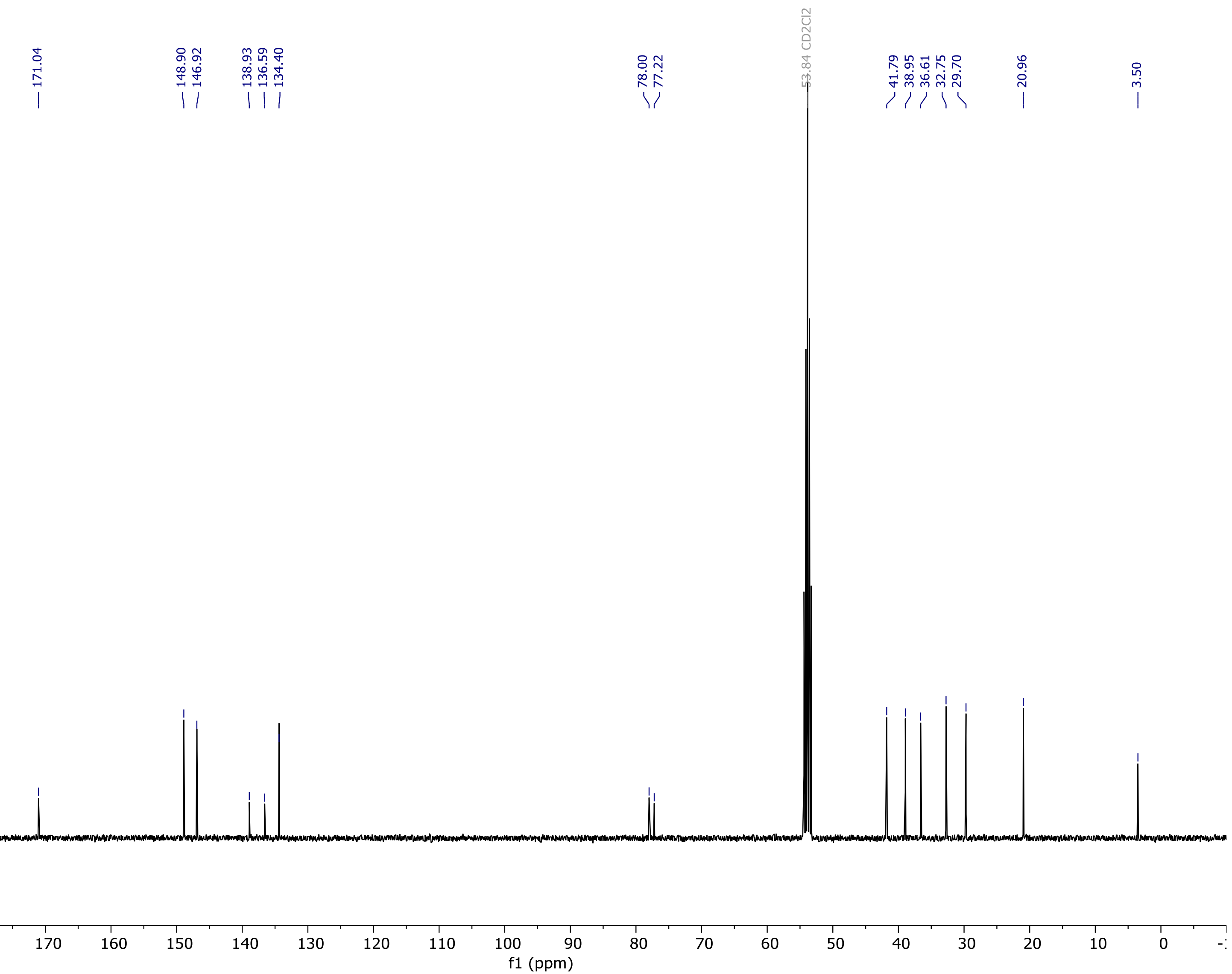

^{1}H NMR spectrum of 23 (400 MHz, CD_2Cl_2)

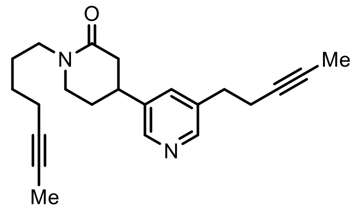

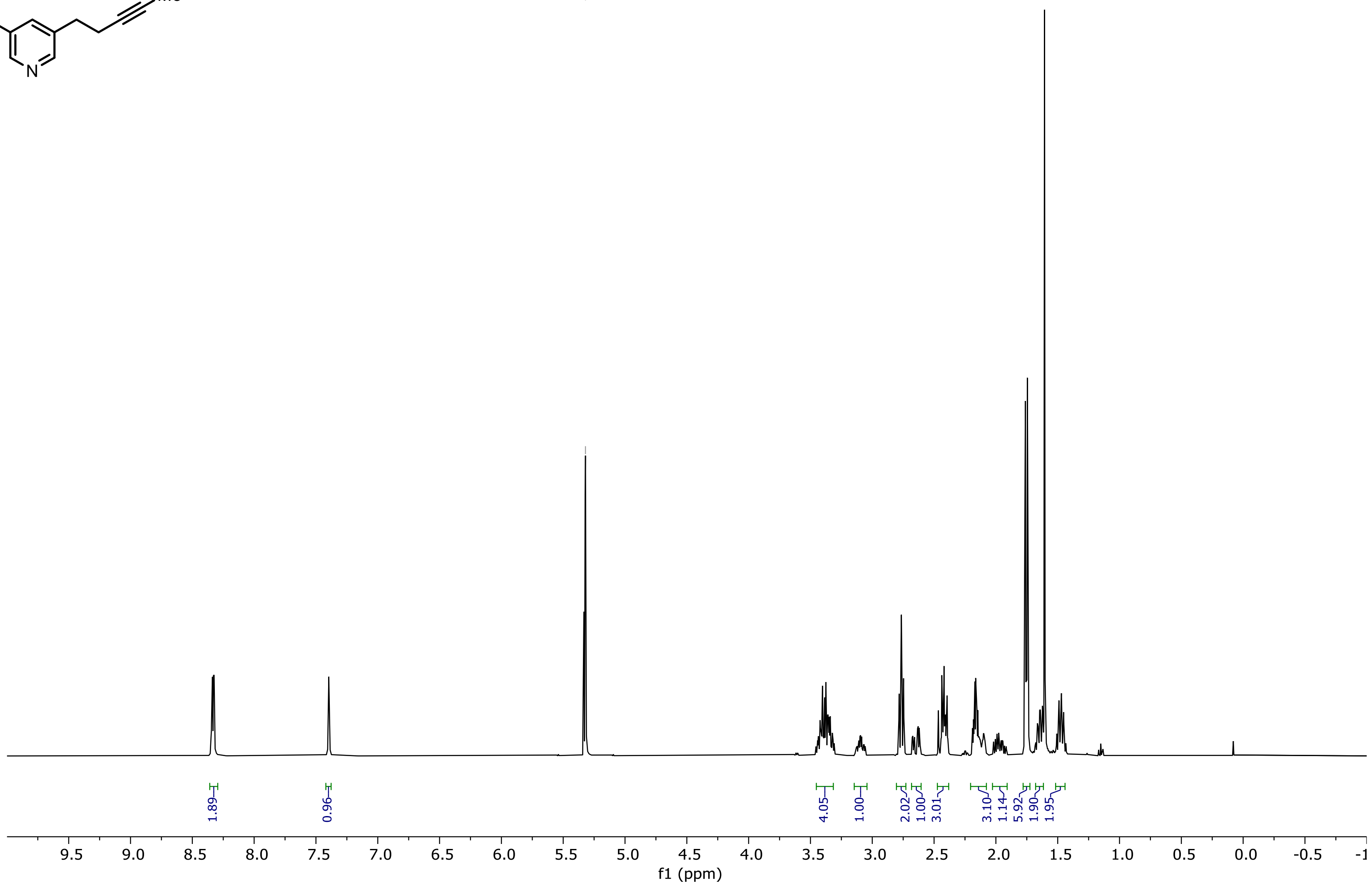

^{13}C NMR spectrum of 23 (101 MHz, CD_2Cl_2)

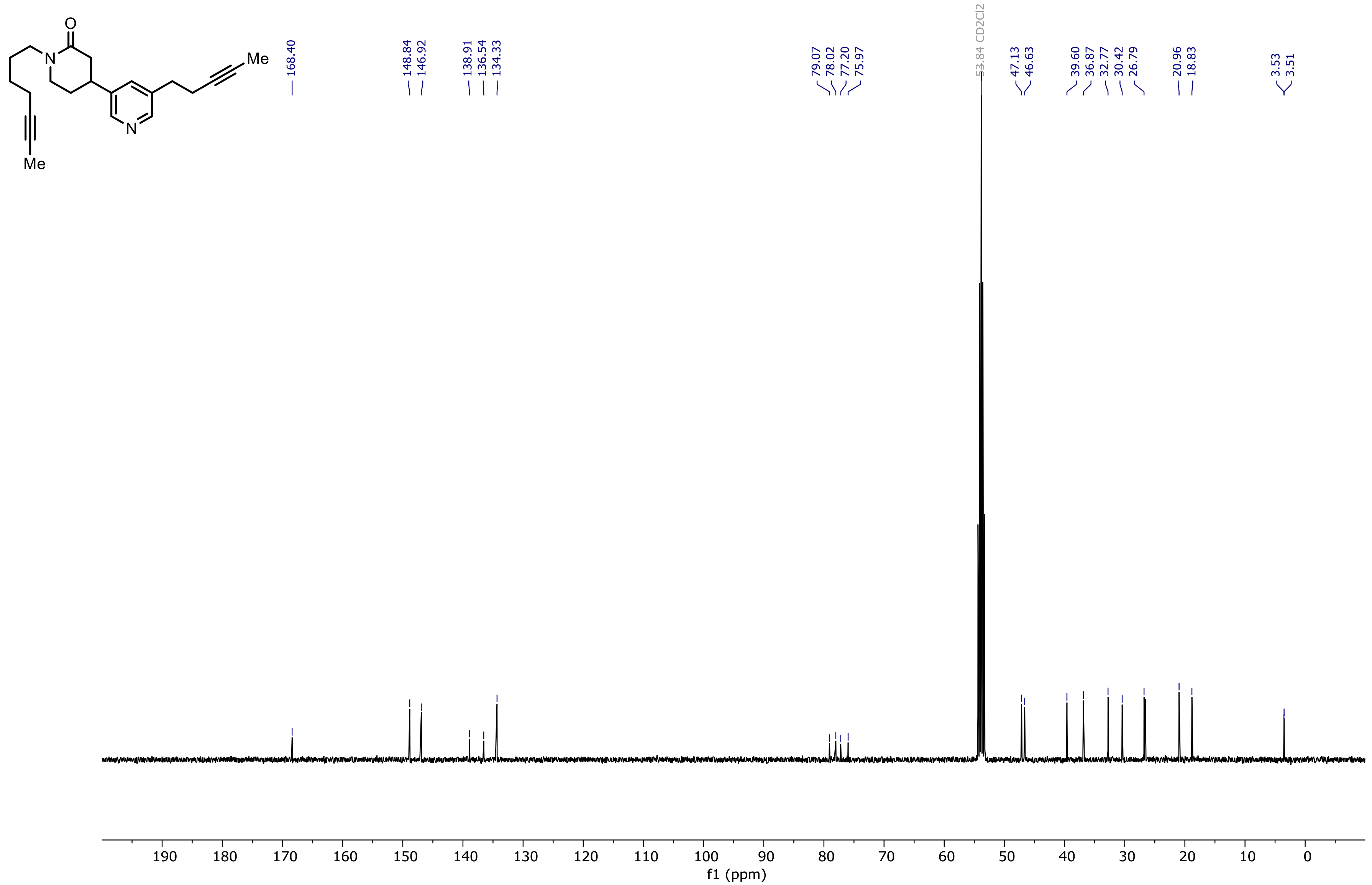

^{1}H NMR spectrum of 24 (400 MHz, CD_2Cl_2)

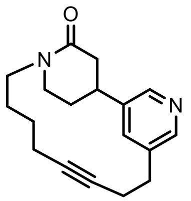

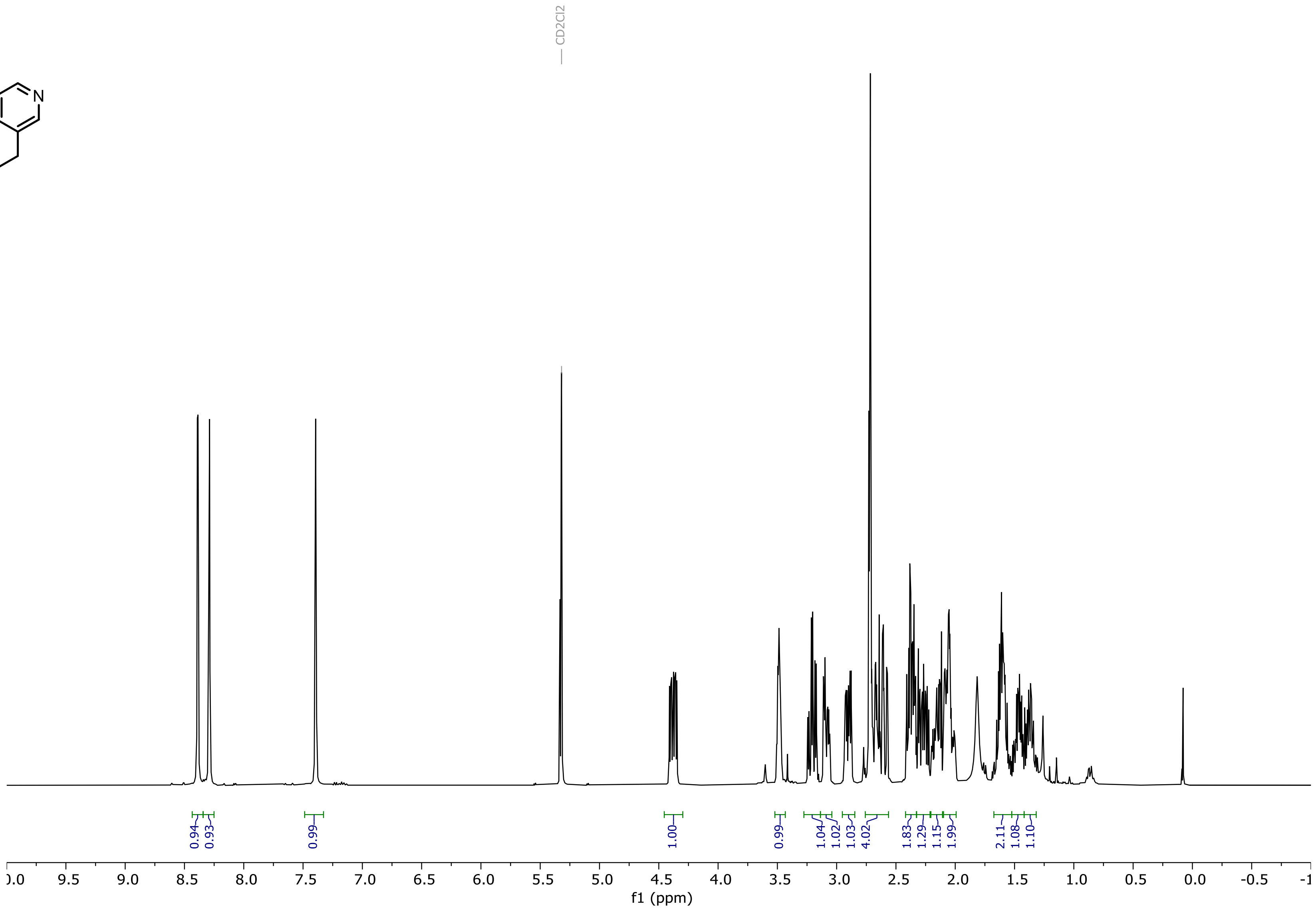

^{13}C NMR spectrum of 24 (101 MHz, CD_2Cl_2)

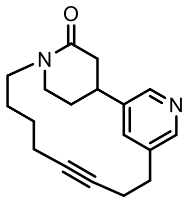

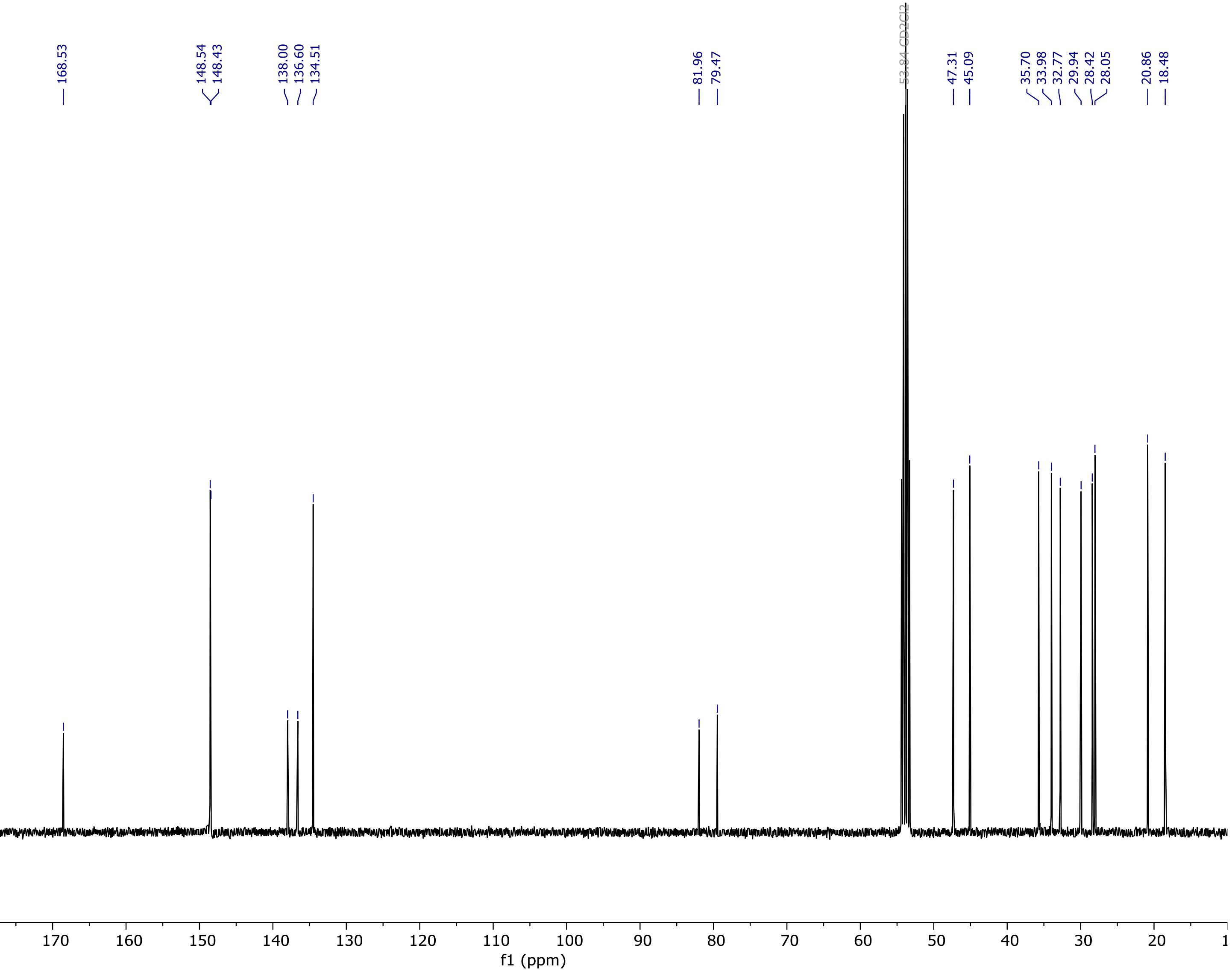

^{1}H NMR spectrum of 25 (600 MHz, CD_2Cl_2)

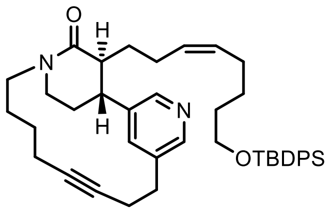

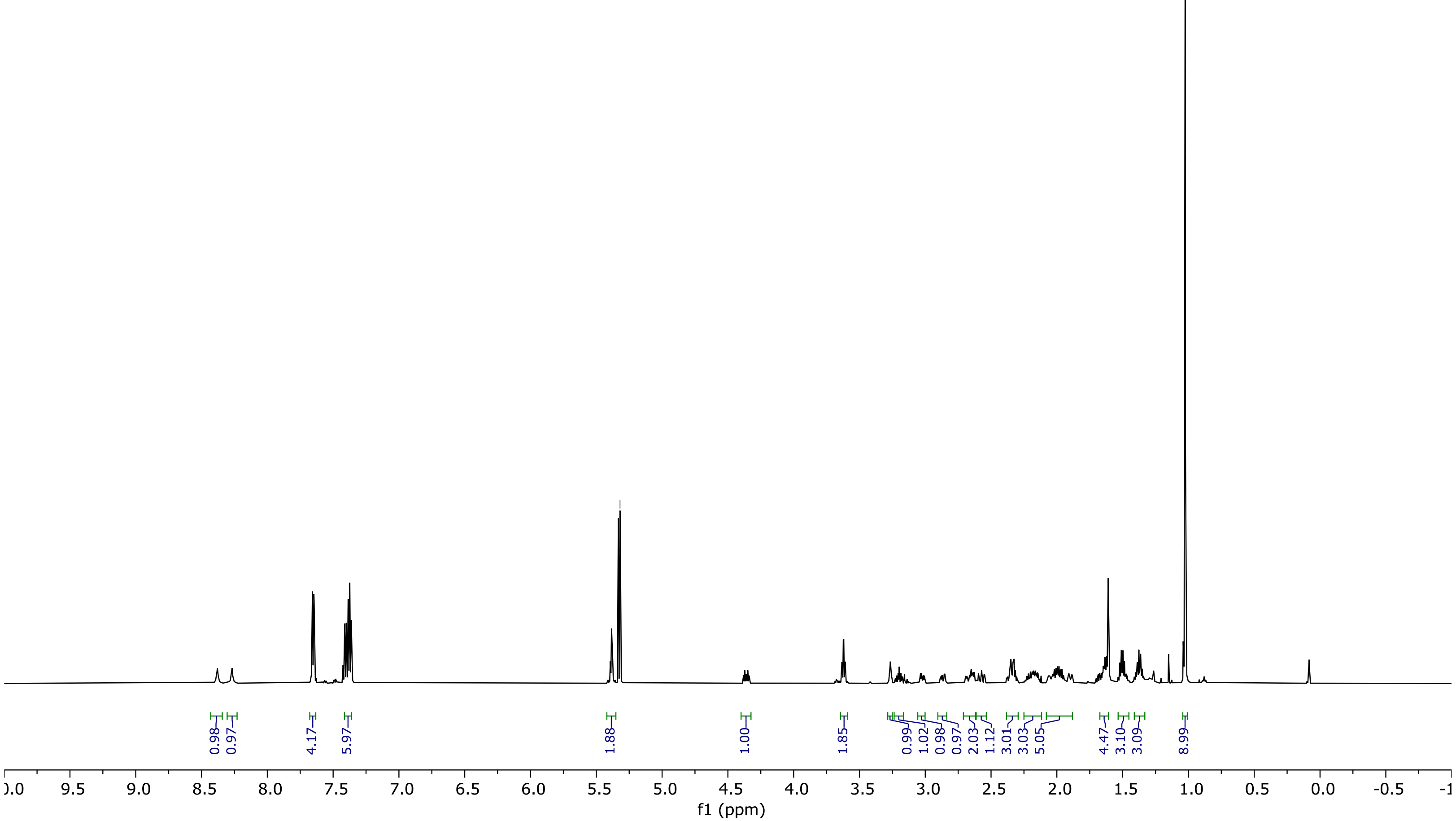

^{13}C NMR spectrum of 25 (151 MHz, CD_2Cl_2)

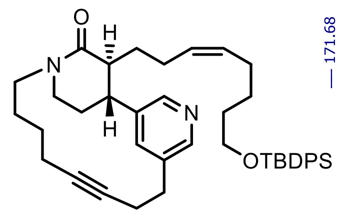

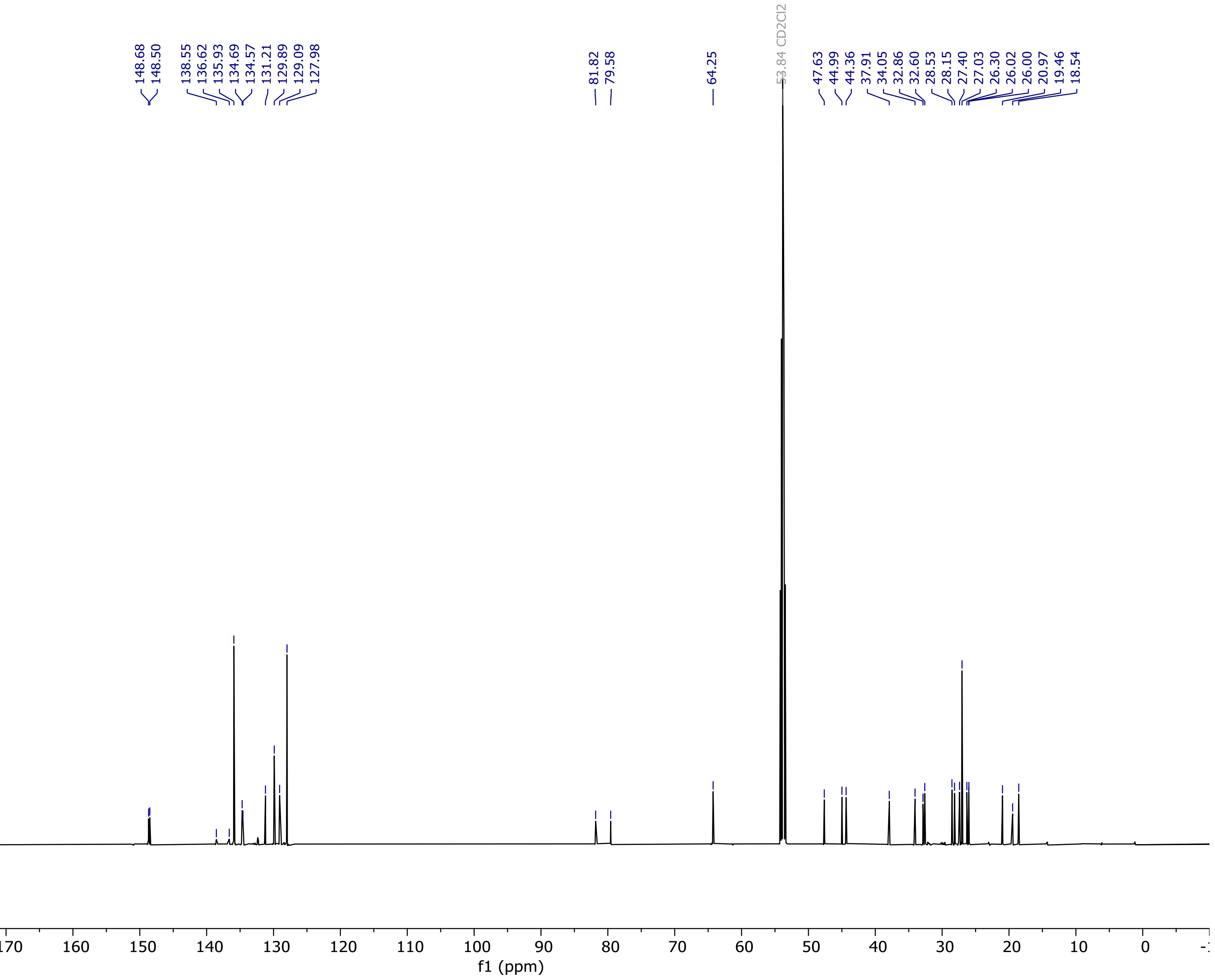

HSQC spectrum of 25

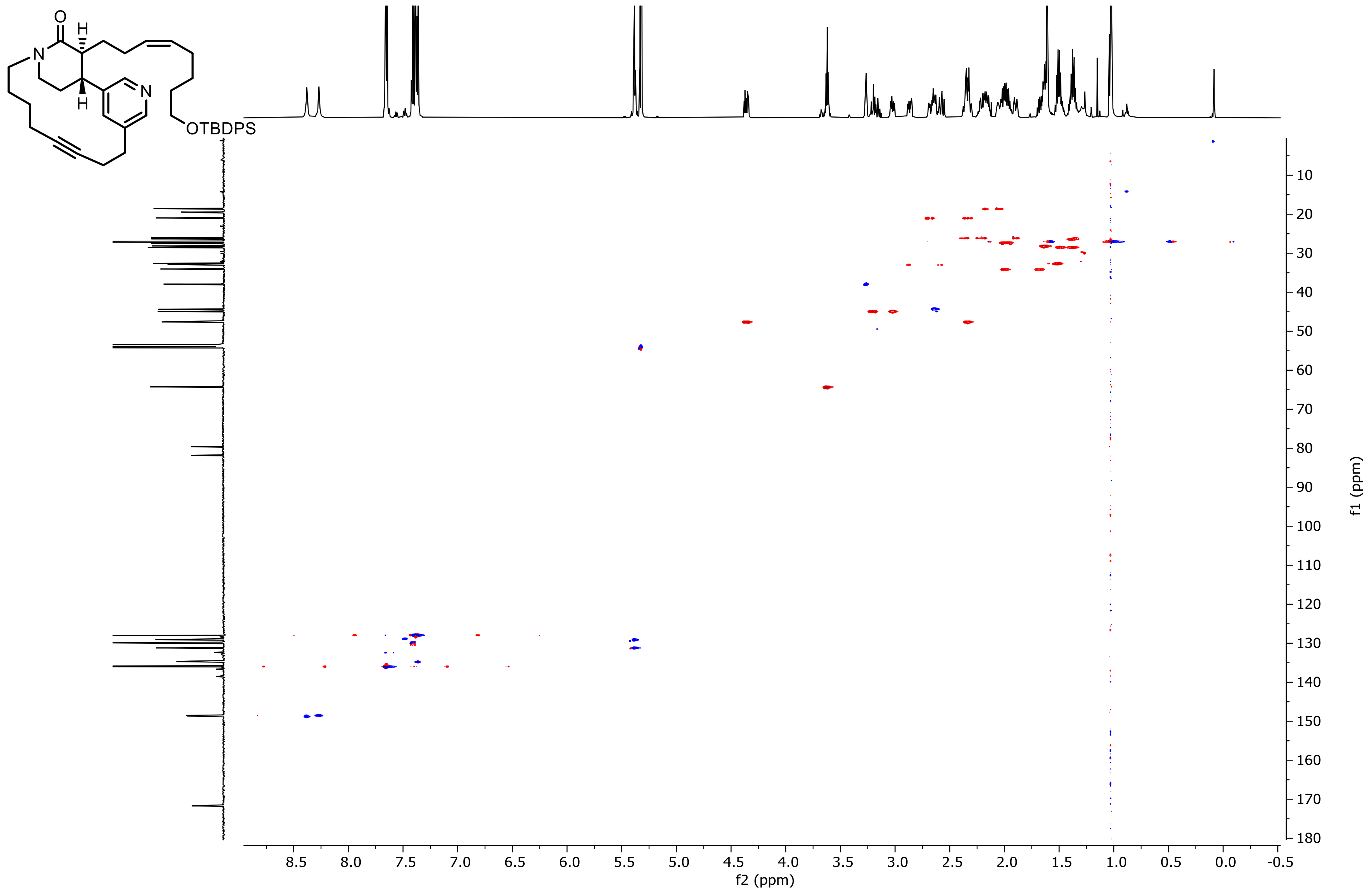

^{1}H-^{1}H COSY spectrum of 25

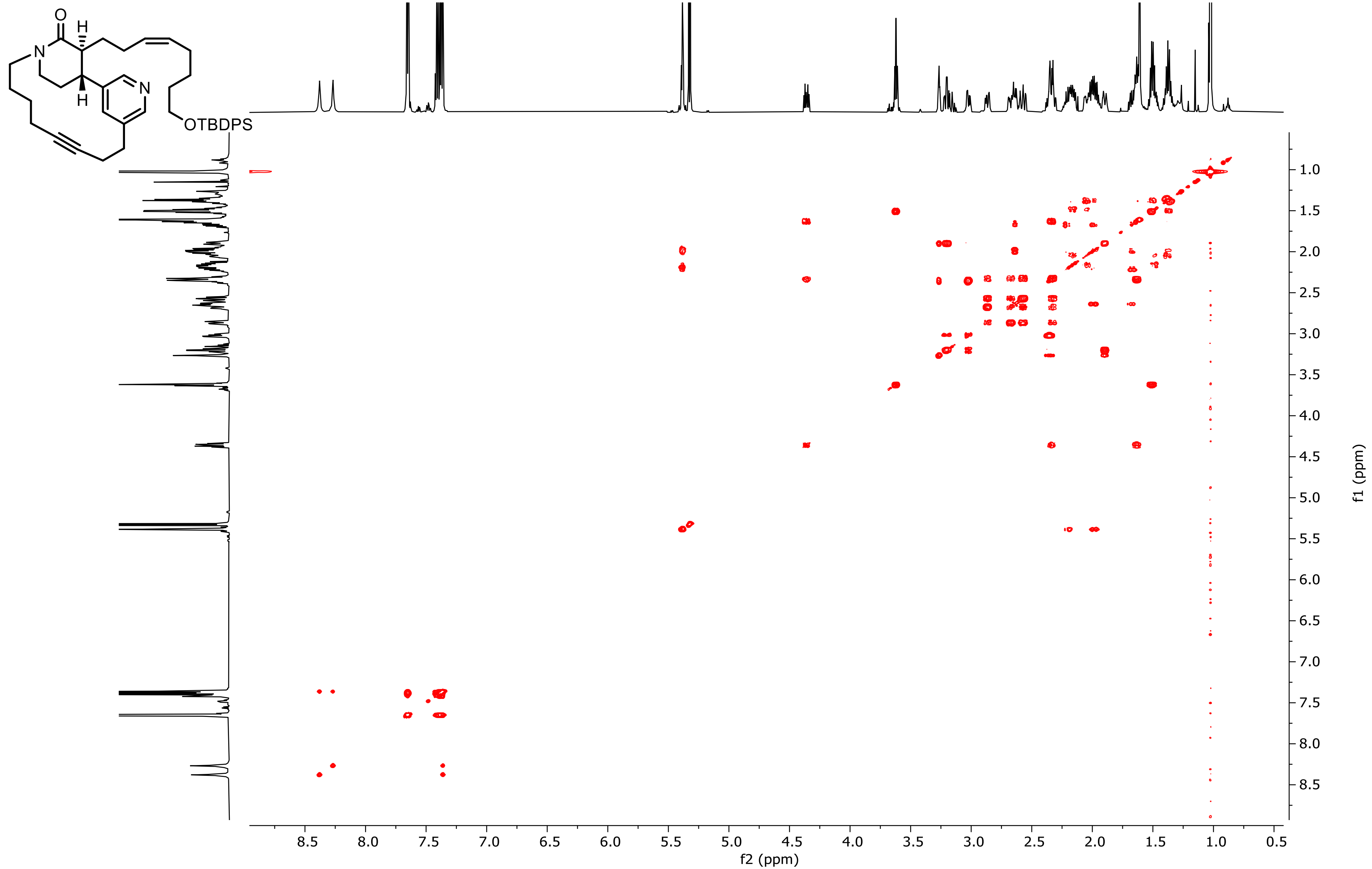

HMBC spectrum of 25

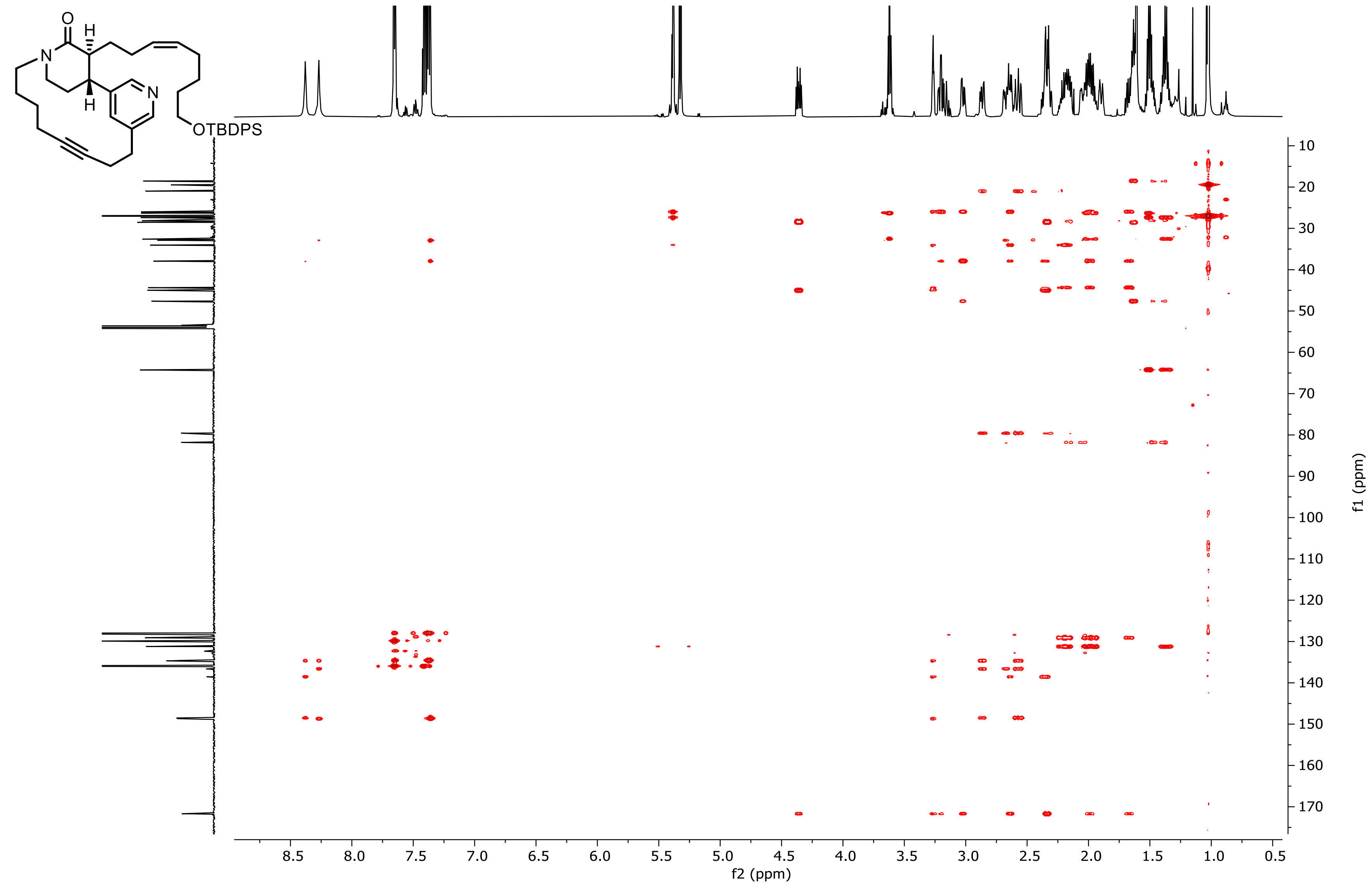

NOESY spectrum of 25

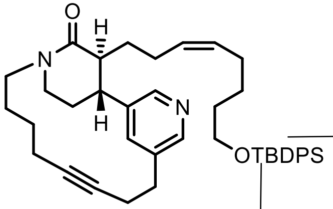

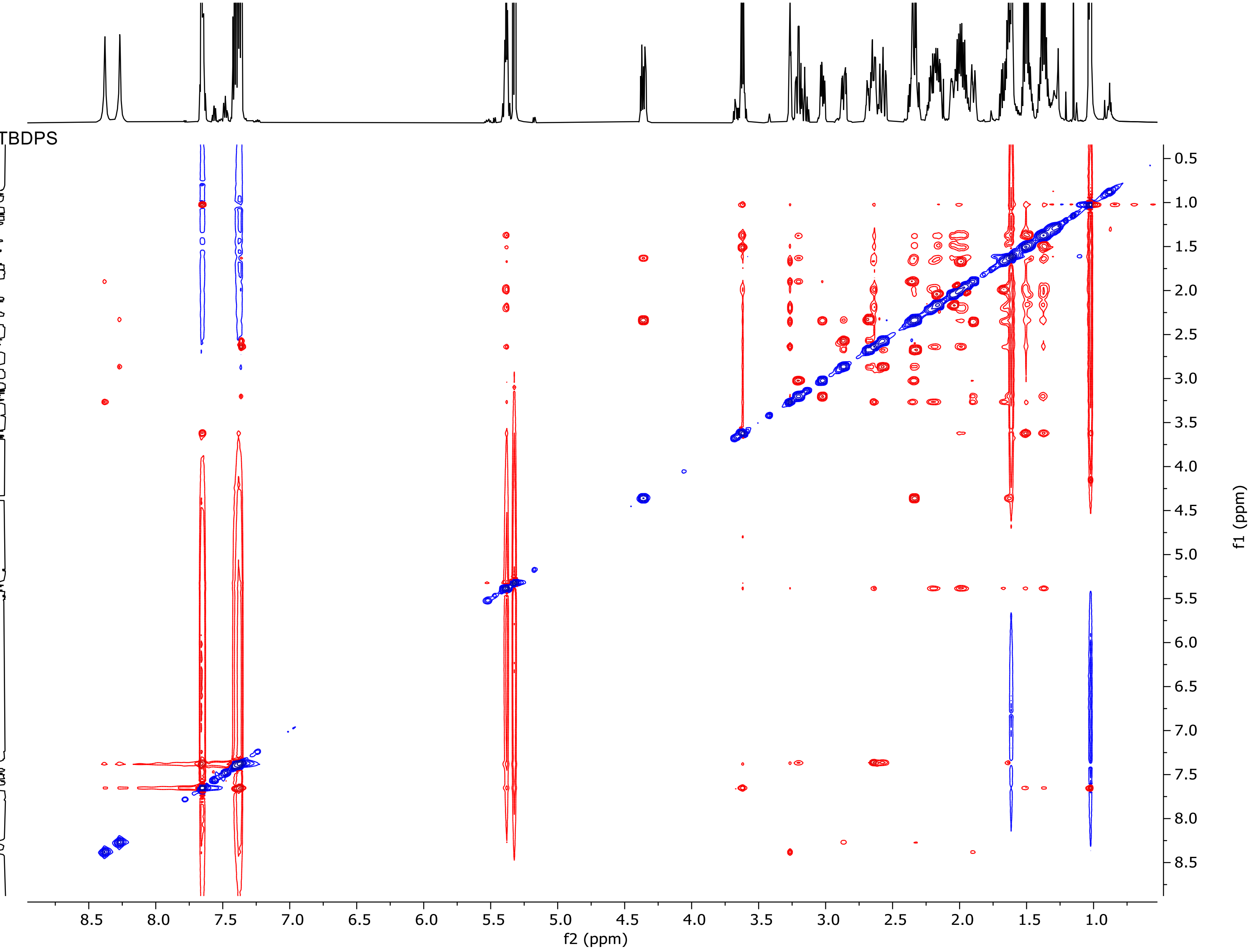

^{1}H NMR spectrum of 26 (400 MHz, CD_2Cl_2)

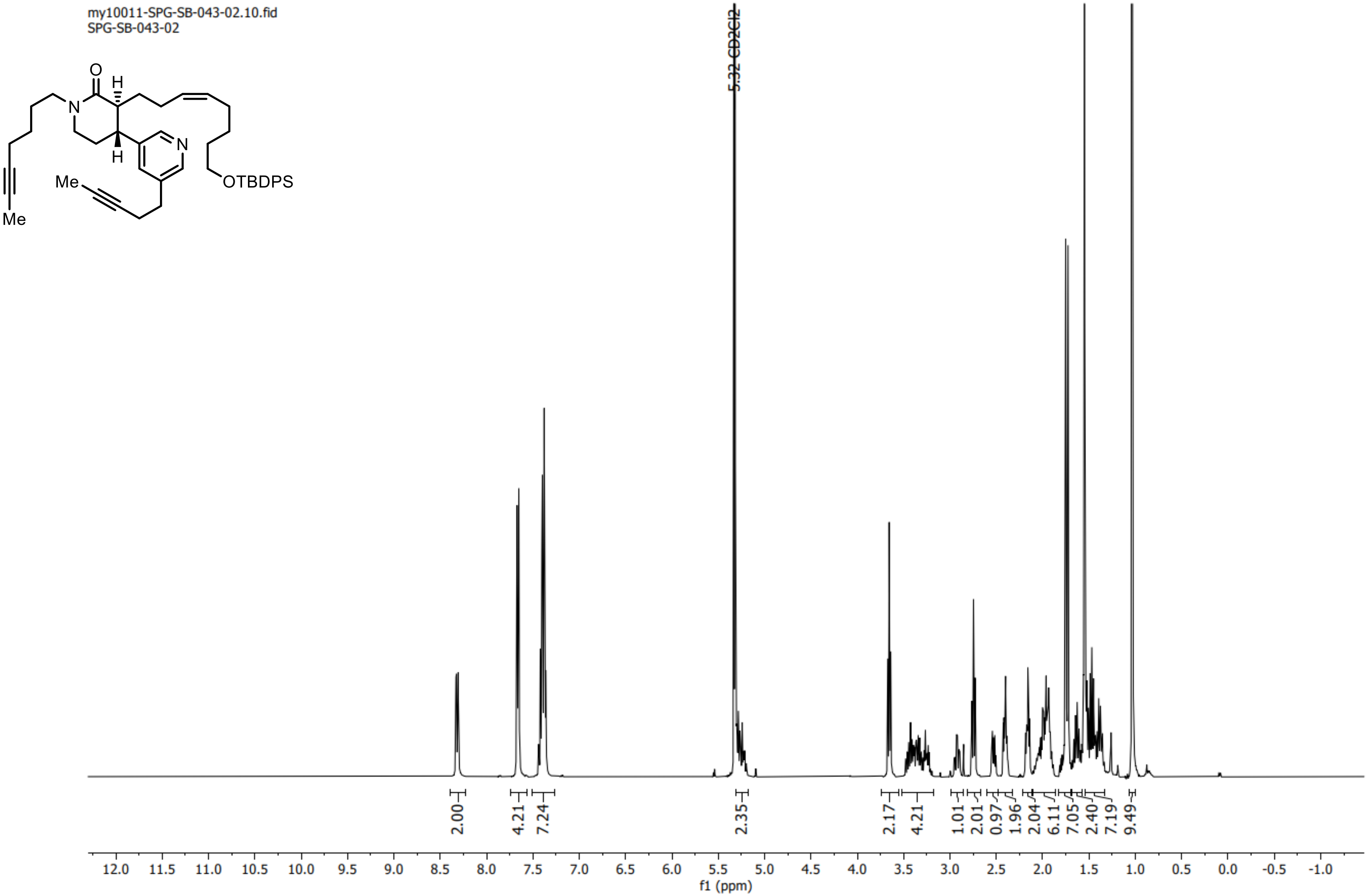

^{13}C NMR spectrum of 26 (101 MHz, CD_2Cl_2)

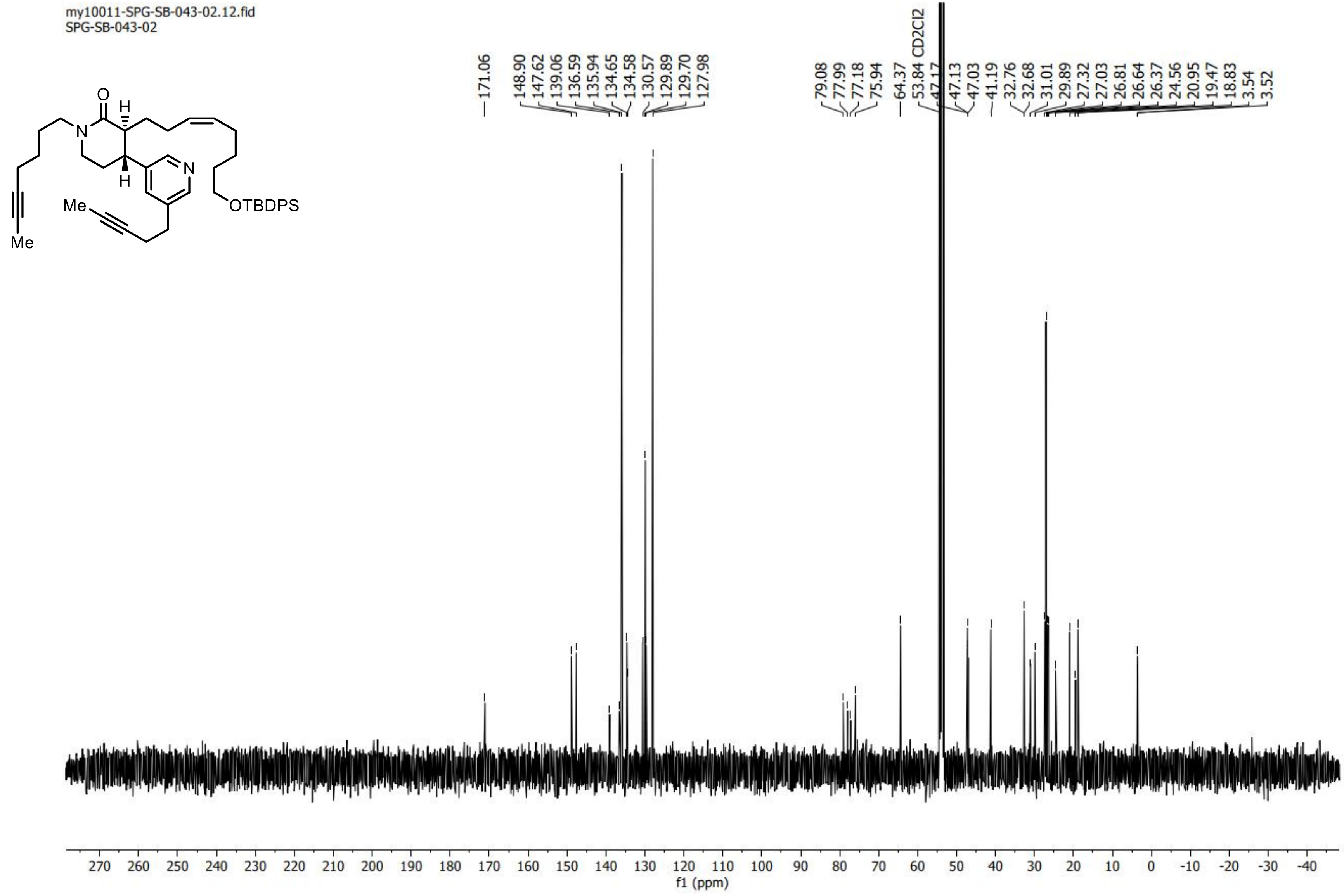

^{1}H NMR spectrum of 28 (400 MHz, CDCl$_3$)

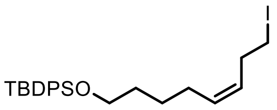

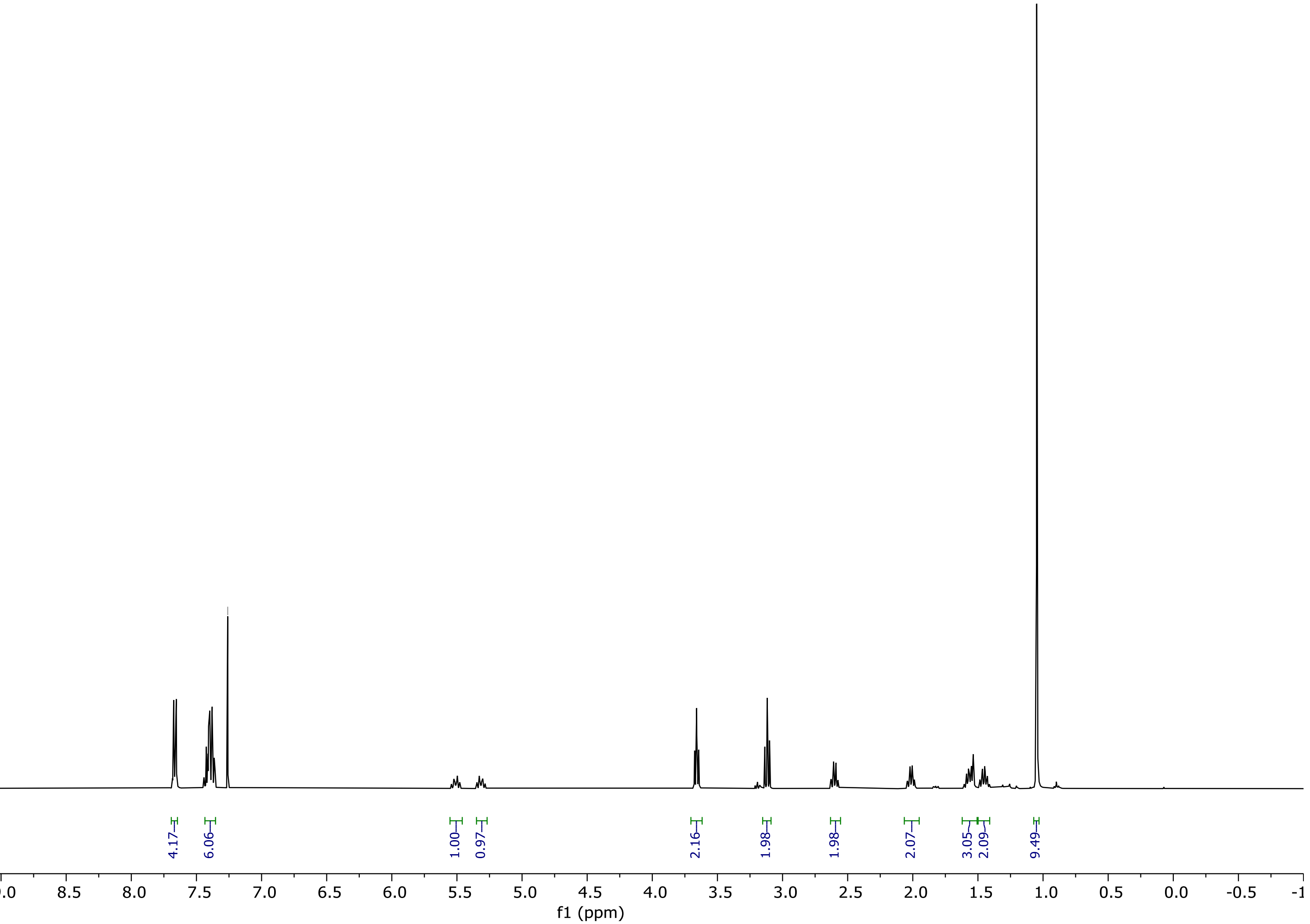

^{13}C NMR spectrum of 28 (101 MHz, $CDCl_3$)

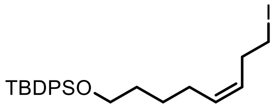

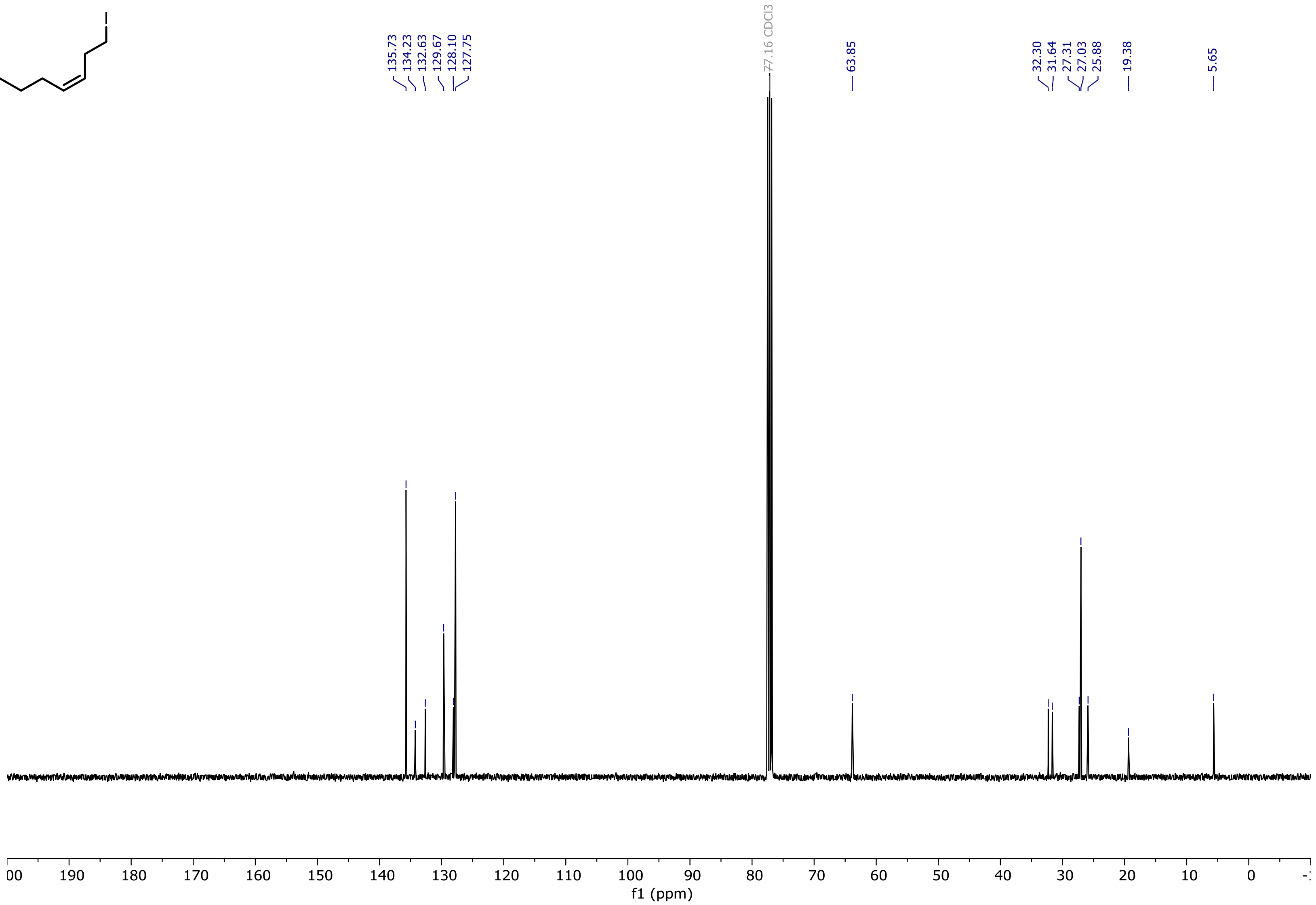

^{1}H NMR spectrum of 30 (600 MHz, CD_2Cl_2)

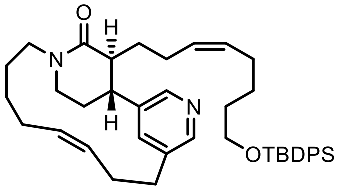

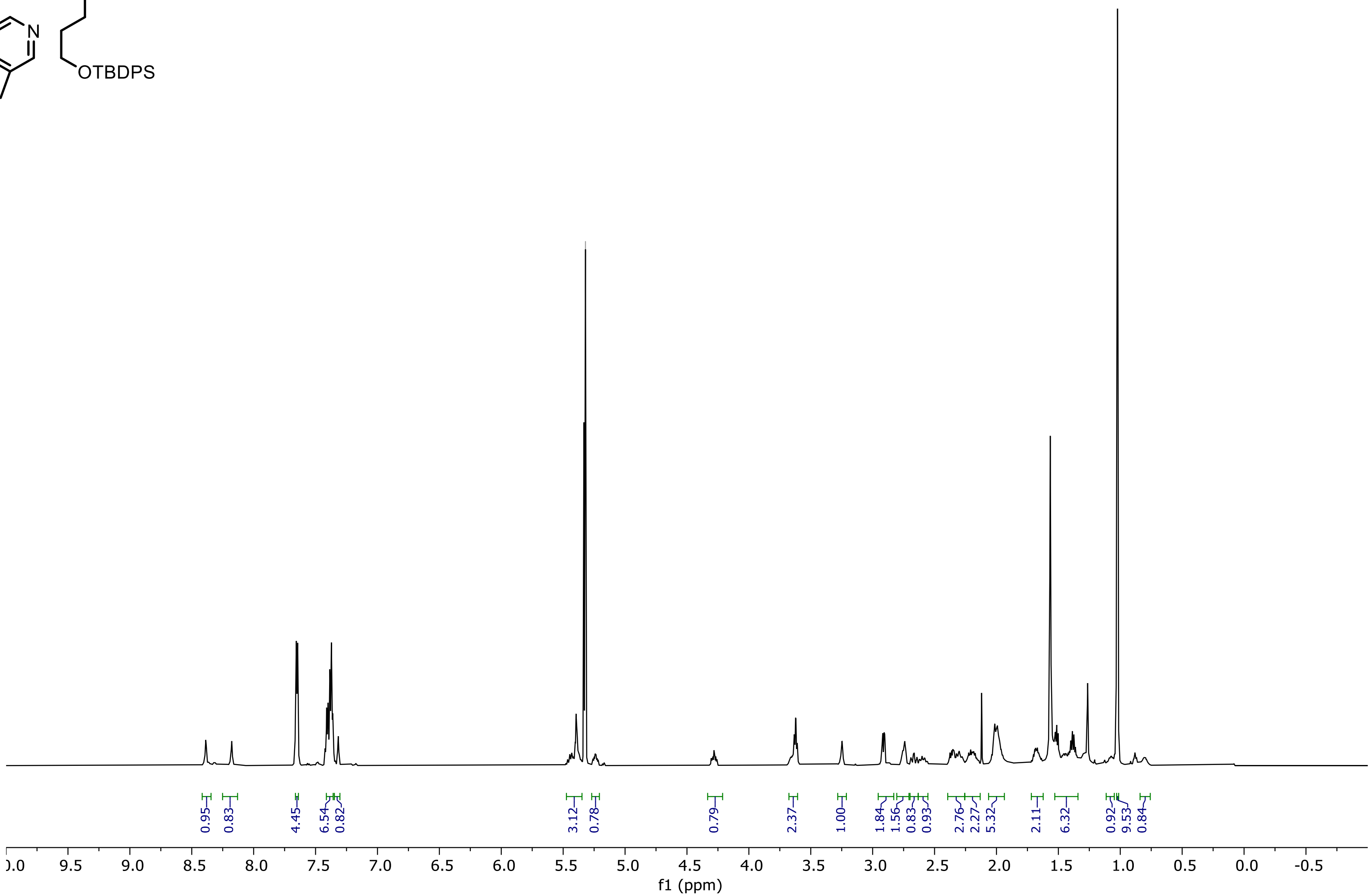

^{13}C NMR spectrum of 30 (151 MHz, CD_2Cl_2)

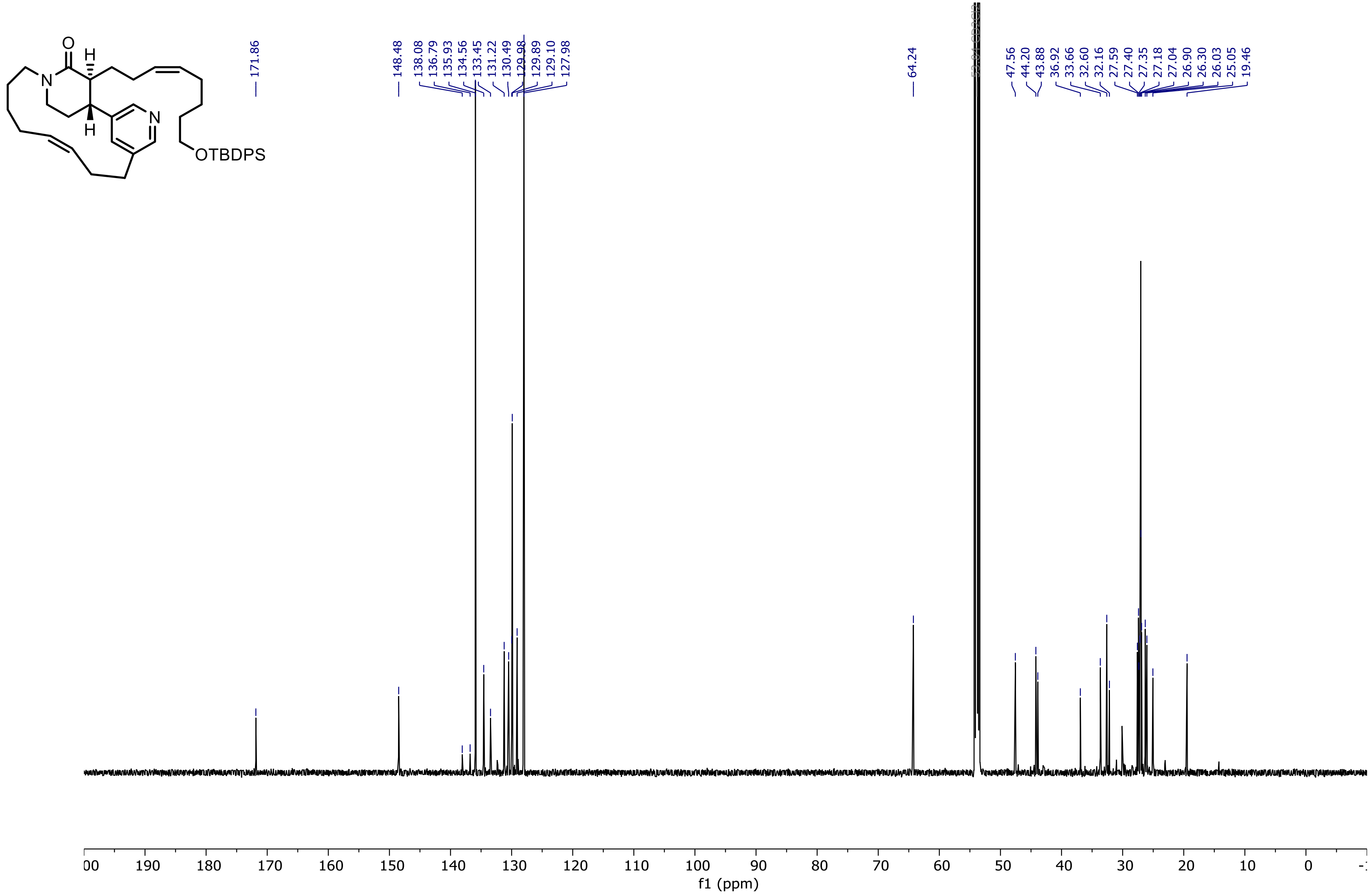

HSQC spectrum of 30

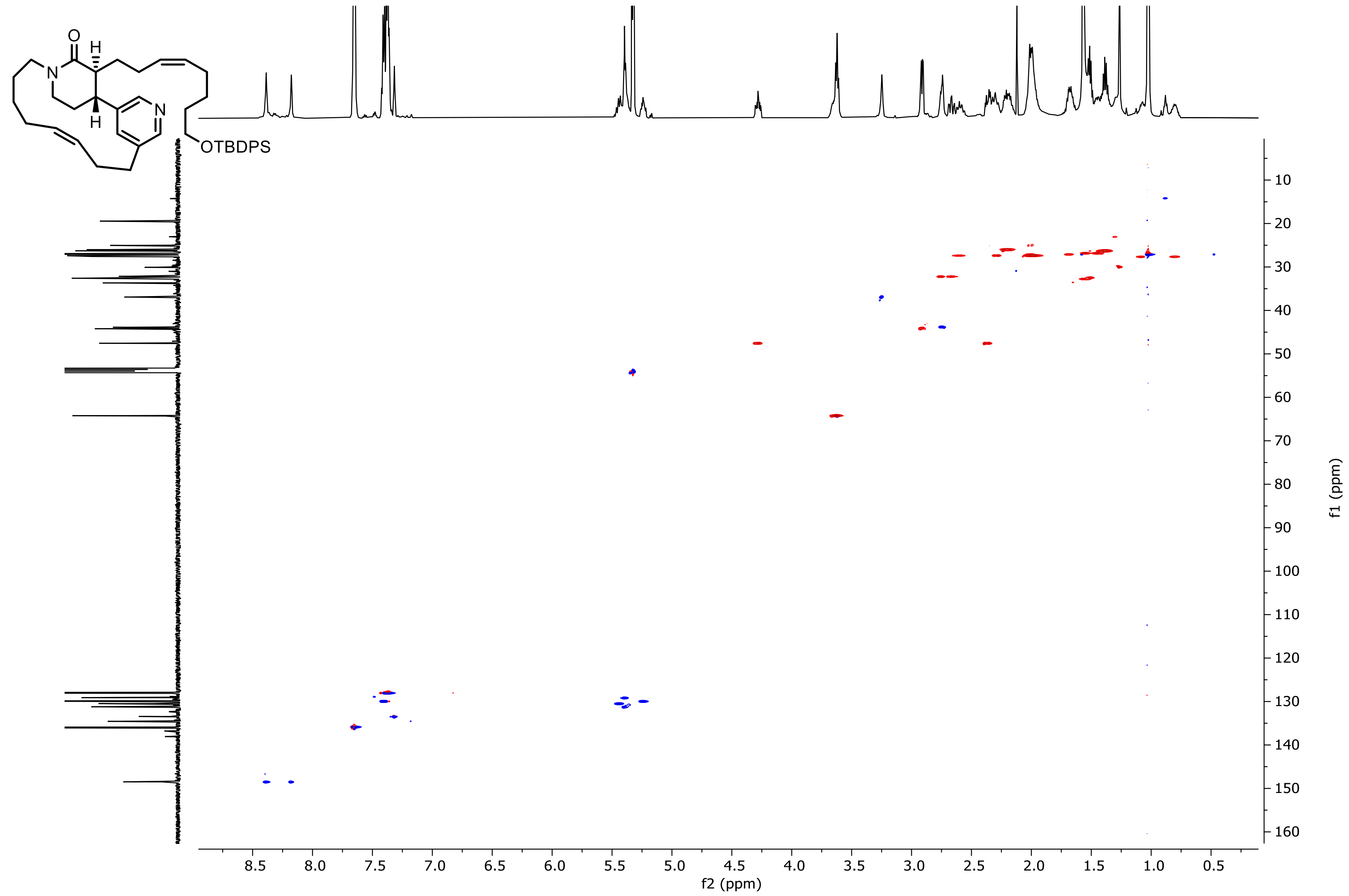

HMBC spectrum of 30

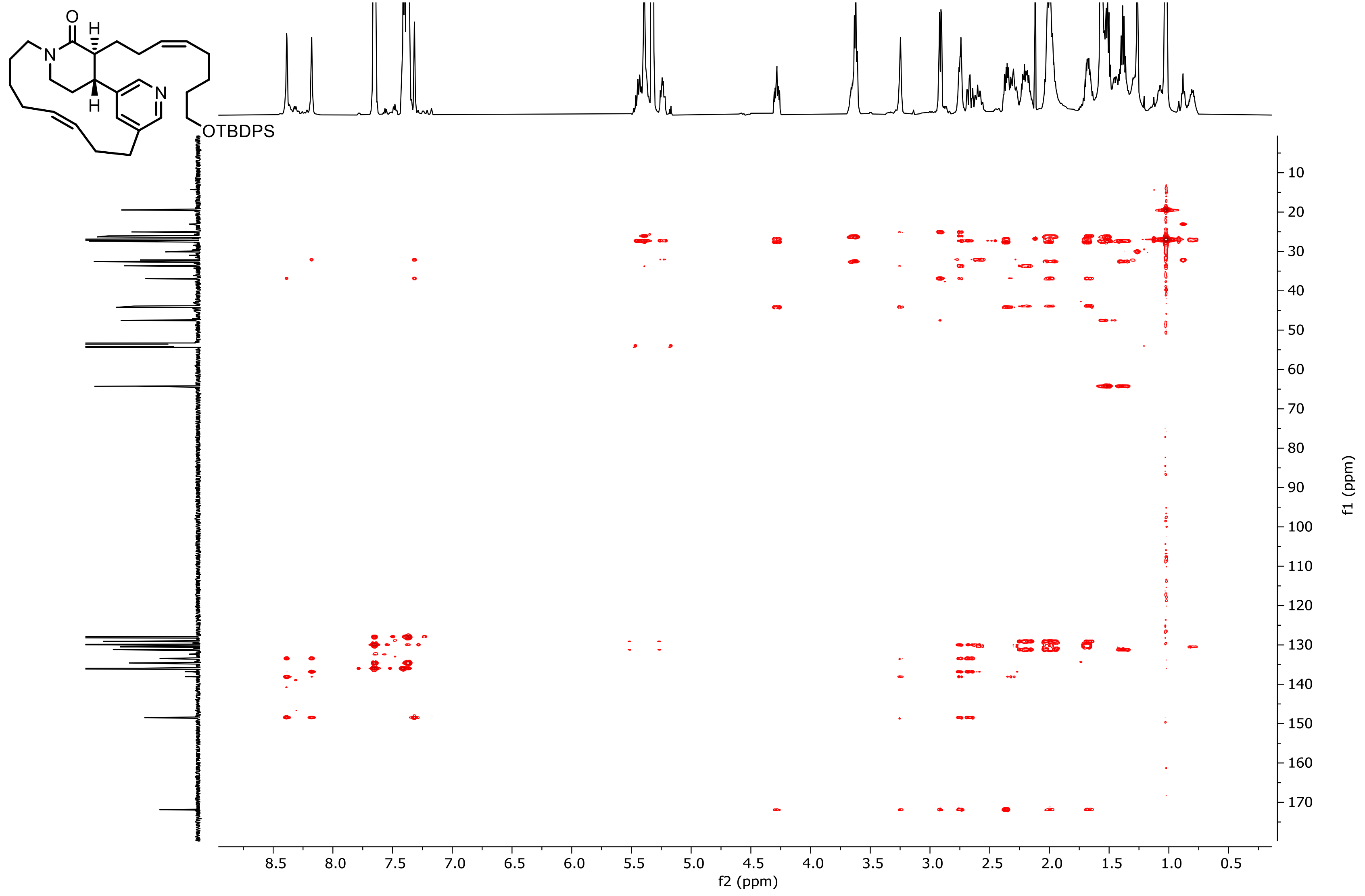

NOESY spectrum of 30

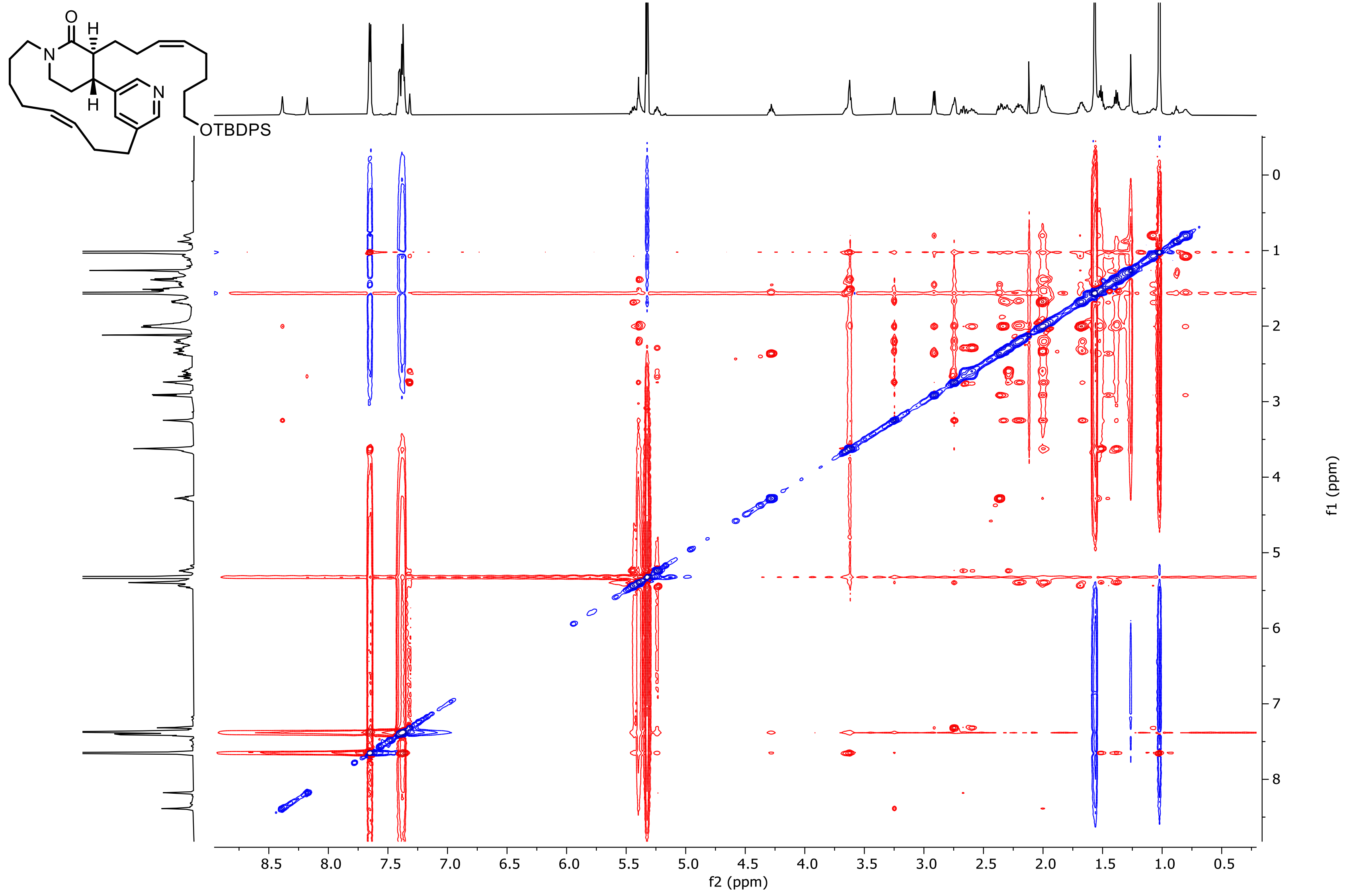

^{1}H NMR spectrum of 32 (600 MHz, CD_2Cl_2)

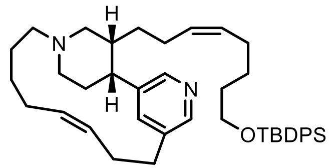

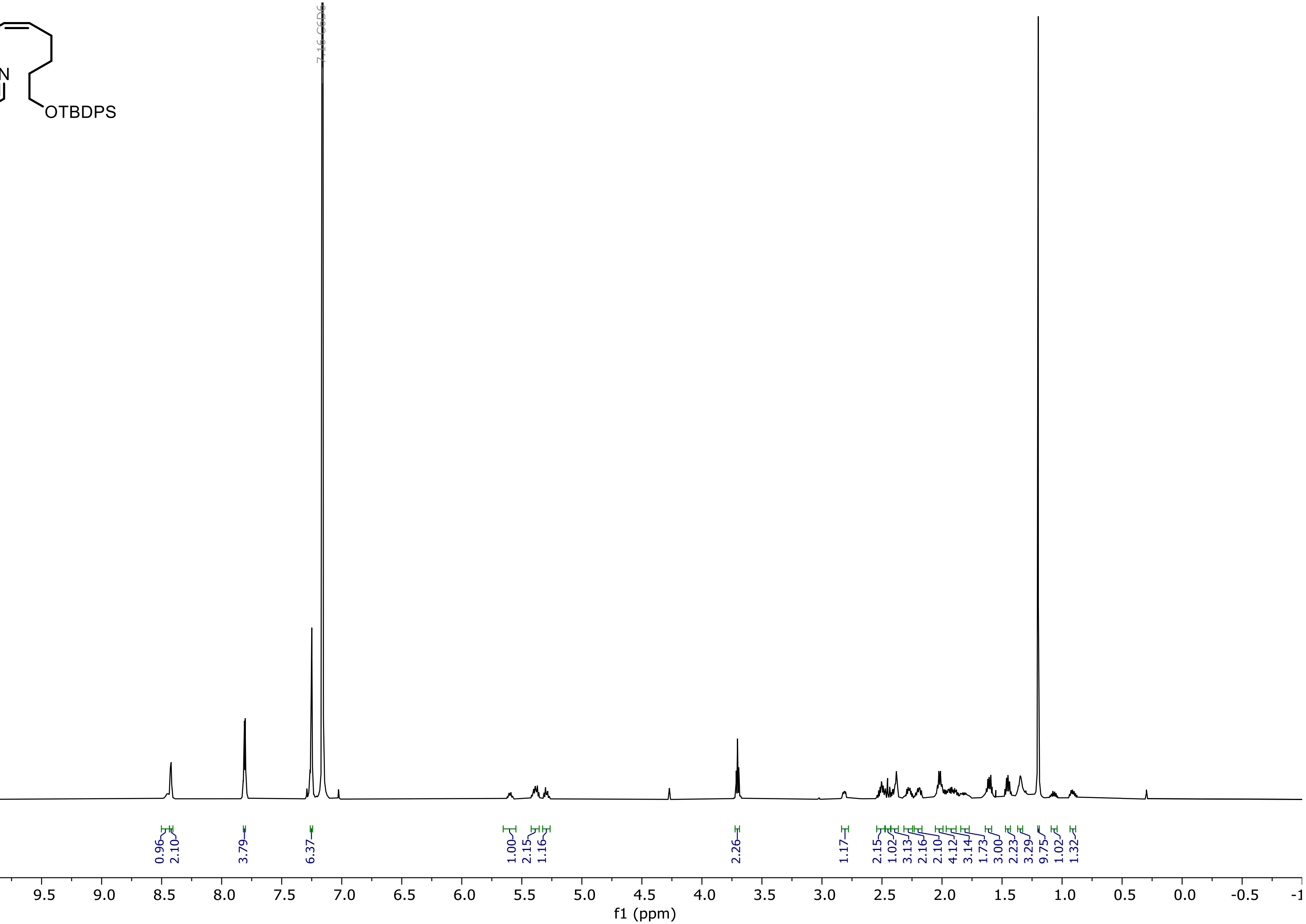

^{13}C NMR spectrum of 32 (151 MHz, CD_2Cl_2)

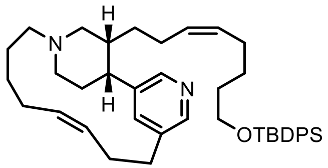

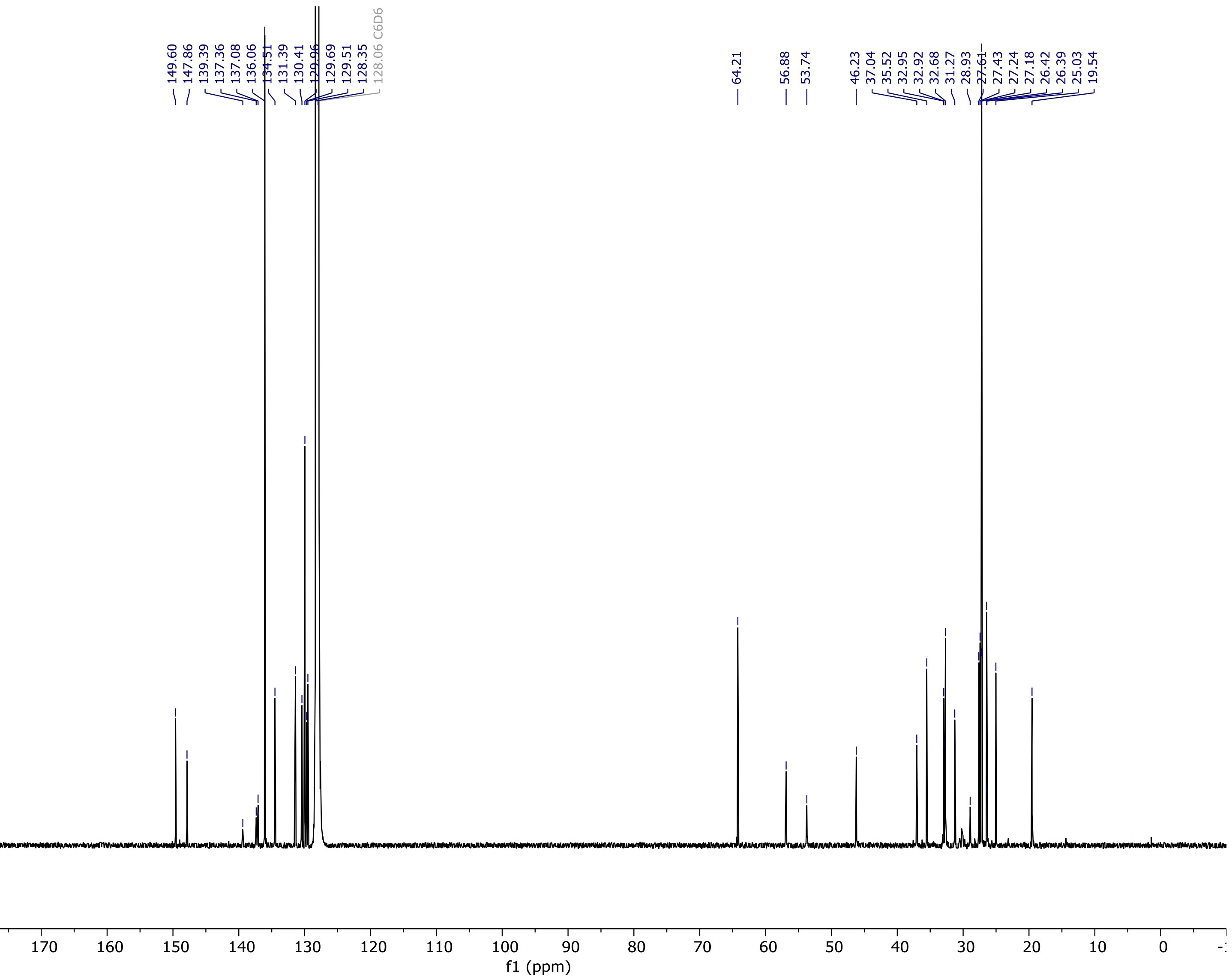

HSQC spectrum of 32

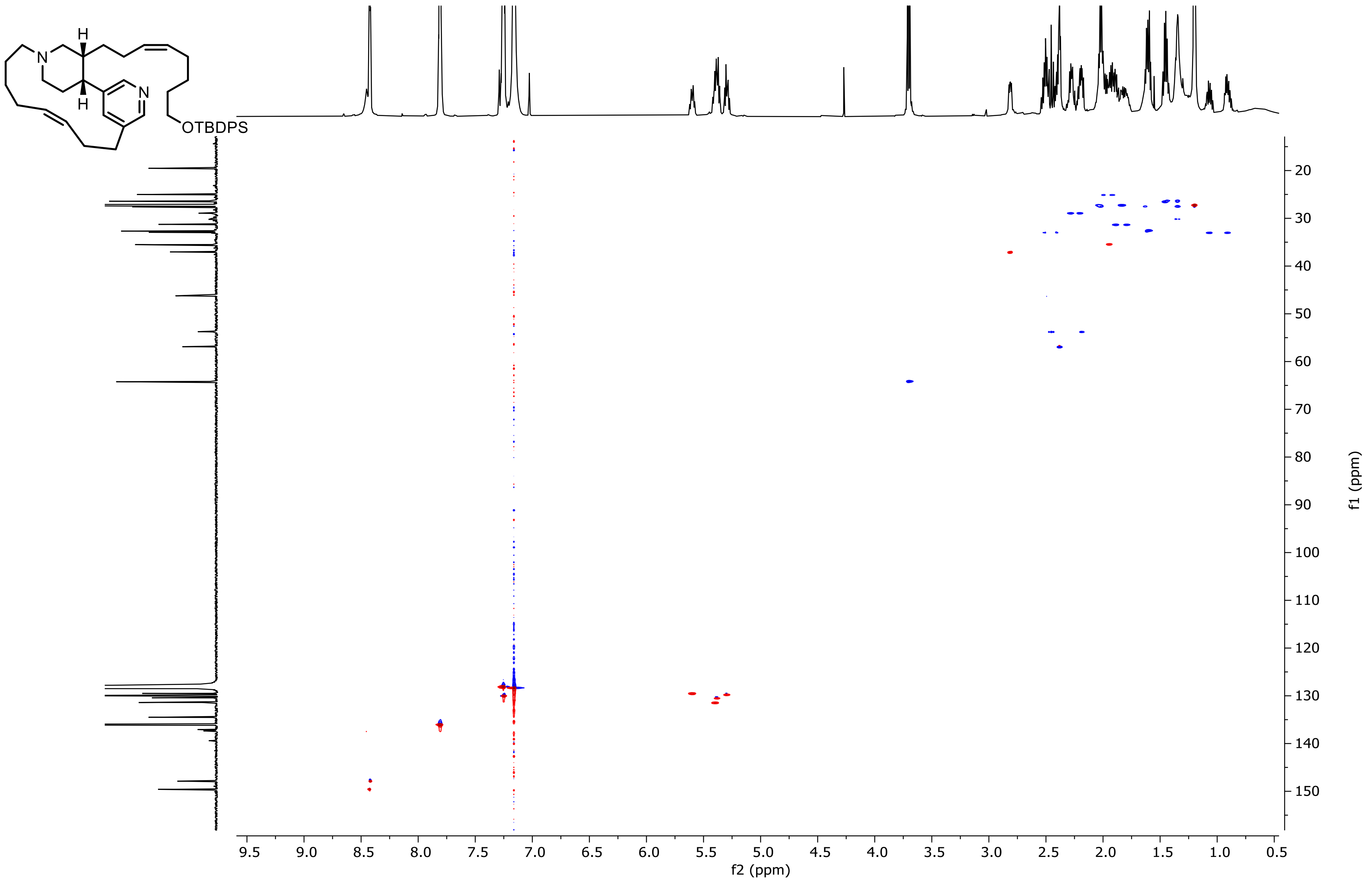

HMBC spectrum of 32

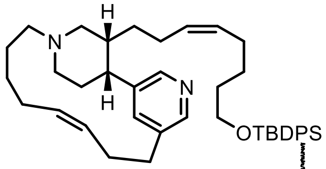

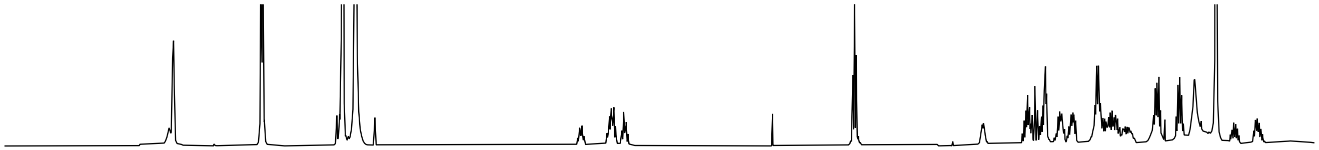

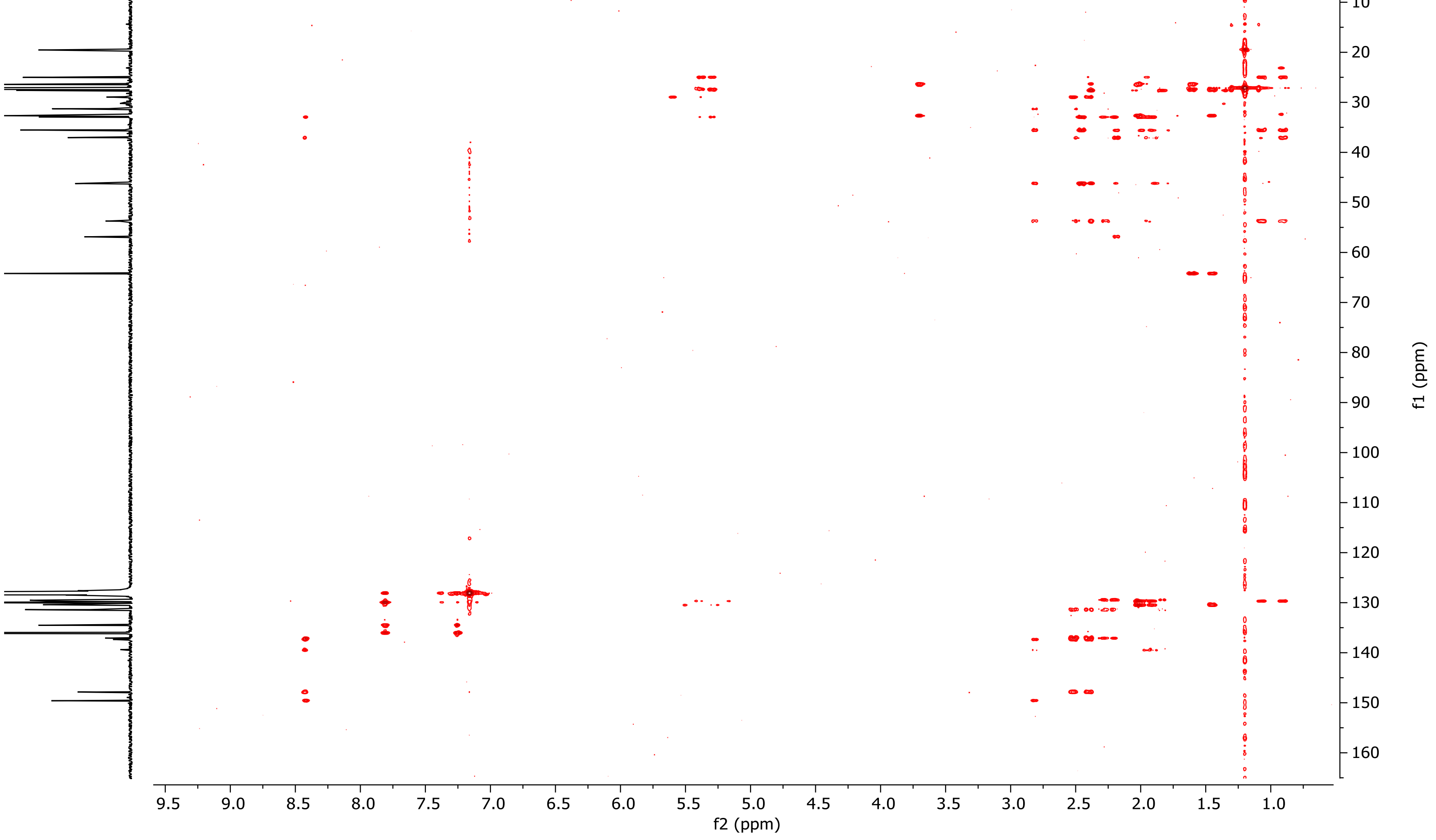

NOESY spectrum of 32

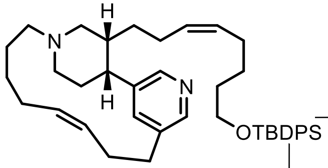

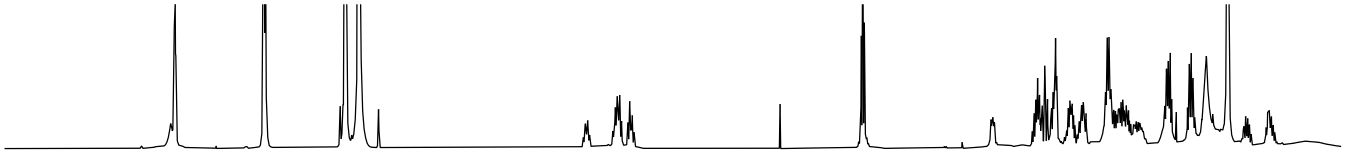

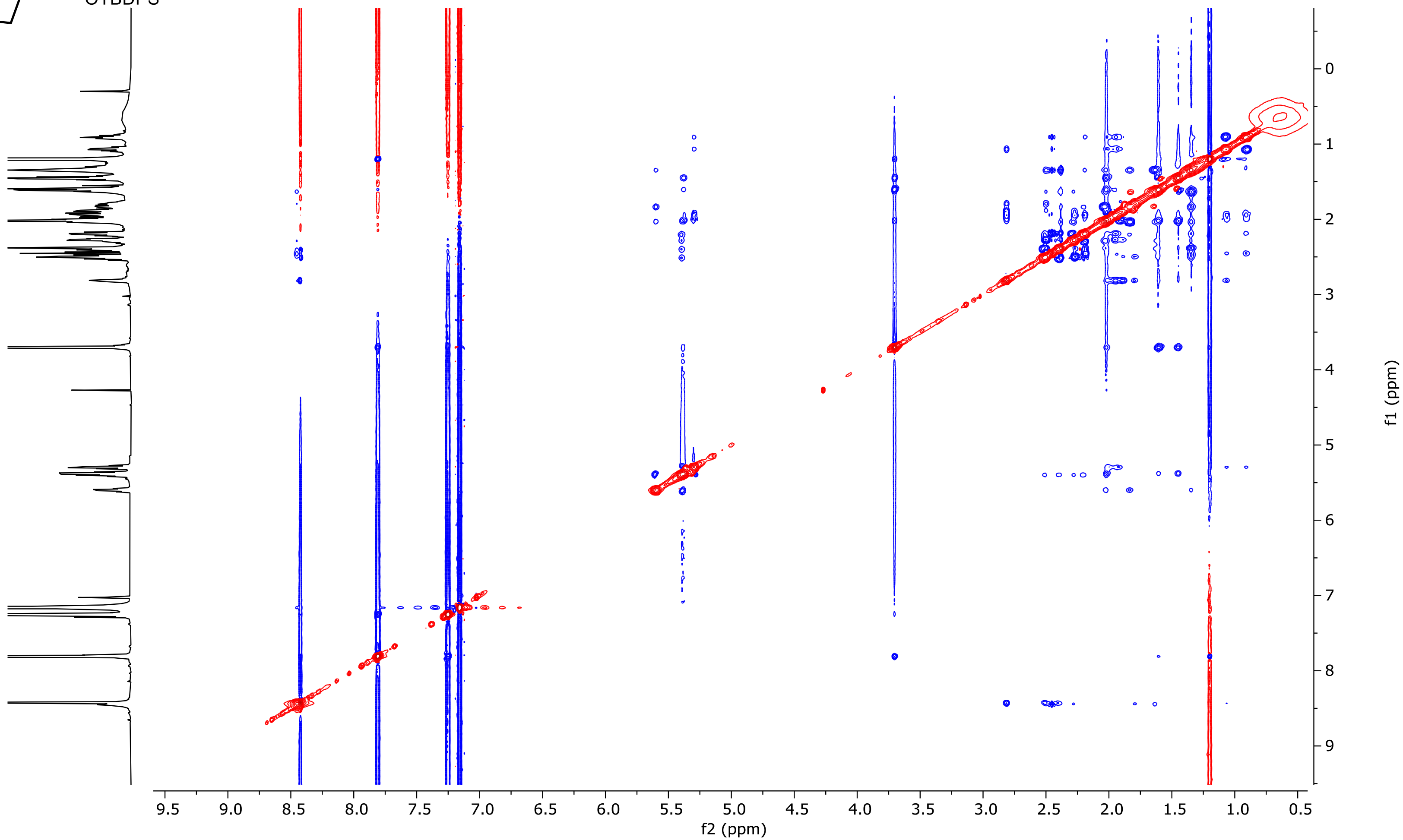

^{1}H NMR spectrum of S9 (400 MHz, CD_2Cl_2

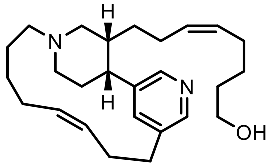

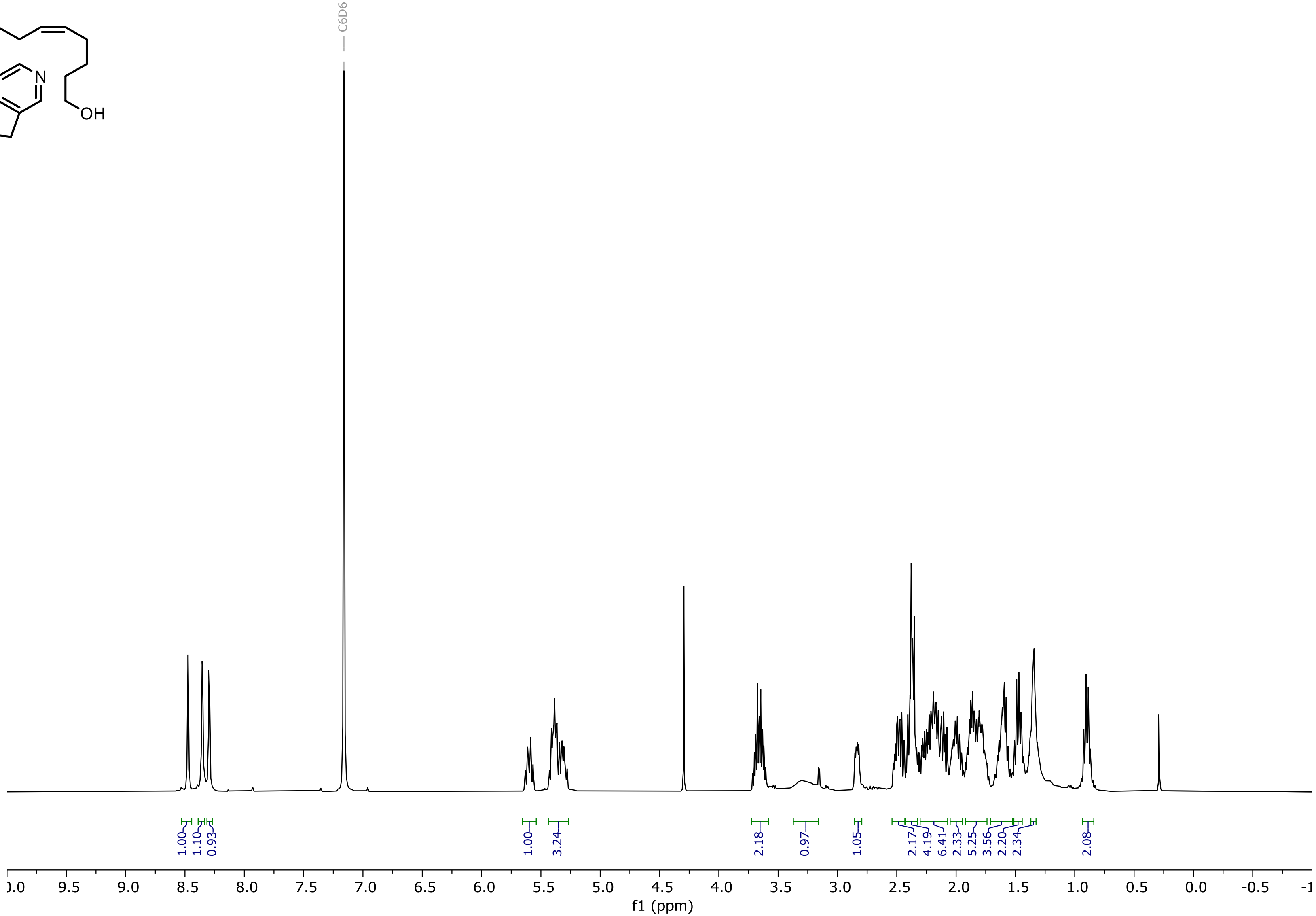

^{13}C NMR spectrum of S9 (101 MHz, CD_2Cl_2)

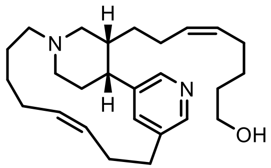

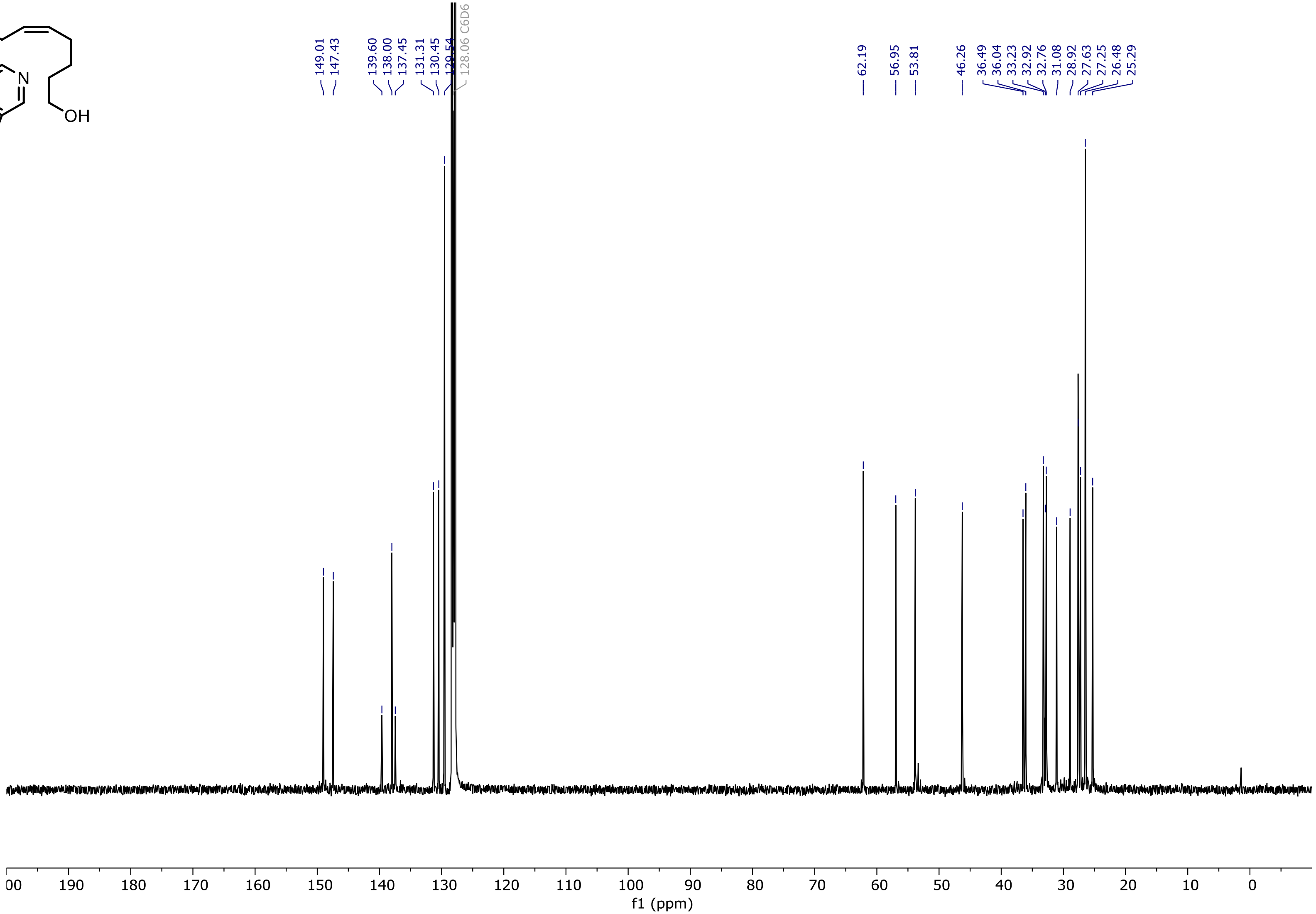

^{1}H NMR spectrum of *epi*-Tetradehydrohalicyclamine B (7) (600 MHz, D_3COD)

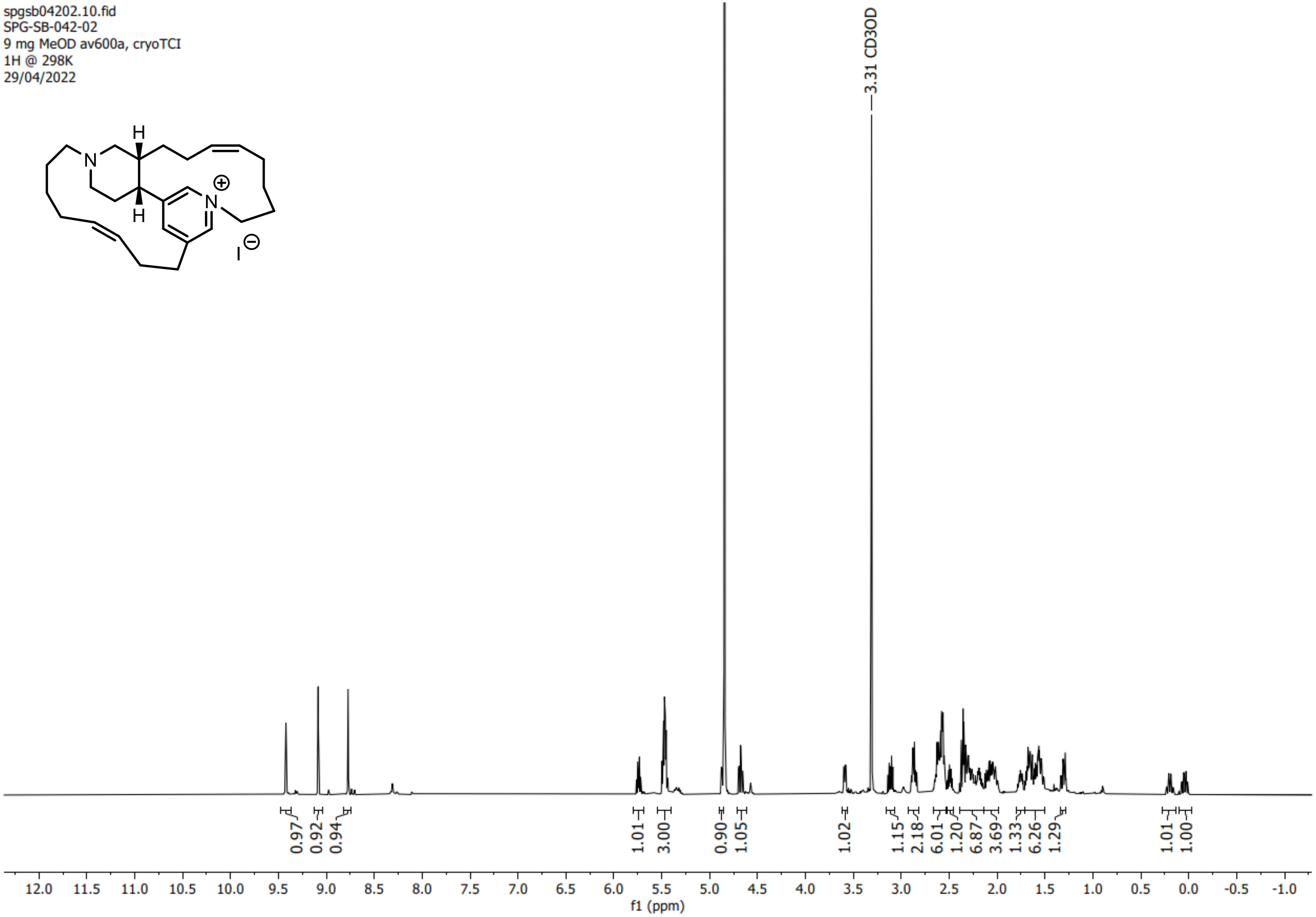

^{13}C NMR spectrum of *epi*-Tetradehydrohalicyclamine B (7) (151 MHz, D_3COD)

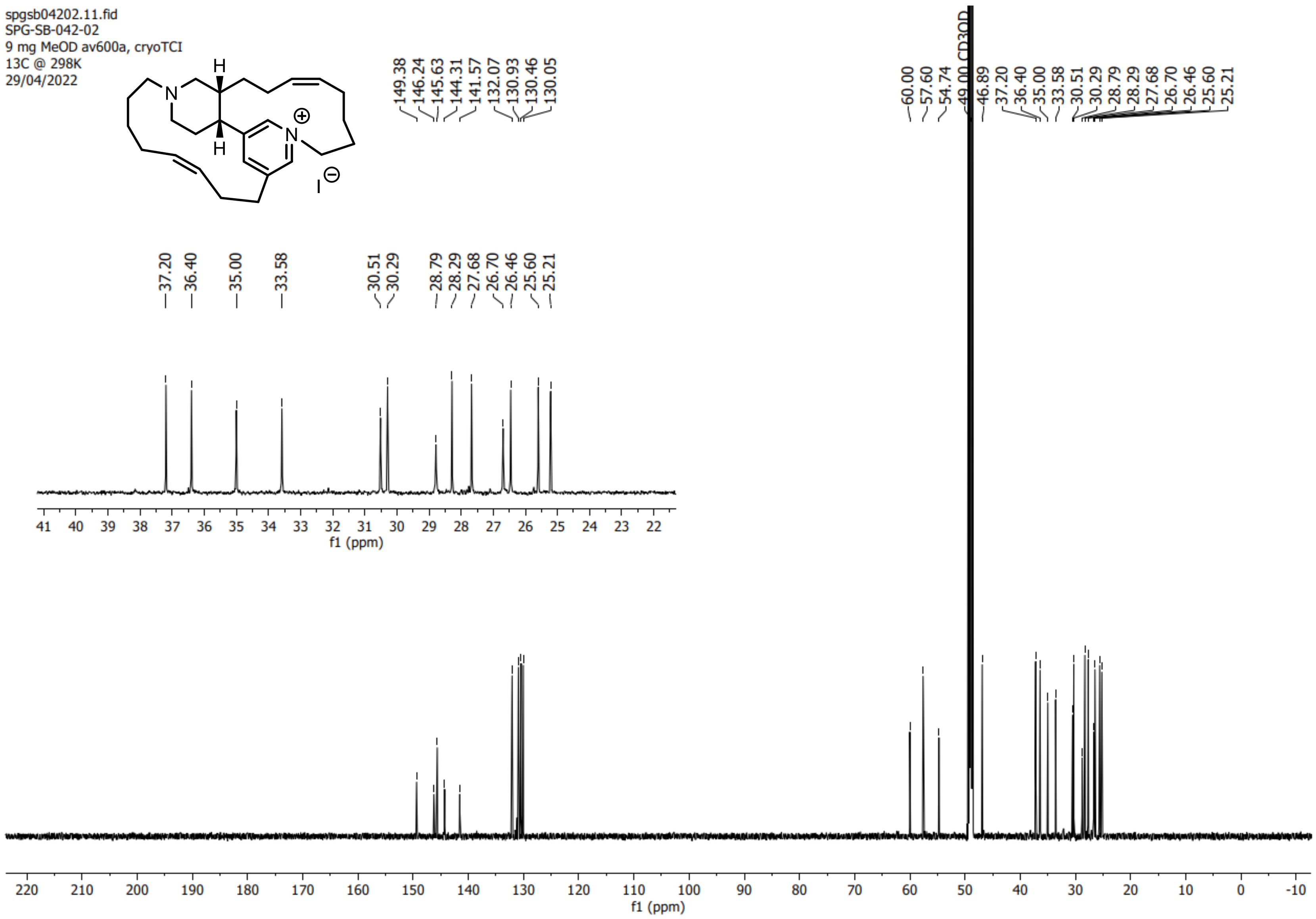

^{1}H-^{1}H COSY spectrum of *epi*-Tetradehydrohalicyclamine B (7)

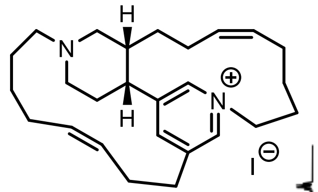

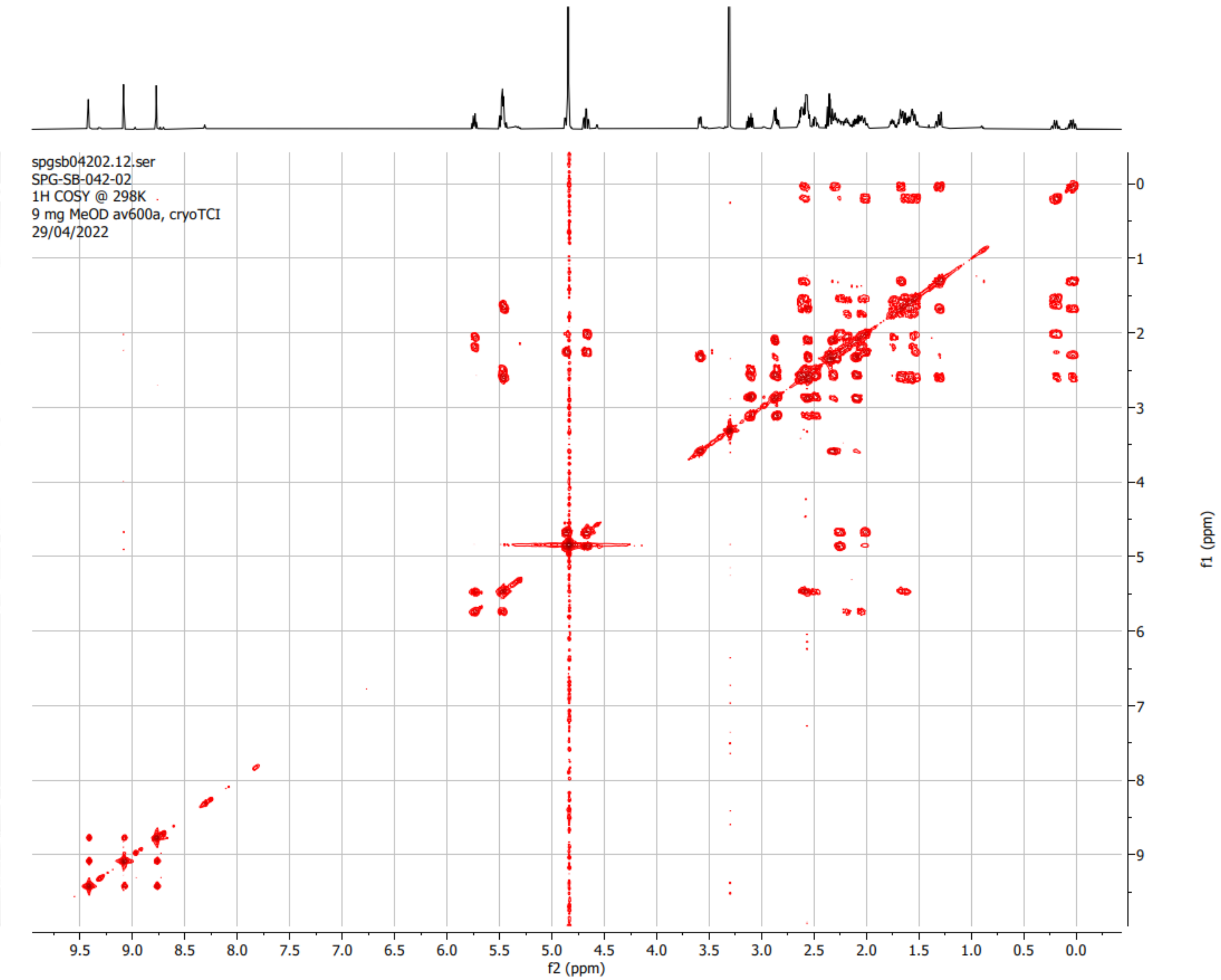

^{1}H-^{13}C HSQC spectrum of *epi*-Tetradehydrohalicyclamine B (7)

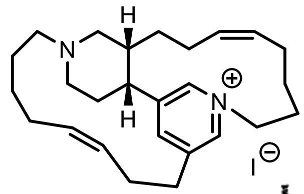

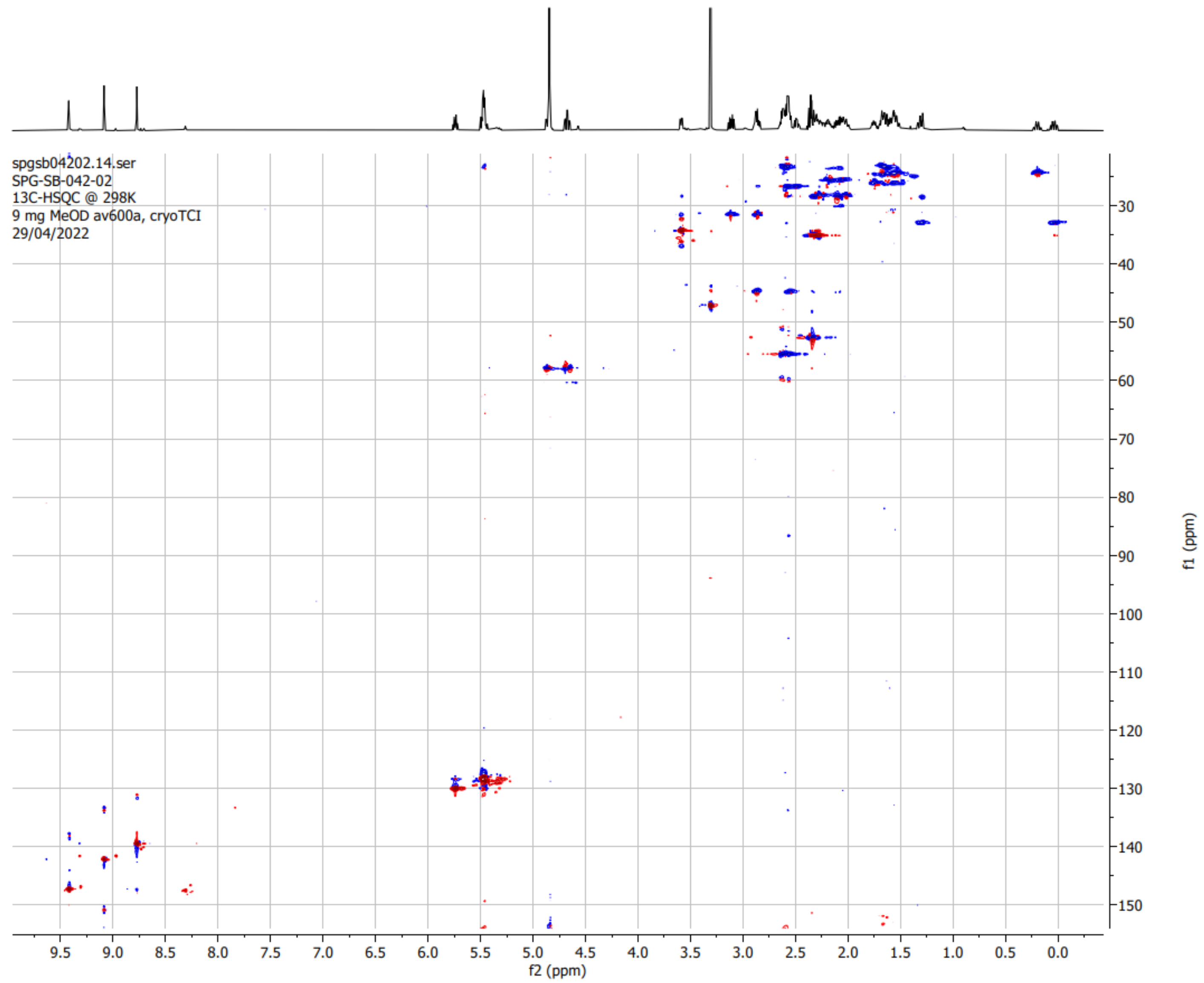

^{1}H-^{13}C **HMBC spectrum of *epi*-Tetradehydrohalicyclamine B (7)**

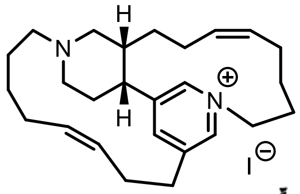

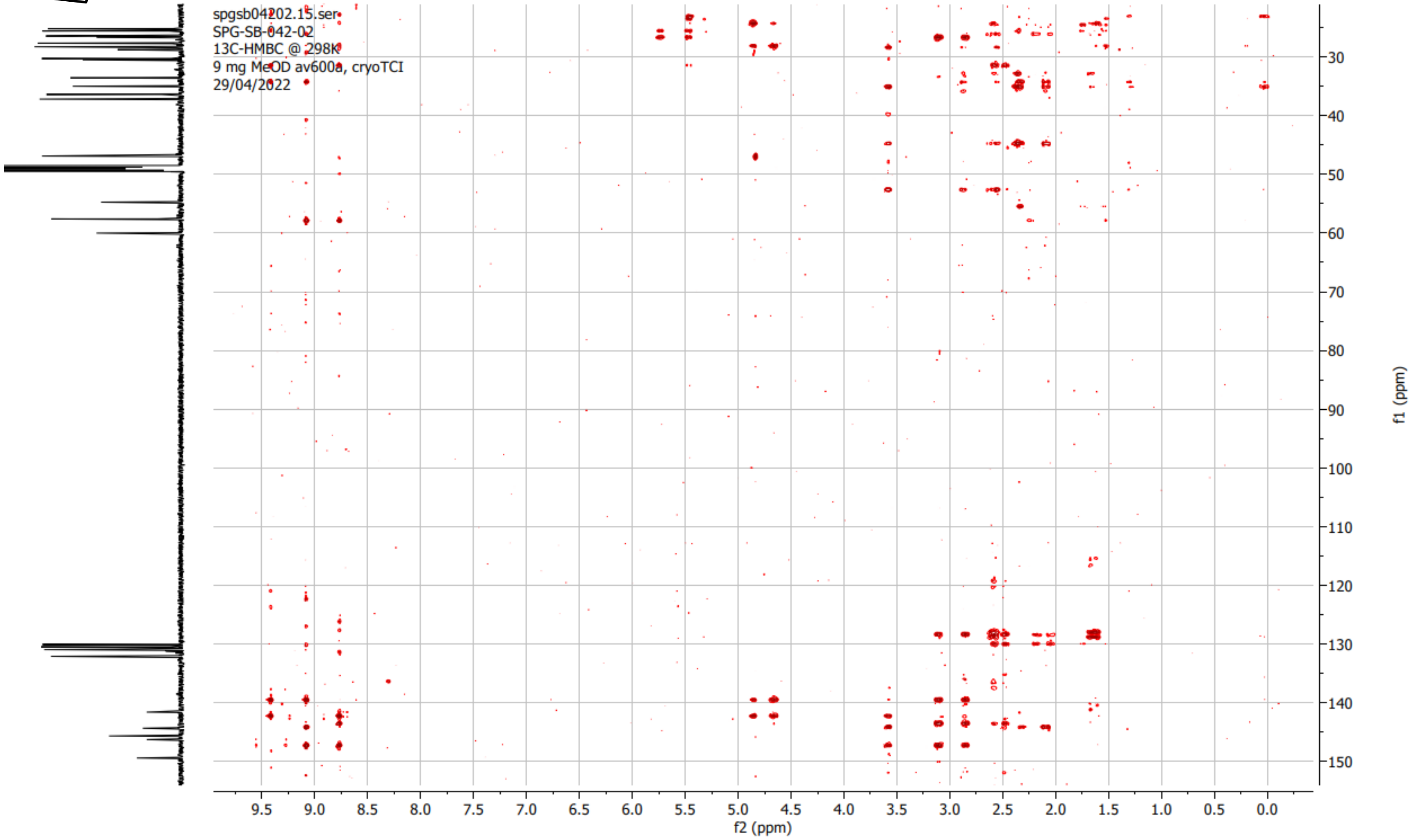

^{1}H-^{15}N HMBC spectrum of *epi*-Tetradehydrohalicyclamine B (7)

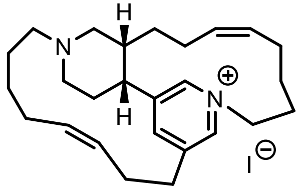

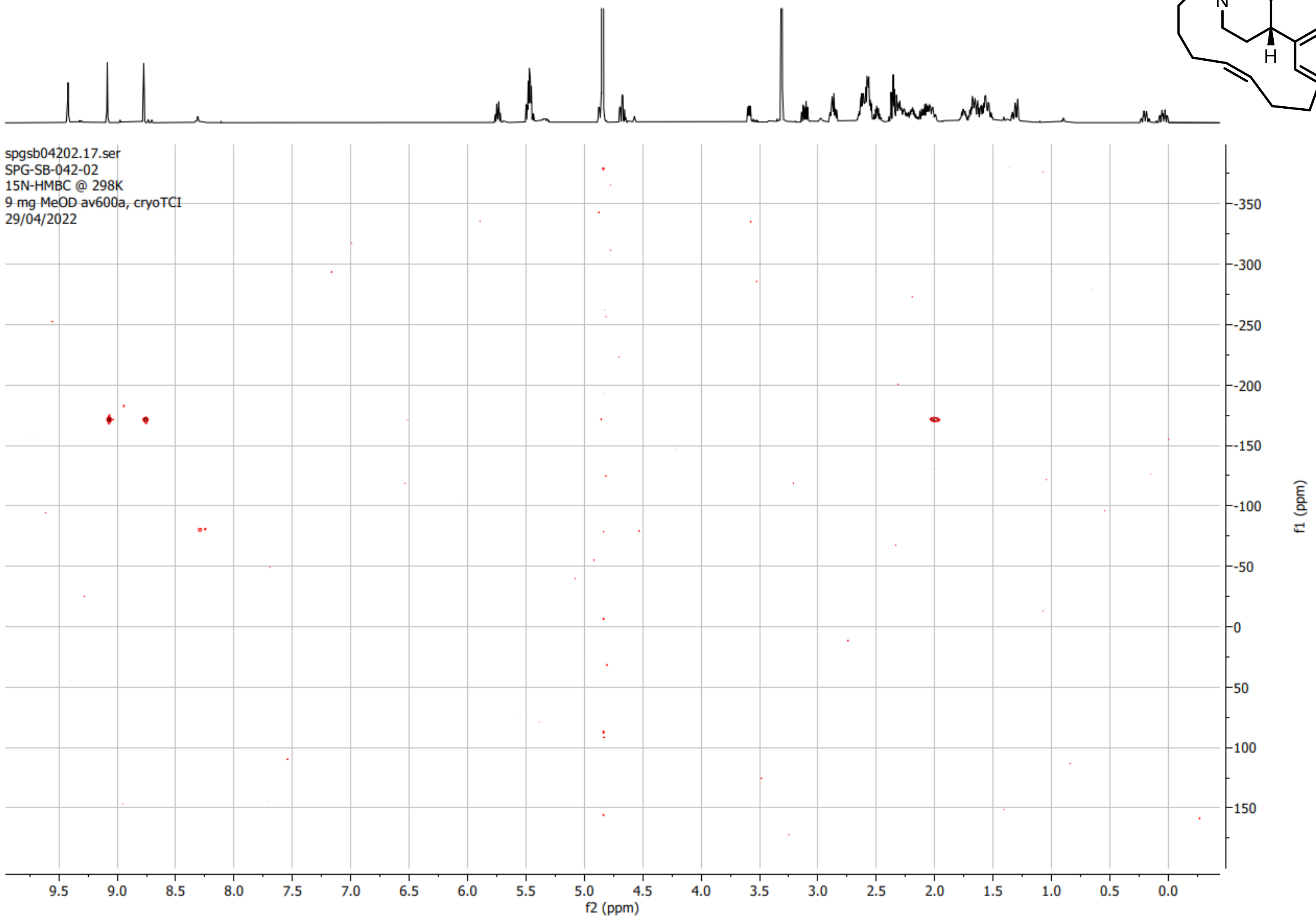

NOESY spectrum of *epi*-Tetradehydrohalicyclamine B (7)

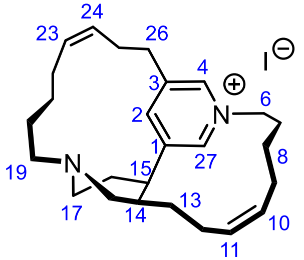

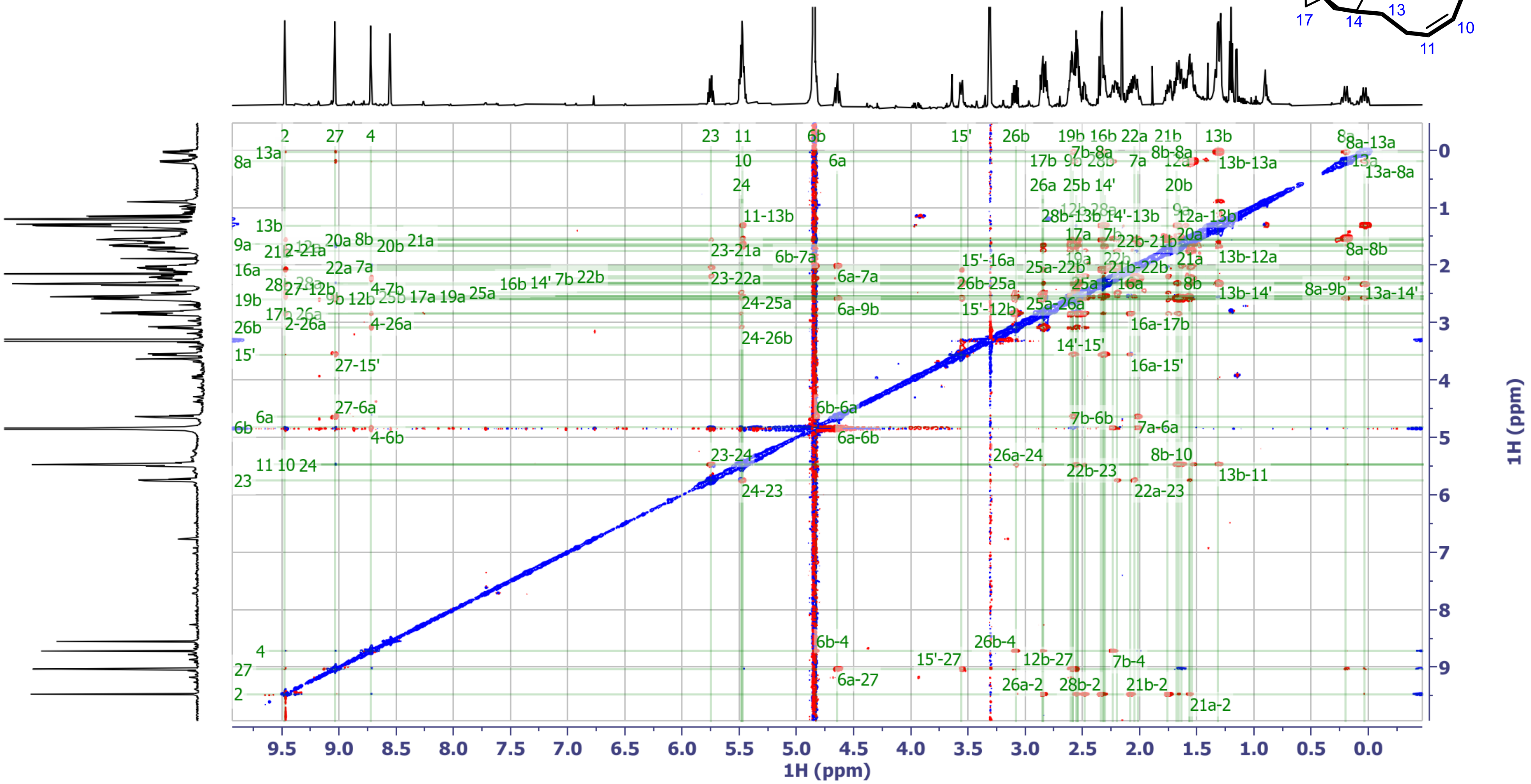

Supplement: Supplementary file 1 — Supporting Information [file ANIE-61-0-s001.pdf]
